# Carotenoid cleavage enzymes evolved convergently to generate the visual chromophore

**DOI:** 10.1038/s41589-024-01554-z

**Published:** 2024-02-14

**Authors:** Yasmeen J. Solano, Michael P. Everett, Kelly S. Dang, Jude Abueg, Philip D. Kiser

**Affiliations:** 1 Department of Physiology and Biophysics, University of California Irvine School of Medicine, Irvine, CA USA; 2 Research Service, VA Long Beach Healthcare System, Long Beach, CA USA; 3 Department of Ophthalmology, Gavin Herbert Eye Institute, University of California Irvine School of Medicine, Irvine, CA USA; 4 Department of Clinical Pharmacy Practice, University of California Irvine School of Pharmacy and Pharmaceutical Sciences, Irvine, CA USA

**Keywords:** X-ray crystallography, Enzyme mechanisms, Metalloproteins

## Abstract

The retinal light response in animals originates from the photoisomerization of an opsin-coupled 11-*cis*-retinaldehyde chromophore. This visual chromophore is enzymatically produced through the action of carotenoid cleavage dioxygenases. Vertebrates require two carotenoid cleavage dioxygenases, β-carotene oxygenase 1 and retinal pigment epithelium 65 (RPE65), to form 11-*cis*-retinaldehyde from carotenoid substrates, whereas invertebrates such as insects use a single enzyme known as Neither Inactivation Nor Afterpotential B (NinaB). RPE65 and NinaB couple *trans–cis* isomerization with hydrolysis and oxygenation, respectively, but the mechanistic relationship of their isomerase activities remains unknown. Here we report the structure of NinaB, revealing details of its active site architecture and mode of membrane binding. Structure-guided mutagenesis studies identify a residue cluster deep within the NinaB substrate-binding cleft that controls its isomerization activity. Our data demonstrate that isomerization activity is mediated by distinct active site regions in NinaB and RPE65—an evolutionary convergence that deepens our understanding of visual system diversity.

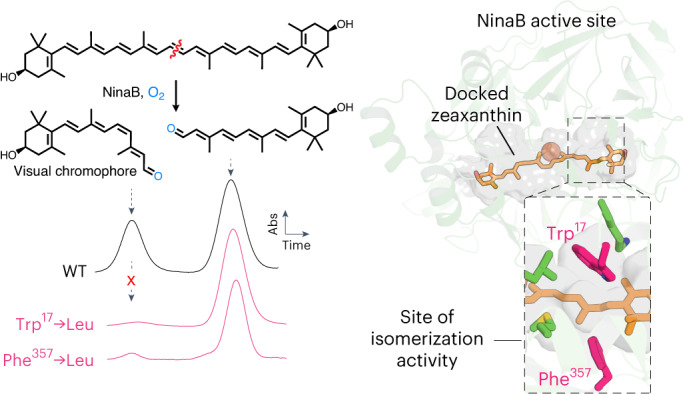

## Abstract (web summary)

NinaB is an isomerooxygenase that generates visual chromophore (11-*cis*-retinal) from carotenoid substrates. Here Solano et al. reveal the structural basis for NinaB isomerase activity, providing new insights into the evolution of visual chromophore synthesis by carotenoid cleavage enzymes.

## Main

Image-forming vision in animals starts with the photoisomerization of an opsin-linked 11-*cis*-retinaldehyde (11-*cis*-RAL) chromophore to an all-*trans* configuration^1^. This stereochemical alteration causes conformational changes in the opsin transmembrane helical bundle allowing G protein signaling and the start of phototransduction^2,3^. These events initiate electrochemical signals within retinal photoreceptor cells that are relayed to the brain through higher order neurons for interpretation. Although specific properties of the visual opsins and the modes of signaling they employ vary among animal groups^4^, the involvement of an 11-*cis*-retinoid functioning as the visual chromophore to initiate light perception is universal. In most animal groups, 11-*cis*-RAL itself is used as the visual chromophore, whereas certain fish and arthropods employ desaturated or hydroxylated 11-*cis*-RAL derivatives in their visual systems^5,6^.

Retinaldehyde (RAL) is biosynthetically generated by the oxidative cleavage of dietary carotenoid precursors, which are polyenes with multiple potentially reactive sites^7,8^. Additionally, 11-*cis*-RAL is a high-energy retinoid isomer that constitutes an inconspicuous percentage of total RAL at thermal equilibrium^9^. Ensuring the specificity of both RAL formation from carotenoids as well as its isomerization to the 11-*cis* configuration necessitates the involvement of enzymes. Iron-dependent, membrane-associated enzymes encompassed within the carotenoid cleavage dioxygenase (CCD) superfamily are pivotal in these biosynthetic transformations^7^. In vertebrates, β-carotene oxygenase 1 (BCO1) cleaves provitamin A carotenoids symmetrically to yield one or two molecules of all-*trans*-RAL. RAL is reduced to all-*trans*-retinol (vitamin A), which is trafficked to the retinal pigment epithelium (RPE) and enters the visual cycle metabolic pathway^10,11^. This pathway involves another CCD superfamily member known as RPE65, which converts all-*trans*-retinyl esters into 11-*cis*-retinol through a coupled ester hydrolysis and C11–C12 alkene isomerization reaction^12,13^. Previous biochemical and structural studies identified critical active site features in RPE65 responsible for retinoid *trans–cis* isomerase activity and ester bond hydrolysis which, together with isotope labeling data, allowed the proposal of an isomerohydrolase catalytic mechanism^14–17^.

In contrast to the multiple CCD paralogs found in vertebrates, most insect genomes encode a single CCD known as Neither Inactivation Nor Afterpotential B (NinaB)^18^. The *Drosophila melanogaster* NinaB ortholog was the initial animal CCD to be cloned and was shown to catalyze the symmetric cleavage of β-carotene to form RAL^19,20^. Later studies demonstrated that NinaB not only cleaves carotenoids symmetrically but also isomerizes one-half of its substrate to form a ~1:1 mixture of 11-*cis* and all-*trans* RAL products^21^. This enzyme was thus termed an isomerooxygenase.

It is intriguing that two homologous enzymes catalyzing distinct primary chemistry on disparate substrates—retinyl ester hydrolysis in the case of RPE65 and carotenoid oxygenation in the case of NinaB—both possess the ability to catalyze *trans–cis* isomerization at a C11–C12 retinoid/carotenoid double bond. This finding raises the question of whether the common ancestor of the lineages leading to the NinaB and RPE65 proteins, which existed greater than 550 million years ago^22^, also possessed *trans–cis* isomerase activity as previously suggested^21,23^. If true, the catalytic machinery responsible for isomerase activity in RPE65 and NinaB is expected to be conserved. Indeed, some residues of known importance for RPE65 isomerase activity align with identical residues in NinaB^23^. Alternatively, isomerase activity may have arisen independently in the two lineages given the fundamental differences in their primary enzymatic activities. Distinguishing between these two possibilities has important implications for our understanding of the early evolution of visual opsins in animals and the mechanisms available for the regeneration of their visual chromophores. The lack of activity data on CCDs from early branching protostomes and deuterostomes precludes phylogenetic and ancestral character state reconstruction approaches to addressing the question. The more direct route of comparing mechanistic relationships between RPE65 and NinaB has been hampered by a paucity of knowledge regarding the structure of the NinaB catalytic site.

To bridge this gap in understanding, we report a high-resolution crystal structure of NinaB from the cabbage looper (*Trichoplusia ni*), revealing the molecular architecture of its active site and details of its membrane-interacting structural elements. Employing a structure-guided mutagenesis approach, we pinpoint the site responsible for NinaB isomerase activity within its expansive substrate-binding cleft. Comparison with the site of isomerization known for RPE65 indicates a functional convergence in the evolution of CCD isomerase activity for visual chromophore biosynthesis.

## Results

### Identification of robustly expressed NinaB orthologs

**Extended Data Fig. 1:**
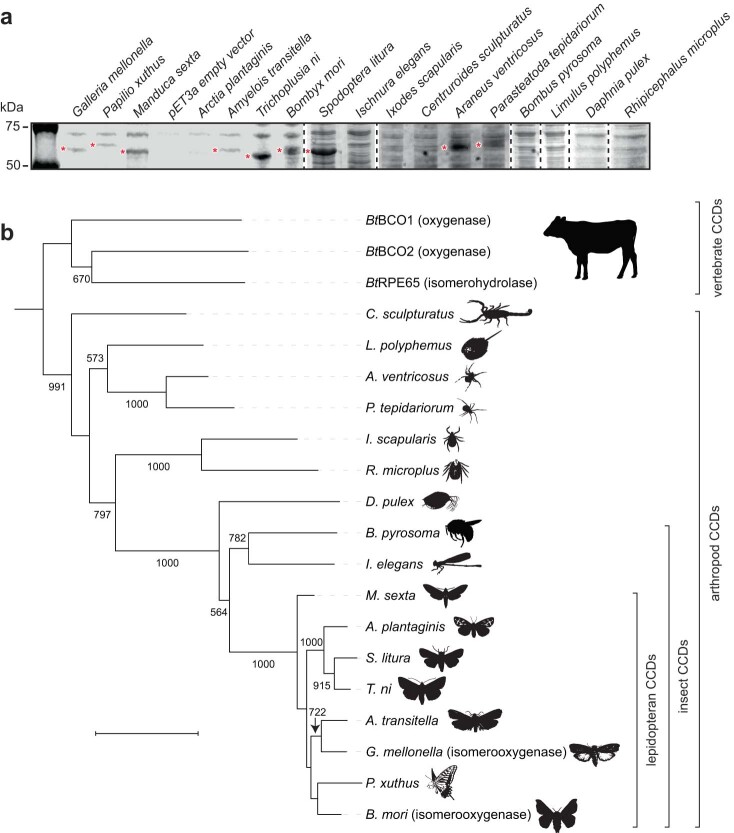
NinaB homologs investigated in this study. **a**, Coomassie-stained SDS-PAGE gels showing the level of soluble expression of each homolog. Bands representing the expressed CCD are marked with red asterisks. The dashed lines demarcate individual gels. Each test expression was performed once. See also Supplementary Data Table 1. **b**, Maximum-likelihood phylogeny of arthropod NinaB homologs investigated in this study along with vertebrate CCDs from *Bos taurus* (Bt). Enzymatic activities known prior to the present study are shown in parentheses. The tree was computed based on aligned amino acid sequences under the LG +G+I model of evolution with four rate categories. Numbers at bipartitions are bootstrap values obtained from 1,000 pseudoreplicates. Only values > 500 are shown. The tree scale bar (lower left black line) denotes an average of one expected substitution per site. Source data

**Extended Data Fig. 2:**
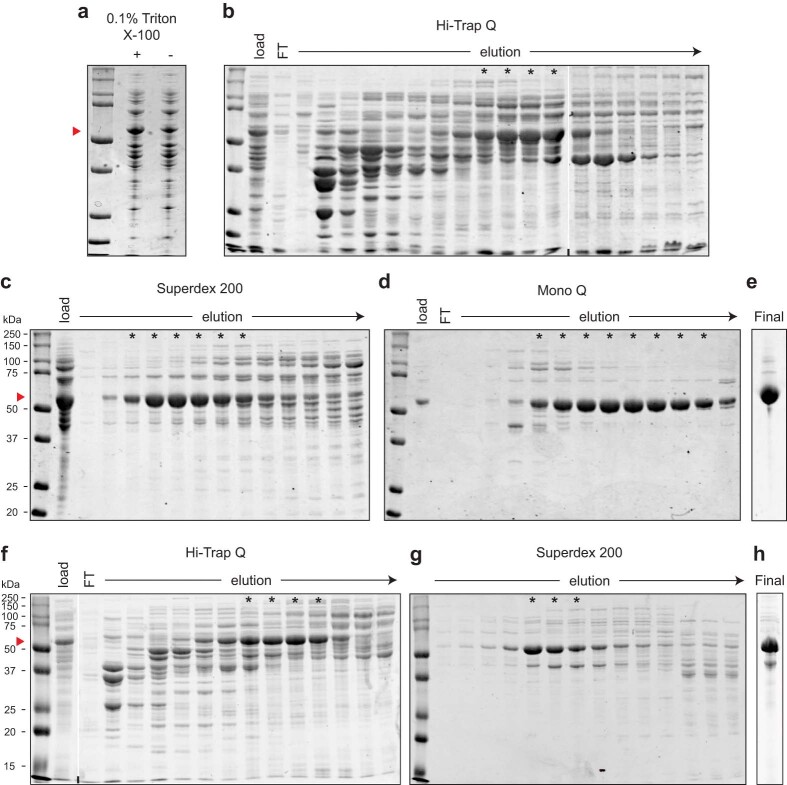
Expression and purification of *Tn*NinaB and *Sl*NinaB as analyzed by SDS-PAGE. **a**, Impact of Triton X-100 on the yield of soluble *Tn*NinaB. The red arrowhead denotes the position of the NinaB band at ~57 kDa. Purification of *Tn*NinaB by **b**, strong anion-exchange chromatography, **c**, size-exclusion chromatography, and **d**, high-resolution anion exchange chromatography. **e**, Final *Tn*NinaB preparation used for crystallization and activity studies. **f**, Purification of *Sl*NinaB by strong anion-exchange chromatography. **g**, Purification of *Sl*NinaB by size-exclusion chromatography. **h**, Final *Sl*NinaB preparation used for crystallization and activity studies. Asterisks indicate fractions that were pooled for the next purification step. FT – flow-through. All gels were stained with Coomassie R250. Vertical lines in b and f indicate the borders of separate gels. The gels are representative of five independent purifications for *Tn*NinaB and two independent purifications in the case of *Sl*NinaB. Source data

The NinaB ortholog from *Galleria mellonella* (*Gm*NinaB) was the first functionally characterized isomerooxygenase enzyme, and its substrate specificity has been extensively defined^21,23^. However, this protein has not proved amenable for structural characterization. To identify a NinaB ortholog suitable for high-resolution structural analysis, we performed an *Escherichia coli* expression screen of putative NinaB proteins from a variety of insects and other arthropods (Supplementary Table 1 and Extended Data Fig. 1). NinaB proteins from the cabbage looper (*T. ni*, *Tn*NinaB) and tobacco cutworm (*Spodoptera litura*, *Sl*NinaB*)* exhibited particularly high soluble expression and were chosen for further analysis. Addition of detergent (Triton X-100) to the cell lysate improved the yield of soluble NinaB protein, consistent with the known membrane affinity of CCDs^24^ (Extended Data Fig. 2a). We chromatographically purified the solubilized NinaB proteins, which resulted in nearly homogeneous preparations (Extended Data Fig. 2b–h).

**Fig. 1:**
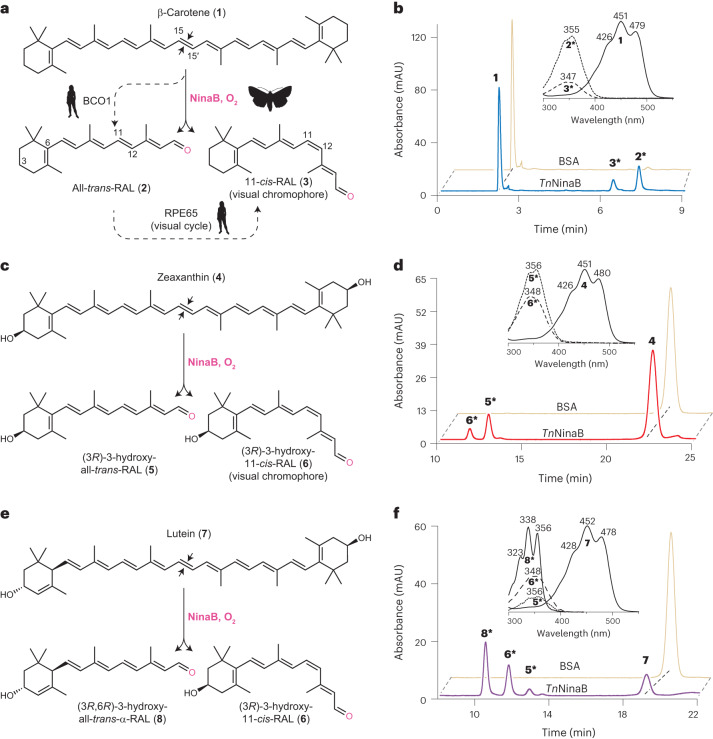
Enzymatic activity of *Tn*NinaB toward carotenoid and xanthophyll substrates. **a**, NinaB isomerooxygenases found in insects (represented by the moth silhouette) cleave β-carotene (**1**) to generate all-*trans*-RAL (**2**) and the visual chromophore 11-*cis*-RAL (**3**). In vertebrates (represented by the human silhouette), 11-*cis*-RAL biosynthesis requires two separate CCD enzymes (BCO1 and RPE65). **b**, HPLC chromatograms demonstrating that *Tn*NinaB displays isomerooxygenase activity toward β-carotene. **c**, Schematic of NinaB activity towards zeaxanthin (**4**) generating (3*R*)-3-hydroxy-all-*trans*-RAL (**5**) and (3*R*)-3-hydroxy-11-*cis*-RAL (**6**), the latter serving as the visual chromophore in insects. **d**, HPLC chromatograms demonstrating that *Tn*NinaB displays isomerooxygenase activity toward zeaxanthin. **e**, Schematic of NinaB activity toward the asymmetric xanthophyll, lutein (**7**), generating (3*R*,6*R*)-3-hydroxy-all-*trans*-α-RAL (**8**) and (3*R*)-3-hydroxy-11-*cis*-RAL (**6**). **f**, HPLC chromatograms demonstrating that *Tn*NinaB displays isomerooxygenase activity toward lutein. BSA was used as a negative control for the assays. RAL products were converted into oxime derivatives before HPLC analysis, which is indicated by asterisks next to compound numbers in **b**, **d** and **f**. Chromatograms were recorded at a wavelength of 360 nm. Insets in **b**, **d** and **f** show absorbance spectra for each of the labeled peaks confirming their identities. Numbers above spectral maxima are in nanometers. The data are representative of three replicates. Source data

**Extended Data Fig. 3:**
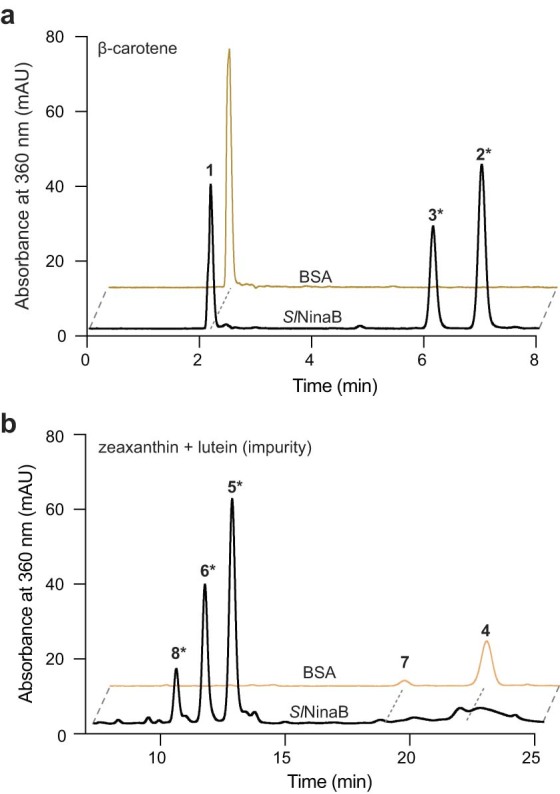
*Sl*NinaB activity assays. **a**, *Sl*NinaB forms the all-*trans* (**2**) and 11-*cis* (**3**) isomers of RAL from β-carotene (**1**). **b**, *Sl*NinaB forms the all-*trans* (**5**) and 11-*cis* (**6**) and isomers of (3*R*)-3-hydroxy-RAL from zeaxanthin (**4**). Additionally, (3*R*,6*R*)-3-hydroxy-all-*trans*-α-RAL (**8**) was formed from contaminating lutein (**7**) present in the substrate stock. RAL products were converted into oxime derivatives prior to HPLC analysis, which is indicated by asterisks next to compound numbers. BSA was used as a negative control for these assays. The data are representative of three independent experiments. Source data

Next, we characterized the enzymatic activity of the purified proteins towards carotene and xanthophyll substrates (Fig. 1). Assays performed with β-carotene (**1**) and zeaxanthin (**4**) revealed that *Tn*NinaB and *Sl*NinaB generated both all-*trans*-RAL (**2**) and 11-*cis*-RAL (**3**) or the corresponding (3*R*)-3-hydroxy derivatives (compounds **5** and **6**) at a ratio of ~0.8 to 1, similar to the 1:1 ratio reported for *Gm*NinaB^21^ (Fig. 1a–d and Extended Data Fig. 3). Enzymatic tests of *Tn*NinaB activity towards the asymmetric xanthophyll, lutein (**7**), revealed cleavage and preferential isomerization of the β-ring side of the substrate to yield (3*R*,6*R*)-3-hydroxy-all-*trans*-α-RAL (**8**) and compound **6** (Fig. 1e,f), similar to the behavior observed for *Gm*NinaB^21^. These data demonstrate *Tn*NinaB and *Sl*NinaB are bona fide isomerooxygenases that functionally recapitulate the substrate and isomerization specificities observed for *Gm*NinaB.

### NinaB crystal structure reveals active site specialization

Next, we generated crystals of *Tn*NinaB that diffracted synchrotron X-rays to ~1.95-Å resolution. The structure was solved by molecular replacement in space group *C*2, revealing an asymmetric unit composed of eight copies of *Tn*NinaB. Iterative refinement and validation produced a structural model characterized by an *R*-factor computed with reflections excluded from refinement (*R*_free_) equal to 24.7% with sound geometrical quality (Supplementary Table 2).

**Fig. 2:**
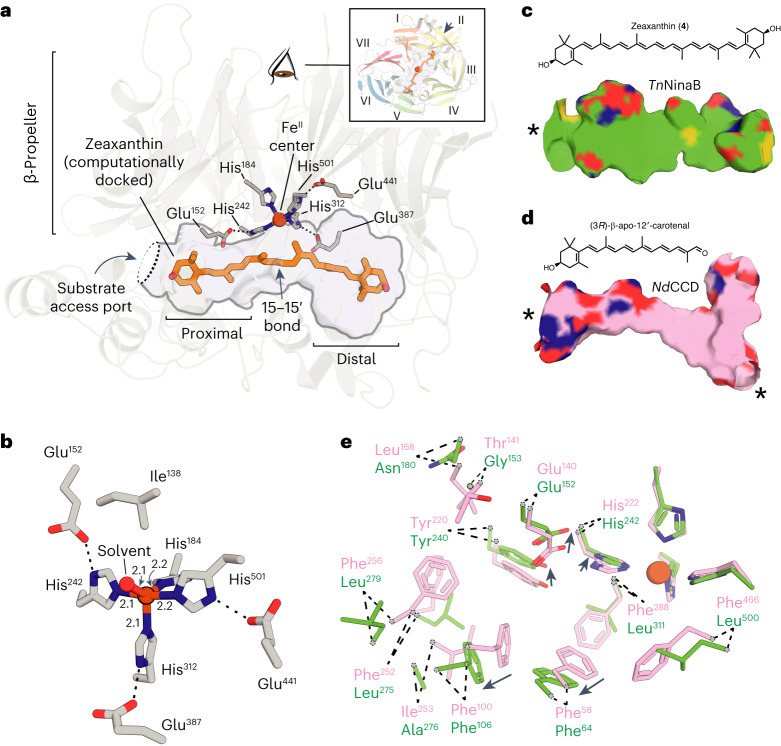
Crystal structure of *Tn*NinaB and its comparison with *Nd*CCD. **a**, Overview of the *Tn*NinaB structure emphasizing the substrate-binding cavity structure (shown in surface representation) and its relationship to the iron center and β-propeller motif. The inset shows a view down the β-propeller axis. A computationally docked zeaxanthin molecule is shown in stick representation. **b**, Details of the *Tn*NinaB iron coordination. Numbers indicate bond lengths in angstrom. **c**, *Tn*NinaB substrate-binding cavity shown below the structure of zeaxanthin. **d**, *Nd*CCD (PDB accession code 6VCH, chain C) substrate-binding cavity shown below the structure of the *Nd*CCD substrate (3*R*)-β-apo-12′-carotenal. Asterisks in **c** and **d** denote cavity entrance/exit points. **e**, Details of the structural alterations responsible for the larger proximal active site region in *Tn*NinaB (green) compared with *Nd*CCD (pink). Arrows depict shifts in conserved residues that contribute to the cavity widening. The paired sites are considered homologous based on sequence alignment.

*Tn*NinaB adopts the classical seven-bladed β-propeller CCD fold with its iron cofactor coordinated at the propeller axis (Fig. 2a). Superposition of *Tn*NinaB onto the structures of RPE65 (ref. ^25^) and an apocarotenoid-cleaving, archaeal CCD, *Nd*CCD^26^, produced an r.m.s. deviation value of ~1.9 Å for both comparisons over 514 and 455 matched Cα atoms, respectively. In NinaB, similar to other oxygenase members of the CCD superfamily, the ferrous iron cofactor activates dioxygen for reaction with a target alkene bond within the substrate. This role notably differs from the Lewis acid function played by the iron cofactor in RPE65 (refs. ^14,27^). The NinaB iron center is coordinated by four conserved His residues and contains an additional solvent molecule bound *trans* to His184 (Fig. 2b). The sixth coordination site is occluded by Ile138, which projects its C^γ2^ methyl group towards the site *trans* to His312. The iron–His bond lengths (2.1–2.2 Å) are typical for CCDs as is the distorted square pyramidal coordination geometry^28^.

**Extended Data Fig. 4:**
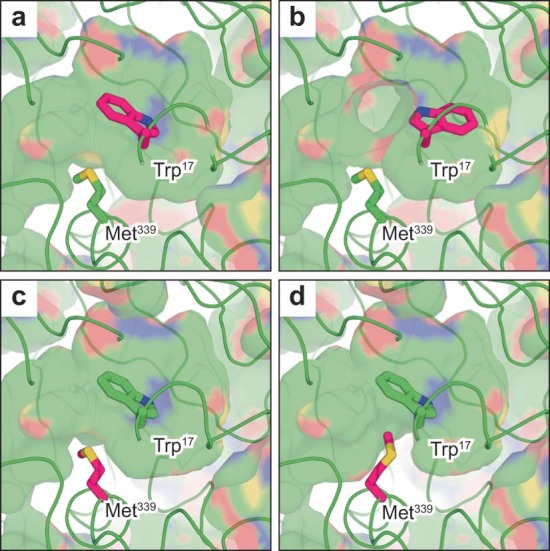
Alternative rotamers in *Tn*NinaB active site residues. **a**, Rotamer A observed for Trp17. **b**, Rotamer B observed for Trp17. **c**, Rotamer A observed for Met339. **d**, Rotamer B observed for Met339.

**Extended Data Fig. 5:**
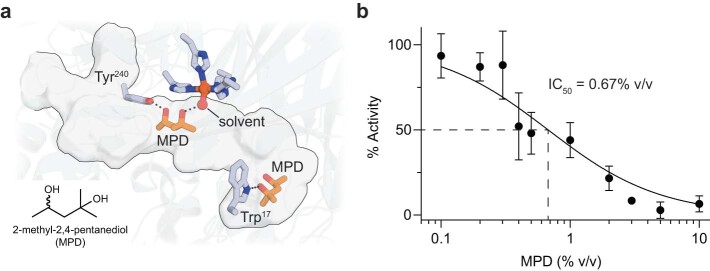
MPD (2-methyl-2,4-pentanediol) binds to the active site of *Tn*NinaB and inhibits its isomerooxygenase activity. **a**, Locations of MPD binding sites within the active site cavity. Dotted lines indicate hydrogen bonds. The inset shows the chemical structure of MPD. **b**, MPD dose-dependently inhibits *Tn*NinaB activity. The data, representing three independent experiments, were normalized as percentages of the uninhibited activity and are shown as means ± s.d. The solid line represents a non-linear fit of the following equation to the data: $\% {activity}=100/(1+\frac{\left[{MPD}\right]}{{{IC}}_{50}})$, with the best fit IC_50_ value equal to 0.67 % v/v (95% CI: 0.50–0.90 % v/v). Source data

The iron center abuts an extended pocket, ~30 Å in length, that runs along the top face of the β-propeller forming the substrate-binding cleft of the enzyme (Fig. 2a). As in other CCDs, the pocket opening is adjacent to a hydrophobic surface that enables the enzyme to interact with membranes and micelles to extract substrate. For ease of discussion, the active site can be divided into three regions: membrane proximal, central and membrane distal (Fig. 2a). The proximal region of the pocket is approximately oval in cross-section and features several residues that are highly conserved in metazoan CCDs such as RPE65 as well as *Nd*CCD. The pocket narrows slightly at the iron center and then widens again in the distal region forming a cul-de-sac structure with notable curvature. As compared with the apocarotenoid-cleaving enzyme *Nd*CCD, the proximal active site size of NinaB is much larger owing to the need to accommodate passage of a β-ionone or 3-hydroxy-β-ionone ring during substrate binding (Fig. 2c,d). This active site expansion arises from both substitutions at sites that directly line the binding pocket as well as those outside the primary active site sphere, which allow conserved residues of known functional importance to shift outward from the pocket center providing greater space for substrate entry (Fig. 2e). The active site pocket is largely apolar and is abundant in aromatic side chains, providing favorable interactions with carotenoid substrates. Other noteworthy features of the active site include the observation of multiple rotamers for Trp17 and Met339, both located in the distal active site (Extended Data Fig. 4), as well as the presence of 2-methyl-2,4-pentanediol (MPD) derived from the crystallization precipitant, which inhibits *Tn*NinaB activity toward zeaxanthin with an half-maximum inhibitory concentration (IC_50_) value of 0.67% v/v (Extended Data Fig. 5).

**Extended Data Fig. 6:**
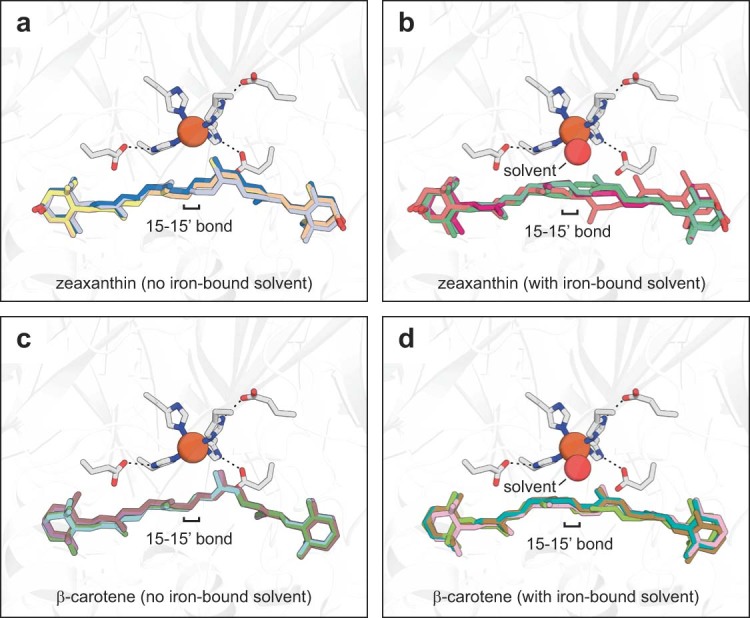
Computational docking of zeaxanthin and β-carotene into the *Tn*NinaB active site. **a**, Zeaxanthin was docked into the *Tn*NinaB active sites of chains B and H. Separate docking runs were performed with Met339 in the two different observed rotamers for chain B and with Trp17 in the two different observed rotamers for chain H, as shown in Extended Data Fig. 4. The top scoring poses for the four separate runs are shown. Docking energies for the poses ranged from −11.9 to −13.1 kcal mol^−1^. The iron-bound solvent was excluded from the model for this set of calculations. Iron is shown as a reddish-brown sphere. **b**, The docking calculation was performed as described for panel a, but with the iron-bound solvent (small red sphere) included in the model. Note the consistent placement of the 3-hydroxy-β-ionone rings as well as the appropriate positioning of the 15-15′ bond in front of the iron center for oxidative cleavage. The top scoring poses for the four separate runs are shown. Docking energies for the poses ranged from −11.8 to −12.6 kcal mol^−1^. **c**, Docking of β-carotene into the *Tn*NinaB active site using the same models described for panel a. The top scoring poses for the four separate runs are shown. Docking energies for the poses ranged from −12.7 to −13 kcal mol^−1^. **d**, The docking calculation was performed as described for panel a, but with the iron-bound solvent included in the model. Note the consistent placement of the β-ionone rings as well as the appropriate positioning of the 15-15′ in front of the iron center for oxidative cleavage. The top scoring poses for the four separate runs are shown. Docking energies for the poses ranged from −12.3 to −12.8 kcal mol^−1^.

To visualize how carotenoid substrates might interact with the NinaB active site, we computationally docked zeaxanthin and β-carotene into the substrate-binding pocket of two crystallographically independent chains (Fig. 2a and Extended Data Fig. 6). We consistently observed binding of the substrates with the scissile 15–15′ bond appropriately positioned for cleavage^26,28^. With reference to the snug mode of apocarotenoid binding to the *Nd*CCD active site, the fit of carotenoids to the NinaB active site is comparatively looser, reflecting the broader substrate-binding cleft required to accommodate passage of the β-ionone ring. The NinaB active site is similarly broader than that of RPE65, which also metabolizes an apocarotenoid derivative (all-*trans*-retinyl ester). Taken together with previous information on RPE65 and *Nd*CCD, the NinaB structure provides a detailed rationalization of the active site determinants of bicyclic carotenoid versus apocarotenoid substrate specificity within a set of structurally similar CCDs.

### NinaB membrane-binding surface and ‘PDPC(+)’ motif

**Fig. 3:**
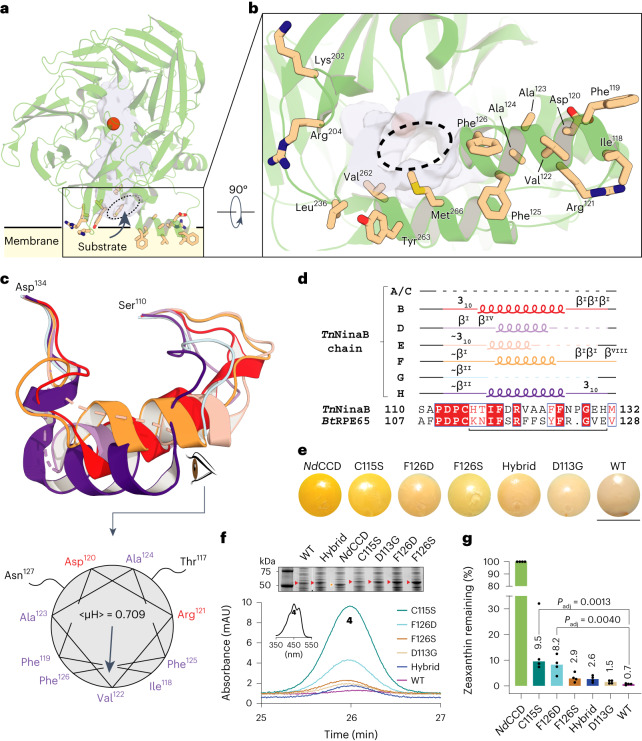
Structure and function of the *Tn*NinaB membrane-binding surface. **a**, Structure of *Tn*NinaB showing groups of hydrophobic and positively charged residues surrounding the active site entrance (dashed oval). **b**, A 90° turned-view of the membrane-binding patch. **c**, Conformational variability in the membrane-binding sequence consisting of residues 110–134. Chains B and D–H are shown in red, lavender, wheat, orange, light-blue and purple, respectively. The lower panel shows a helical wheel plot of residues 118–126 of chain H. <μH> indicates the magnitude of the first hydrophobic moment and the arrow shows its direction. **d**, Secondary structure for residues 110–132 of the different chains in the *Tn*NinaB structure. Dashed lines, loops, 3_10_ and β indicate unmodeled sequence, α-helices, 3_10_ helixes and β-turns, respectively. A tilde indicates resemblance to the indicated secondary structure. The bracket beneath the sequences demarcates the region that was substituted to generate the NinaB–RPE65 hybrid sequence. **e**, Cell pellets obtain**e**d from zeaxanthin-producing *E. coli* that co-expressed the indicated CCD enzymes or point mutants. Scale bar, 2 cm. **f**, HPLC analysis of zeaxanthin remaining in the *E. coli* cells at the end of the culture period. The chromatograms were recorded at 450 nm. The inset shows a representative absorbance spectrum. The upper Coomassie-stained SDS–PAGE gel shows the expression levels of each CCD (marked by arrows). **g**, Levels of zeaxanthin remaining at the end of the culture period expressed as a percentage relative to *Nd*CCD. The data, representing *n* = 4 independent experiments, are shown as medians (numbers above the bars) together with individual replicates (circles). The data were analyzed using the Kruskal–Wallis test which showed a significant difference among the mean group ranks (Kruskal–Wallis statistic = 19.53, *P* = 0.0015). Post hoc comparisons with WT *Tn*NinaB revealed significant mean rank differences only for the C115S and F126D mutants. Multiplicity-adjusted *P* values were calculated with Dunn’s test. Source data

*Tn*NinaB exhibits a surface near its active site entrance featuring several exposed lipophilic and cationic side chains that promote membrane/micelle interactions, allowing access to carotenoid substrates^24^ (Fig. 3a,b). Contributing to this surface are four discontinuous regions composed of residues 118–126, 202, 204, 236, 262–263 and 266. The presence of multiple copies of *Tn*NinaB in the asymmetric unit revealed that residues 118–126 and those immediately adjacent (residues 112–131) are dynamic, as reflected by their elevated atomic *B*-factors, and capable of adopting a variety of conformations (Fig. 3c and Supplementary Video 1).

**Extended Data Fig. 7:**
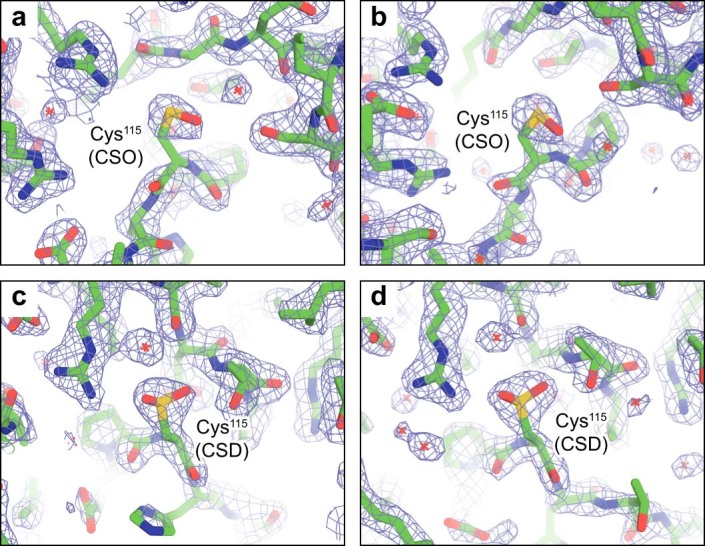
Electron density in vicinity of oxidized Cys115 residues of the *Tn*NinaB structure. **a**, Chain E showing oxidation of Cys115 to the sulfenic acid (CSO). **b**, Chain F showing oxidation of Cys115 to the sulfenic acid (CSO). **c**, Chain G showing oxidation of Cys115 to the sulfinic acid (CSD). **d**, Chain H showing oxidation of Cys115 to the sulfinic acid (CSD). Note the positively charged environment surrounding the sulfinic acids in **c** and **d**. The mesh represents sigma A-weighted 2Fo-Fc electron density contoured at the 1 r.m.s. deviation level.

This mobile region starts with a ‘PDPC(+)’ motif, (+) indicating a cationic residue, that is conserved in metazoan CCDs^29,30^ (Fig. 3d). Previous studies demonstrated that the Cys residue of this motif can be palmitoylated, which contributes to membrane affinity of the sequence^31,32^. In RPE65, it is thought that this motif initiates an extended amphipathic α-helix that plays a key role in targeting the protein to membranes^33^. In the *Tn*NinaB crystal structure, three of the protomers in the asymmetric unit (chains B, F and H) exhibited well-defined density for the entire region while three others had partially resolved sequences (chains D, E and G). We found that the ‘PDPC(+)’ motif is highly flexible and capable of adopting 3_10_ helix-, type I β-turn- and type II β-turn-like structures (Fig. 3d). The Cys115 residue exhibits density consistent with sulfenic (chains E and F) or sulfinic (chains G and H) acid modifications that likely occurred during protein purification and/or crystallization (Extended Data Fig. 7). Notably, Cys115 was the only Cys residue in the structure exhibiting oxidative modification, consistent with its accessibility and augmented chemical reactivity. Although Cys acylation was not observed in the structure, Cys115 appears to be appropriately positioned such that an attached palmitoyl group would reinforce membrane binding. Residues 118–126 form a variable-length α-helical structure with pronounced amphipathicity (Fig. 3c). Similar amphipathicity has been predicted for the corresponding sequence of RPE65 (ref. ^34^). Analysis with the Orientations of Proteins in Membranes (OPM) server^35^ indicates that residues 118–126 can penetrate the lipid bilayer core up to a depth of ~6.2 Å (Fig. 3a). The C-terminal end of the sequence (residues 127–131) is again structurally variable, adopting either a series of type I/VIII β-turns or a 3_10_-helical structure, which then joins back with a structurally invariant region of the protein (Fig. 3c,d). The overall conformation of the 112–131 segment of NinaB resembles the corresponding region of a recently determined CCD structure from *Caenorhabditis elegans*, although the α-helix observed in the *C. elegans* structure has a partially buried hydrophobic face^36^. The sequence similarity in this region between RPE65 and NinaB (Fig. 3d) suggests the conformational dynamics we observe for NinaB could serve as a model for RPE65 and the vertebrate CCD family in general.

To probe the importance of this mobile sequence for *Tn*NinaB activity, we employed an assay system in which zeaxanthin is synthesized within the inner phospholipid membranes of *E. coli* cells where it can serve as a potential substrate for the co-expressed NinaB enzyme^37,38^. Enzymatic activity can be assessed visually by a shift in the cell pellet color from orange to white (Fig. 3e) or quantitatively by HPLC analysis of the quantity of zeaxanthin remaining at the end of the culture period (Fig. 3f,g). We generated *Tn*NinaB point mutants, focusing on sites that were reported to impact function in other CCDs or that appeared likely to mediate membrane binding based on our structural data. Moreover, we replaced the entire *Tn*NinaB mobile α-helix region with the corresponding sequence from RPE65. *Nd*CCD, an apocarotenoid oxygenase, served as a negative control for these experiments. We found that Cys115, contained within the PDPC(+) motif, is highly important for activity, similar to what was observed for RPE65. Conversely, a D113G mutation within this motif, which is associated with RPE65-associated retinitis pigmentosa, had lesser impact on *Tn*NinaB activity. Mutation of Phe126 to Ser or Asp impaired activity in a manner consistent with the expected degree of perturbation to the helix amphipathicity. We found that substitution of the *Tn*NinaB mobile loop with the corresponding sequence from bovine RPE65 largely preserved NinaB catalytic activity towards zeaxanthin, suggesting that the general amphipathic character of this region rather than its specific sequence is most critical for support of catalytic activity. However, even substitutions that are expected to disrupt this amphipathic character, for example, F126D, did not abolish *Tn*NinaB activity, which indicates that other sites within the protein can also confer membrane affinity and enable substrate uptake from membranes. This conclusion is supported by the NinaB structure, which shows multiple hydrophobic/cationic regions close to the active site entrance that each likely contributes to productive membrane interactions.

### Residues mediating NinaB carotenoid isomerase activity

**Fig. 4:**
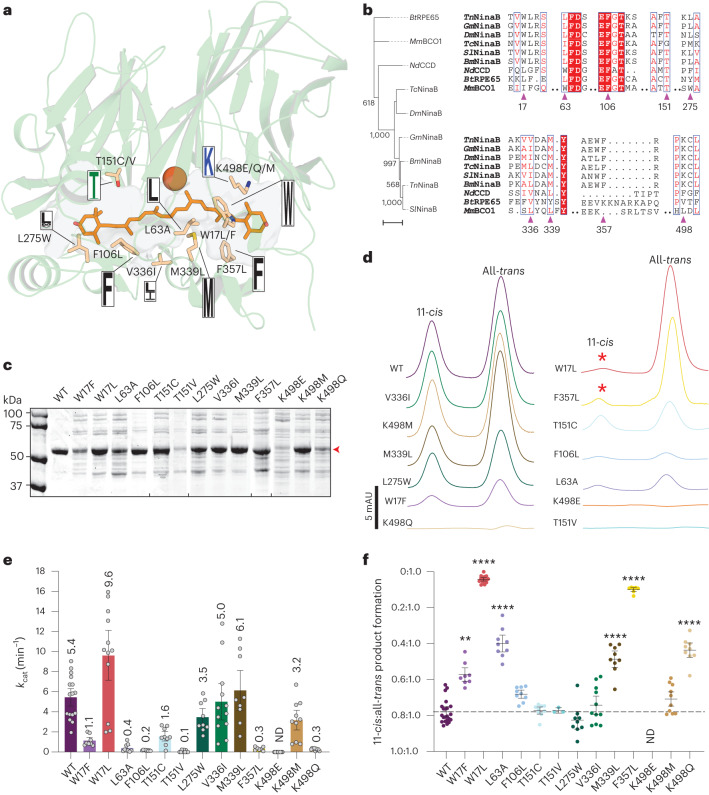
Identification of the active site region responsible for NinaB isomerase activity. **a**, Structure of *Tn*NinaB showing residues (wheat-colored sticks) selected for mutagenesis, the docked zeaxanthin molecule (orange sticks) and the iron center (sphere). Boxed letters are sequence logos showing the degree of conservation at each position for high-confidence NinaB orthologs. **b**, Maximum likelihood phylogeny (left) and amino acid sequence alignment (right) of verified isomerooxygenases together with *Nd*CCD, *Bos taurus* RPE65 and *Mus musculus* BCO1. The phylogeny was computed from aligned amino acid sequences under the LG + G + I model of evolution with four rate categories. Numbers on the tree bipartitions are bootstrap values from 1,000 pseudoreplicates. Bootstrap values > 500 are shown. The scale bar denotes an average of one expected substitution per site. Sites targeted for mutagenesis are marked with magenta arrows. **c**, SDS–PAGE analysis of purified *Tn*NinaB mutants. The red arrowhead at ~57 kDa indicates the position of *Tn*NinaB. Tick marks indicate borders of separate gels. The experiment was repeated twice with similar results. **d**, Representative 360-nm absorbance profiles showing the levels of 11-*cis*- and all-*trans*-retinoids formed from zeaxanthin by the *Tn*NinaB mutants. Asterisks highlight two mutations (W17L and F357L) that largely eliminated formation of 11-*cis*-retinoid with retention of all-*trans*-retinoid formation. **e**, Turnover numbers for WT and point-mutated *Tn*NinaB. Data are presented as means ± s.e.m. together with values for each replicate (gray circles). Each independent experiment (*n*) included three technical replicates. WT (*n* = 6); W17L, V336I and K498M (*n* = 4); other mutants (*n* = 3). **f**, Quantification of 11-*cis*:all-*trans*-retinoid production ratios for the various mutants. Data are plotted as $1-\frac{11{\hbox{-}}{cis}}{{\rm{all}}{\hbox{-}}{trans}}$ and presented as means ± s.e.m. together with values for each replicate (colored circles). For clarity, the *y* axis is labeled with the corresponding 11-*cis*:all-*trans* ratios. The data were analyzed by one-way analysis of variance (ANOVA) (*F*_(12,59)_ = 50, *P* < 0.0001) followed by Dunnett’s post hoc test (****P_adj_ < 0.0001, **P_adj_ = 0.0025). ND, not detectable/determined. Each independent experiment (*n*) included three technical replicates. WT (*n* = 5); W17L, V336I and K498M (*n* = 4); T151V (*n* = 1); other mutants (*n* = 3). Source data

With the *Tn*NinaB structure crystallographically defined, we next sought to elucidate the specific region of its expansive substrate-binding cavity that confers isomerase activity. For this, we employed our model of the enzyme–zeaxanthin complex to select amino acid residues directly lining the active site cavity that are plausibly positioned to contribute to isomerization activity (Fig. 4a). Most of the residues we selected are highly conserved in functionally verified and other high-confidence NinaB proteins (Fig. 4a,b). We also probed NinaB active site residues that are identical to those residues in RPE65 known to be influential in its isomerization activity (that is, Phe106, Thr151)^14^. Using these criteria, a total of nine sites, located throughout the active site cavity, were selected for mutagenesis. Each mutant was expressed in an otherwise native form, purified as described for the wild-type (WT) protein and enzymatically characterized.

**Fig. 5:**
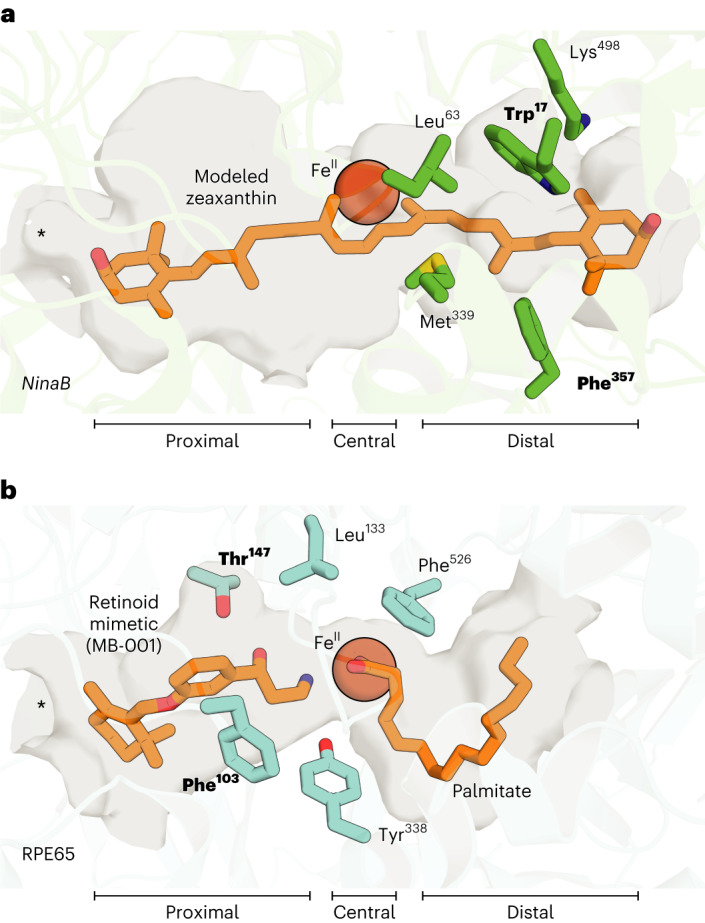
Differing loci of isomerization activity in the NinaB and RPE65 active sites. **a**, Active site of *Tn*NinaB with the docked zeaxanthin substrate showing the location of residues important for isomerase activity within the distal active site. Trp17 and Phe357 (bolded) are especially important for NinaB isomerization catalysis. **b**, Active site of RPE65 with a bound retinoid mimetic (MB-001) and palmitate (PDB accession code 4RSE) together with residues that govern retinoid product isomer specificity. Note that most of these residues localize to the proximal active site where retinoid binds during catalysis. Phe103 and Thr147 (bolded) are shown to be especially important for RPE65 isomerase activity. Asterisks are placed over the active site entrances.

**Extended Data Fig. 8:**
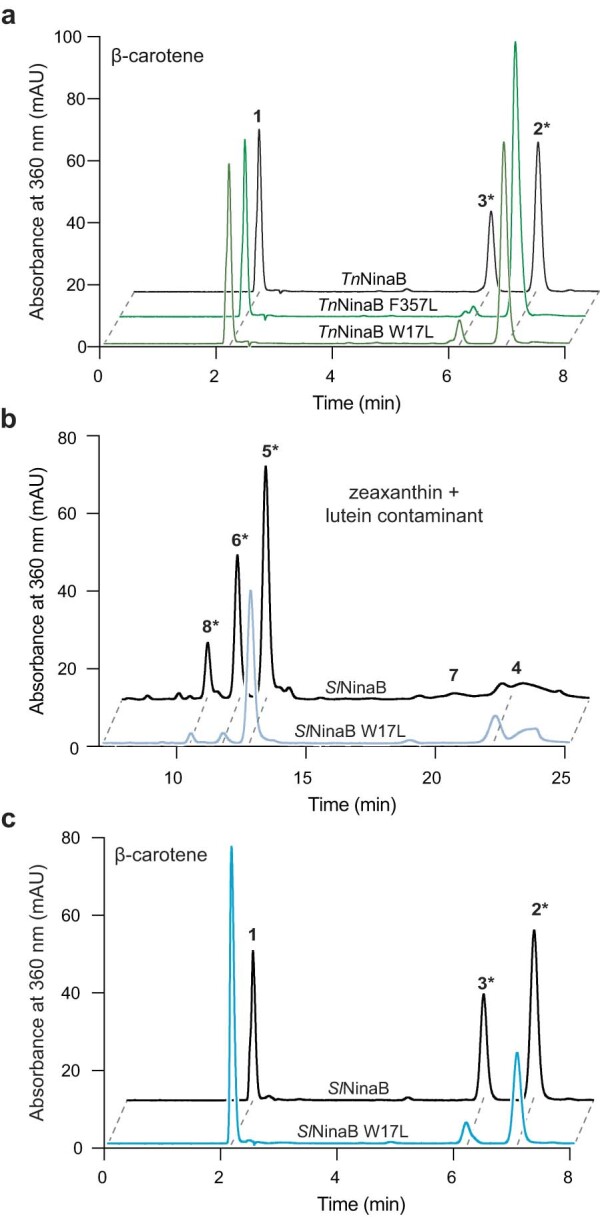
Activity of *Tn*NinaB and *Sl*NinaB wild-type and point mutant proteins towards β-carotene and zeaxanthin. **a**, *Tn*NinaB W17L and F357L point mutations nearly extinguished formation of 11-*cis*-RAL (**3**) from β-carotene (**1**) despite preserved carotenoid oxygenase activity, as demonstrated by formation of all-*trans*-RAL (**2**). **b**, The W17L substitution in *Sl*NinaB drastically reduces its ability to form (3*R*)-3-hydroxy-11-*cis*-RAL (**6**) from zeaxanthin (**4**) despite preserved oxygenase activity as demonstrated by the formation of (3*R*)-3-hydroxy-all-*trans*-RAL (**5**). (3*R*,6*R*)-3-hydroxy-all-*trans*-α-RAL (**8**) was formed from contaminating lutein present in the substrate stock. **c**, The W17L substitution in *Sl*NinaB drastically reduces its ability to form 11-*cis*-RAL (**3**) from β-carotene (**1**) despite preserved oxygenase activity as demonstrated by the formation of all-*trans*-RAL (**2**). Retinoids were converted to oxime derivatives prior to HPLC analysis, which is denoted by asterisks next to compound numbers. The data are representative of three independent experiments. Source data

Point mutations typically reduced *Tn*NinaB expression levels to variable degrees, although most of the mutants could be obtained in quantities sufficient for enzymatic testing (Fig. 4c). The activity of mutant *Tn*NinaB enzymes towards zeaxanthin was characterized both in terms of their overall activity level, measured by the total production of 3-hydroxy-RALs, as well as their differential production of the all-*trans* and 11-*cis* isomers (Fig. 4d). The WT *Tn*NinaB enzyme exhibited a turnover number (*k*_cat_) towards zeaxanthin of ~5 min^−1^ (Fig. 4e), as compared with the *k*_cat_ value of 0.5 min^−1^ estimated for *Gm*NinaB towards β-carotene, and produced 11-*cis* and all-*trans* isomers at a ratio of 0.78:1 (Fig. 4f). Active site mutations reduced *Tn*NinaB catalytic activity in many cases although quantifiable activity was obtained for all except the K498E mutant, which was among the poorest expressed mutants in our screen. Although most of the mutants displayed 11-*cis*:all-*trans*-RAL production ratios comparable to the WT enzyme, we identified five sites at which mutations significantly altered this ratio—Trp17, Leu63, Met339, Phe357 and Lys498. Among these, the W17L and F357L mutations produced the most dramatic activity change with near complete abolishment of 11-*cis* isomer formation. Notably, the W17L mutant oxygenase activity was comparable to WT level, indicating Trp17 plays a selective role in the isomerase activity of NinaB. Comparable results were obtained when β-carotene was used as the substrate (Extended Data Fig. 8a), except that cleavage activity was substantially higher for the F357L mutant with this substrate. To probe the generality of the selective role of Trp17 in NinaB isomerase activity, we introduced a W17L mutation into *Sl*NinaB and tested its activity towards zeaxanthin and β-carotene. In both cases, 11-*cis* isomer production was nearly abolished despite robust oxygenase activity as evidenced by WT-level formation of the all-*trans* isomer product (Extended Data Fig. 8b,c). Mapping Trp17, Phe357 and the other residues impacting 11-*cis* formation within the *Tn*NinaB active site revealed their clustering within the distal region of the active site pocket (Fig. 5a).

Notably, substitutions in residues homologous to those involved in RPE65 isomerization activity, Thr151 (Thr147 in RPE65) and Phe106 (Phe103 in RPE65), which are found in the proximal active site (Fig. 4a), dramatically reduced oxygenase activity of *Tn*NinaB but did not alter the distribution of its product isomers (Fig. 4e,f). Similarly, none of the other proximal active site substitutions we studied (L275W, V336I) altered the product isomer specificity of the enzyme. These findings demonstrate that isomerization activities in NinaB and RPE65 are mediated by distinct active site regions (Fig. 5a,b).

## Discussion

The structure of NinaB and activity data that we describe in this work: (1) provide an understanding of the active site adaptations that determine apocarotenoid versus carotenoid cleavage activity in CCDs; (2) provide a dynamic picture of a functionally critical, but structurally enigmatic, membrane-binding region of metazoan CCD proteins; and (3) identify the specific residues responsible for NinaB *trans–cis* isomerase activity, providing compelling evidence for convergent evolution of the isomerase activity in CCD-dependent visual chromophore synthesis.

The question of which CCD active site features dictate bicyclic carotenoid versus apocarotenoid cleavage specificity was left unanswered following structure determination of the *Synechocystis* apocarotenoid oxygenase^39^. Later studies on *Nd*CCD, in which a genuine enzyme-apocarotenoid complex was resolved, showed that apocarotenoid substrate specificity as well as cleavage regioselectivity are enforced by multiple residues within the proximal active site region^26^. The phylogenetic and structural relatedness of NinaB to *Nd*CCD provides a unique opportunity to delineate the specific active site changes allowing for bicyclic carotenoid cleavage. Specifically, the NinaB proximal active site is substantially larger in volume compared with that of *Nd*CCD, which allows the β-ionone moiety to traverse the active site cavity to reach its catalytically relevant binding position. The structural changes giving rise to this expansion are numerous but involve both direct active site amino acid substitutions as well as substitutions outside the primary active site that enable repositioning of conserved residues. Two such conserved sites are Phe106 (Phe103 in RPE65) and Thr151 (Thr147 in RPE65). The repositioning of these sites in NinaB, as necessitated to accommodate a larger substrate, appears to render them unable to contribute to isomerization catalysis since they cannot achieve the necessary close apposition observed in the RPE65 structure. Similar modifications in both primary and secondary residues underlying changes in substrate specificity have been observed in enzymes such as sesquiterpene synthases^40,41^.

**Extended Data Fig. 9:**
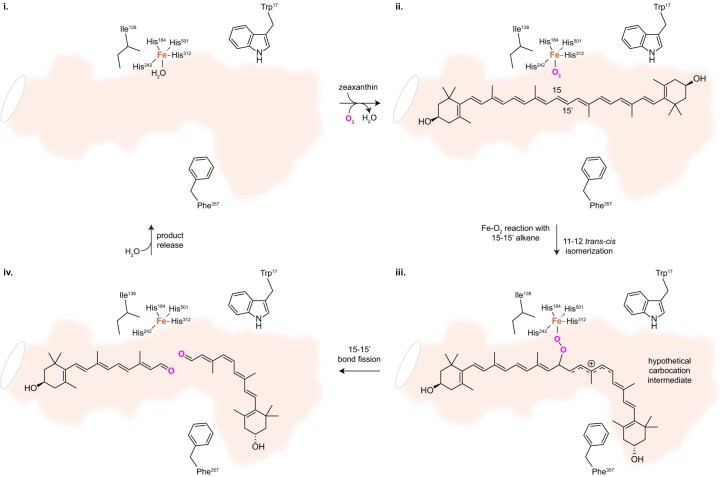
Hypothetical mechanism for the carotenoid isomerooxygenase reaction catalyzed by NinaB. ***i****.* The resting state of the enzyme features a five-coordinate Fe(II) center with a solvent molecule (likely water) bound at the exchangeable site. This resting state structure is based on the *Tn*NinaB crystal structure data as well as published data on other CCDs. ***ii****.* Substrate diffuses into the active site from the membrane/micelle and binds with the scissile 15-15′ bond positioned next to the iron center, consistent with our ligand docking calculations. Exchange of solvent for dioxygen (O_2_) may occur randomly or may be facilitated by binding of the carotenoid substrate. ***iii****.* The iron-oxy complex reacts with the substrate to form an intermediate, such as the resonance-stabilized carbocation intermediate shown here. Such intermediates have been predicted to form based on computational studies, but none have been experimentally observed to date. The intermediate provides the lowered bond order needed for the *trans-cis* isomerization reaction to occur. As shown in the present study, Trp17 and Phe357 (*Tn*NinaB numbering) play crucial roles in the isomerization reaction. ***iv****.* Continued reaction of dioxygen with the scissile bond results in formation of the two aldehyde products and restoration of the polyene bond order. The energy released from the oxygenation reaction is sufficient to offset the endergonic *trans-cis* isomerization, making the overall reaction thermodynamically favorable. The hydrophobic aldehyde products are released from the enzyme through the same opening as substrate entry, back into the membrane/micelle.

In stark contrast to the active site fortifications that ensure proper substrate specificity and cleavage regioselectivity, we observed that single amino acid substitutions at five different positions in the NinaB enzyme could dramatically alter the isomeric products of catalysis. The five residues all localize to the distal active site, suggesting that they may work in concert to ensure an active site environment conducive to the isomerization process. Among these, a W17L substitution produced a strikingly selective effect on the isomerization activity of the enzyme with complete retention of canonical oxidative cleavage activity. The W17L substitution nearly abolished 11-*cis* isomer formation from both carotene and xanthophyll substrates, whereas an W17F substitution possessed an all-*trans*/11-*cis* production ratio only slightly elevated relative to the WT enzyme. Notably, an examination of high-confidence NinaB ortholog sequences revealed that position 17 is conserved in the Trp state with a few enzymes exhibiting Phe in place of the Trp. Other metazoan CCDs do not possess a Trp/Phe residue at this position, suggesting that this single amino acid change may have been a major factor in the development of isomerase activity in the NinaB enzyme lineage. An F357L substitution produced a similarly striking reduction in 11-*cis* isomer formation although its effect on oxygenase activity depended on the specific substrate used in the assay. Trp17 and Phe357 are located across from each other in the distal active site and are plausibly positioned to facilitate isomerization through steric effects, π–π stacking interactions and/or quadrupole stabilization of reaction intermediates including cations or radical cations^42–44^, which have been invoked to explain how bond order lowering occurs during carotenoid/retinoid isomerization (Extended Data Fig. 9)^23,24^. It is noteworthy that both type 1 and type 2 opsin proteins feature Trp and other aromatic residues in proximity to the photoisomerization-labile double bond of RAL^3^. Likewise, squalene cyclase catalyzes a complex, multisite double bond isomerization reaction using a Trp-rich active site^45^. Our finding that Phe but not Leu can largely functionally substitute for Trp at position 17 further indicates that aromaticity, cation stabilization capacity and potentially side-chain volume are important attributes that support isomerization activity. Similar, but nonhomologous, retinoid–aromatic interactions are known to contribute to RPE65 retinoid isomerase activity^13,14^. Future mechanistic studies will be required to elucidate the specific physicochemical contributions of these residues to the isomerization reaction.

Our data also provide insights into the structure and dynamics of a conserved mobile sequence beginning with a ‘PDPC(+)’ motif that plays a key role in metazoan CCD membrane binding and enzymatic function. The importance of this sequence was proposed in a paper describing the cloning of RPE65, where it was hypothesized to form a membrane-interacting amphipathic α-helix (ref. ^34^). Recent biochemical and computational studies support this proposal^33^. Additionally, the well-documented susceptibility of the Cys residue within the ‘PDPC(+)’ motif to palmitoylation is believed to further promote membrane binding of the sequence^25,31,32^. However, the structure of this sequence in RPE65 was never crystallographically resolved owing to dynamic disorder^25,27^. Consequently, the well-resolved structure of the corresponding region of NinaB substantially contributes to our understanding of this membrane-binding element. The closely packed nature of the *Tn*NinaB crystals allowed six distinct conformations of the sequence to be trapped, three of which were fully resolved. Consistent with previous proposals, we find that a portion of the sequence (residues 118–126) adopts an α-helical structure with pronounced amphipathic character allowing the helix to penetrate the lipid bilayer. However, we find that adjacent sequence regions are more variable in structure and appear to act as hinges to allow the α-helical segment to move as a rigid body. In two of the subunits, the sequence was sufficiently disordered such that it was untraceable. Thus, similar to the situation for RPE65, we find that the sequence overall is highly flexible and is locked into a specific conformation only when it is externally constrained. It has been repeatedly documented that dynamic regions of protein structures are often functionally critical^46^. In the case of NinaB and other metazoan CCDs, it is possible that the dynamic membrane-binding region plays a role in recruiting substrates from membranes/micelles and funneling them into the active site cavity. Another nonmutually exclusive possibility is a role for this region in sealing the active site once substrate is bound, as frequently observed for other enzymes.

In conclusion, this study provides detailed information on an unusual type of dual catalytic enzyme that plays a crucial role in insect vision. The distinct loci of isomerization activity observed in NinaB and RPE65 provide an interesting example of convergent evolution using differing regions of a homologous active site. This information, along with the known lack of *trans–cis* isomerase activity in vertebrate beta-carotene oxygenase (BCO) enzymes, suggests that the last common ancestor of these enzymes was not an 11-*cis*-RAL-generating enzyme. We anticipate that this information will prove useful in ongoing efforts to understand the origins of the catalytic machinery necessary to support function of visual pigments in dim-light environments^47–50^. Convergent evolution has been documented in several aspects of visual systems and RAL-based light-detection in general^51–54^. In the case of CCD-dependent visual chromophore production, this work provides an example of functionally convergent evolution within a protein superfamily that arose through distinct active site adaptations^55^. Similar evolutionary scenarios have been proposed for the globin^56^ and thioredoxin/glutaredoxin^57^ superfamilies. Our identification of residues critically involved in the carotenoid isomerization process opens the door to detailed structure–function studies of light-independent alkene isomerization and provides a means to further probe the biological importance of carotenoid isomerooxygenase activity in insects.

## Methods

### Bioinformatics

Arthropod homologs of *G. mellonella* NinaB (*Gm*NinaB, Supplementary Table 1) were identified with BlastP by searching the Non-Redundant Protein Sequence Database. Sequences with variable identity (31–81%) to *Gm*NinaB, but with conservation of core catalytic residues (for example, the metal-binding 4-His cluster), were selected for expression studies (Supplementary Table 1). For sequence logo construction, the *T. ni* NinaB (*Tn*NinaB, Supplementary Table 1) amino acid sequence was used as a query sequence in BlastP to identify high-confidence insect NinaB orthologs. Unique sequences (that is, for organisms with multiple isoforms, only a single sequence was used) with greater than 60% identity^58^ to *Tn*NinaB and sequence lengths (*L*) of 500 ≤ *L* ≤ 550 residues were included in the analysis together with sequences for *Tribolium castaneum* and *D. melanogaster* NinaBs. The sequences were aligned with MUSCLE using default parameters^59^ and alignment gaps removed with Gblocks. The sequence logo was generated using the WebLogo server^60,61^. Phylogeny inference was carried out with PhyML^62^.

### Reagents and materials

Except as noted below, chemical reagents were purchased from Sigma-Aldrich, Fisher Chemical or USB Biochemicals in the highest purity form available. Ultrapure water (resistivity = 18.2 mΩ × cm) was used to prepare all aqueous solutions. All HPLC procedures and carotenoid/retinoid handling were performed under dim-red light. A preparative silica column (Luna 10 μm Silica, 250 × 21, Phenomenex) was used for large-scale carotenoid and retinoid purification methods. Zeaxanthin (Toronto Research Chemicals, Z275000) was used as supplied or purified by normal phase HPLC (mobile phase: 30:70, hexanes:ethyl acetate) before use. A minor lutein contaminant in the commercial zeaxanthin was separated by HPLC and collected for later use. β-Carotene (Sigma-Aldrich, C4582) was purified before use by normal phase HPLC (mobile phase: 90:10, hexanes:ethyl acetate).

*cis*-RAL oxime standards were produced according to published methods with some modifications^63^. Briefly, 50 mg of all-*trans*-RAL (Toronto Research Chemicals, R24000) was dissolved in 2.5 ml of acetonitrile and illuminated with an 85-W mercury light bulb (emission peak at 395 nm) at maximum intensity at 10 cm for 3 h at 4 °C. The resultant 9-*cis*-, 11-*cis*- and 13-*cis*-RAL products were purified by normal phase HPLC (mobile phase: 9:1, hexanes:ethyl acetate). Fractions containing individual RAL isomers were dried in vacuo, dissolved in 1:1 methyl *tert*-butyl ether (MTBE)/petroleum ether and then repurified by preparative HPLC to verify the isomer composition. The sample was dried under nitrogen gas at room temperature and the RAL redissolved in 90% v/v hexanes, 9.9% v/v ethanol and 0.1% v/v triethylamine. The RAL concentration was determined spectrophotometrically (Lambda Bio+, Perkin-Elmer) in ethanol using a quartz cuvette and published extinction coefficients^64^. Aliquots were dried under nitrogen, redissolved in dimethylformamide (DMF) and then subjected to serial dilution in DMF. RAL oximes were formed by the addition of 90 μl of 1 M hydroxylamine, pH 8, and 100 μl of MeOH to 10 μl of the purified RAL standards dissolved in DMF. After a 5-min incubation at room temperature, 200 μl of brine was added to the reaction followed by 400 μl of hexanes. Following vigorous shaking the mixture was centrifuged at 16,000*g* for 3 min and the organic hexanes layer containing a known mass of RAL oxime was collected and directly analyzed by HPLC on an analytical silica column (Zorbax Sil 5 μm, 4.6 × 250 mm, Agilent). Chromatograms and spectra were analyzed using ChemStation (Agilent). A standard curve relating the known amount of RAL used to form the RAL oxime standards to the *syn*-RAL oxime peak area was then generated.

### Molecular biology

Expression plasmids were purchased from Genscript. Sequences encoding proteins of interest were codon-optimized for an *E. coli* expression system and subcloned in the pET3a expression vector. Site-directed mutagenesis was carried out using the Q5 Site-Directed Mutagenesis Kit (New England Biolabs, E0554S) according to the manufacturer instructions. All parental and mutagenized plasmids were verified by Sanger sequencing.

### Protein expression

NinaB protein expression was carried out according to a published protocol^65^. Final bacterial pellets were suspended in 10 mM HEPES-NaOH, pH 7, and stored at −80 °C. The expression of some NinaB homologs was also tested in T7 express *E. coli* competent cells (New England Biolabs, C2566H) that were co-transformed with the chaperone overexpression plasmid pG-KJE8 (Takara Bio, 3340).

### NinaB purification

NinaB-expressing *E. coli* were lysed by two passes through a French pressure cell. Triton X-100 was added to the lysate to a final concentration of 0.1% v/v, and the mixture was incubated on ice for 20 min. The lysate was centrifuged at >100,000*g* for 1 h and the resulting supernatant collected. Purification procedures were conducted at 4 °C using an NGC chromatography system (Bio-Rad). The supernatant was loaded onto a HiTrap Q HP 5-ml anion-exchange column (GE Healthcare) equilibrated with 10 mM HEPES-NaOH, pH 7, and 0.05% v/v Triton X-100 (buffer A). The column was then washed with 3 column volumes of buffer A. Bound NinaB was eluted by a linear increase from 0% to 100% of buffer B containing 10 mM HEPES-NaOH, pH 7, 0.05% v/v Triton X-100 and 500 mM NaCl. Fractions containing NinaB were identified by SDS–PAGE, pooled together and concentrated in a 50-kDa molecular weight cut-off (MWCO) Amicon (Millipore). The sample was then purified by gel filtration chromatography on a 120-ml Superdex 200 column (GE Healthcare) that was pre-equilibrated with a buffer consisting of 10 mM HEPES-NaOH, pH 7, 0.05% v/v Triton X-100 and 100 mM NaCl. NinaB fractions were screened and pooled together as previously described. Fractions were diluted in ice-cold buffer A to a volume of 50 ml. The diluted sample was loaded onto a 1-ml MonoQ column (GE Healthcare) equilibrated in buffer A. NinaB was eluted with a linear gradient up to 50% of a buffer containing 10 mM HEPES-NaOH, pH 7, 0.05% v/v Triton X-100 and 1 M NaCl. Fractions containing purified NinaB (≥90% pure as judged by SDS–PAGE analysis) were pooled and concentrated to 80–100 mg ml^−1^ based on a Bradford assay (Bio-Rad, 5000006) using BSA as a standard, flash-frozen and stored at −80 °C. NinaB mutants were purified in a similar fashion except that the MonoQ chromatography step was omitted in some cases.

### Activity assays using β-carotene substrate

NinaB activity assays employing β-carotene as a substrate were performed under a dim-red safety light according to published methods with the following modifications^21,23^. First, 2 nmol purified β-carotene in 100% ethanol was mixed with 3% w/v octylthioglucoside (Anatrace, O314) and the mixture was dried in vacuo without heating. The dried material was reconstituted in 200 μl of 10 mM HEPES-NaOH, pH 7, containing 1 mM Tris-carboxyethylphosphene. The reaction was initiated by the addition of 20–25 μg of purified NinaB and then placed in a shaker-incubator operating at 300 r.p.m. and 37 °C for 1 h. Then 100 μl of 1 M hydroxylamine, pH 8, was added to the reaction to convert aldehyde products into oximes. After a 5-min incubation at room temperature, 400 μl of acetone, 400 μl of diethyl ether and 100 μl of petroleum ether were added to the mixture and the retinoid/carotenoid components extracted by vigorous shaking. Following centrifugation at 16,000*g* for 3 min, the organic layer was collected and dried in vacuo. Dried material was redissolved in 300 μl of 90:10 hexanes:ethyl acetate and then analyzed on a diode-array detector-equipped HPLC system (1260 Infinity II Series, Agilent) using a Zorbax silica column (4.6 μm, 250 mm) and a 90:10 hexanes:ethyl acetate mobile phase flowing at 1.4 ml min^−1^.

### Activity assays using xanthophyll substrates

NinaB activity assays employing xanthophylls as substrates were performed under dim-red safety light according to published methods with the following modifications^21,23^. Chromatographic separation was achieved with an isocratic mobile phase of 70:30 hexanes:ethyl acetate at a flow rate of 1.4 ml min^−1^. Test substrates zeaxanthin and lutein were purified as mentioned above. The enzymatic activity of NinaB proteins towards zeaxanthin and lutein was performed as previously described^21^ with the following modifications. Aliquots of NinaB proteins suspended in 10 mM HEPES pH 7 were removed from a −80 °C freezer and thawed on ice. Reactions were performed in 1.5-ml Eppendorf tubes with a final volume of 250 μl. A 250-μl solution consisting of 20 mM HEPES-NaOH, pH 7, 20 μg of purified NinaB protein and 5 mM lauryl maltose neopentyl glycol (Anatrace, NG310) was first prepared. The reaction was initiated by the addition of 10 nmol of zeaxanthin or lutein dissolved in 100% ethanol. The reaction mixture was placed in a shaker-incubator operating at 300 r.p.m. and 25 °C for 30 min. Then 100 μl of 1 M hydroxylamine, pH 8, was added to the reaction to convert aldehyde products into oximes. After a 5-min incubation at room temperature, 400 μl of acetone, 400 μl of diethyl ether and 100 μl of petroleum ether were added to the mixture and the retinoid/carotenoid components extracted by vigorous shaking. Following centrifugation at 16,000*g* for 3 min, the organic layer was collected and dried in vacuo. Dried material was redissolved in 300 μl of 70:30 hexanes:ethyl acetate and analyzed on a diode-array detector-equipped HPLC system (1260 Infinity II Series, Agilent) using a Zorbax silica column (4.6 μm, 250 mm; Agilent) and a 70:30 hexanes:ethyl acetate mobile phase flowing at 1.4 ml min^−1^.

### Analysis of NinaB-catalyzed reaction products

Absorbance peaks for RALs were integrated within ChemStation software (Agilent) and converted to absolute amounts based on standard curves generated using authentic RAL standards. For the purposes of data plotting, the ratio of the masses of 11-*cis* and all-*trans* retinoid isomers formed was calculated as $1-\frac{11{\hbox{-}}{cis}}{{\rm{all}}{\hbox{-}}{trans}},$ where a value of 0 indicates a 50:50 mixture of 11-*cis* and all-*trans* isomers and a value of 1 indicates exclusive production of the all-*trans* isomer.

### NinaB assays in zeaxanthin-accumulating *E. coli*

A plasmid carrying the genes for zeaxanthin biosynthesis from *Erwinia herbicola*^66^ as well as a chloramphenicol-resistance cassette was transformed into T7 Express *E. coli* competent cells (New England Biolabs, C2566H). Positive clones were selected on agar culture plates containing chloramphenicol at a concentration of 34 μg ml^−1^. One positive clone was inoculated into 3 ml of LB medium containing 34 μg ml^−1^ chloramphenicol, which was placed in a shaker-incubator and grown for 12 h at 37 °C. This starter culture was then transferred into 250 ml of LB medium containing 34 μg ml^−1^ chloramphenicol, which was grown in a darkened shaker-incubator at 37 °C until an optical density (OD)_600 nm_ of 0.6 was reached. The cells were then pelleted by centrifugation at 4 °C, resuspended into 10 ml of ice-cold 100 mM CaCl_2_ until homogenous and incubated on ice for 10 min, a process that was carried out a total of three times. After the last wash, the pellet was resuspended in 3 ml of 100 mM CaCl_2_ and 15% v/v glycerol. The resulting zeaxanthin-producing competent cells were divided into 50-μl aliquots, flash-frozen in liquid nitrogen and stored at −80 °C.

CCD expression plasmids were introduced into the zeaxanthin-producing *E. coli* by heat-shocking at 42 °C for 30 s, and positive clones were selected on agar culture plates containing 100 μg ml^−1^ ampicillin and 34 μg ml^−1^ chloramphenicol. Positive clones were cultured in LB medium containing the same concentrations of ampicillin and chloramphenicol at 37 °C in a darkened shaker-incubator until the culture reached an OD_600 nm_ of 0.6. At this time, the incubator temperature was adjusted to 27 °C and the culture was allowed to continue growing for 19 h. CCD expression occurred constitutively due to the leaky behavior of the T7 promoter in this system. The levels of zeaxanthin present in the cultures at the end of the incubation period were visually assessed by collecting the cells by centrifugation and observing the color of the pellet. To obtain a quantitative measurement of the amount of zeaxanthin remaining after the incubation period, cells were pelleted from 4 ml of the cultures, resuspended in 20 mM HEPES-NaOH, pH 7, to a final volume of 300 µl, and then lysed by sonication. Next, 300 µl of brine, 400 µl of acetone, 400 µl of diethyl ether and 200 µl of petroleum ether were added to the lysate and the carotenoid components were extracted by vigorous shaking. The sample was centrifuged at 16,000*g* for 5 min to separate the aqueous and organic layers, and the upper organic layer was collected and dried in vacuo. Dried material was redissolved in 300 µl of 70:30 hexanes:ethyl acetate and then separated on a Zorbax silica column (4.6 µm, 250 mm; Agilent) with a mobile phase of 70:30 hexanes:ethyl acetate flowing at 1.4 ml min^−1^. Chromatography was performed on a diode-array detector-equipped HPLC system (1260 Infinity II Series, Agilent).

### *Tn*NinaB crystallization

*Tn*NinaB crystallization was performed by the sitting-drop, vapor-diffusion method by mixing 1.3 μl of purified protein at a concentration of 30 mg ml^−1^ in 10 mM HEPES-NaOH, pH 7, 100 mM NaCl and 0.05% v/v Triton X-100 with 1.3 μl of various commercially available crystallization screens. Plate-like crystals with approximate dimensions of 20 × 150 × 150 μm^3^ were observed after 2 d of incubation at room temperature in condition no. 1 of the Wizard Cryo II crystallization screen (Rigaku, 1009537), which consisted of 100 mM sodium cacodylate, pH 6.5, 40% v/v MPD and 5% w/v PEG 8000. The crystals were collected 4 d later using dual-thickness MiTeGen loops and flash-cooled in liquid nitrogen. The crystals were stored in liquid nitrogen vapor before X-ray data collection.

### X-ray data collection, structure solution and refinement

*Tn*NinaB X-ray diffraction data were collected at the Northeastern Collaborative Access Team (NE-CAT) beamlines at the Advanced Photon Source (APS) and at beamlines 12-1 and 12-2 at the Stanford Synchrotron Light Source (SSRL). The diffraction data were processed and analyzed with XDS^67^ and phenix.xtriage^68^. Pseudotranslational symmetry was identified based on a strong peak in the Patterson function (53% of the origin peak). *Tn*NinaB crystals diffracted to a nominal resolution of 1.95 Å and belonged to space group *C*2. Data collection statistics are shown in Supplementary Table 2. Structure solution was carried out in MrBUMP^69^ within the CCP4 online server. An AlphaFold2 model of *Gm*NinaB (AF-A8Y9I2-F1) was used for molecular replacement using the program Phaser^70^. Eight copies of the protein were found in the asymmetric unit. The initial solution was used for automated model building within the program ARP/wARP^71^. The resulting model was then used as a starting point for manual model building in Coot^72^ alternating with reciprocal space refinement in REFMAC5 (ref. ^73^). A single aquo ligand was placed at the exchangeable position of the iron center. After refinement, the aquo ligand and iron *B*-factors were similar in magnitude and no difference density was present at the exchangeable position of the iron center, thus ruling out the presence of alternative ligands (for example, dioxygen or a halide). Ordered MPD molecules were also observed in the NinaB active site and on its surface. A few relatively strong (5–7 r.m.s. deviation) difference map peaks within the active site could not be convincingly assigned to known components of the crystal mother liquor. The geometric integrity and electron density fit of the model were accessed with the Molprobity^74^ and wwPDB^75^ validation servers. The final model was characterized by working and free crystallographic *R*-factors (*R*_work_ and *R*_free_) values of 21.8% and 24.7%, respectively (Supplementary Table 2). The somewhat elevated *R* values can be attributed to the pseudotranslational symmetry in the data, which produces bimodal intensity statistics. The Ramachandran plot featured 96.9% of residues in favored regions with no outliers. The Molprobity global score and all-atom clash score were 1.02 (100th percentile) and 1.21 (100th percentile), respectively. Structural figures were generated using PyMOL (Schrödinger).

### In silico substrate docking

Docking calculations were carried out using the AutoDock suite of programs^76,77^. Chains B and H from the *Tn*NinaB structure were used for docking of zeaxanthin and β-carotene. The two alternative conformations for Met339 (chain B) and Trp17 (chain D) were considered in the docking calculations. Additionally, we performed docking studies with or without the iron-bound solvent included in each of the models. The *Tn*NinaB, zeaxanthin and β-carotene coordinates were prepared within AutoDockTools and the docking calculations were carried out using AutoDock Vina. Polar hydrogens were included in the models. The search area included a 36 × 22 × 32 Å^3^ box centered in front of the iron center and encompassing the entire active site cavity. The exhaustiveness parameter was set to 500. All single bonds within the polyene were treated as rotatable in the docking calculations. The top scoring poses for each of the receptor–ligand pairs are shown in Extended Data Fig. 6.

### Statistical analyses

Enzymatic activity data are presented as means ± s.e.m., means ± s.d. or medians. Relevant statistical tests are described in the figure legends. *P* values for comparisons between two groups are two-tailed. GraphPad Prism was used to carry out all statistical analyses.

### Reporting summary

Further information on research design is available in the Nature Portfolio Reporting Summary linked to this article.

## Online content

Any methods, additional references, Nature Portfolio reporting summaries, source data, extended data, supplementary information, acknowledgements, peer review information; details of author contributions and competing interests; and statements of data and code availability are available at 10.1038/s41589-024-01554-z.

### Supplementary information

Supplementary Information Supplementary Tables 1 and 2.

Reporting Summary

Supplementary Video 1 Dynamics of a membrane-binding sequence of *Tn*NinaB inferred from diverse conformations observed within the crystallographic asymmetric unit. The movie begins with a view of the structure in cartoon representation down the β-propeller axis. The iron center is shown as a brown sphere. The view rotates to highlight the membrane-binding surface of the protein and then focuses on the dynamic sequence composed of residues 112–130 (shown as sticks). Here and throughout the rest of the movie, the order of structure morphing is from chain F to chain B to chain H. The structure rotates to highlight the amphipathic nature of the mobile α-helix. The view then focuses on the ‘PDPC(+)’ motif of the mobile sequence which is observed to undergo a hinge motion during the morph. The docked zeaxanthin is then displayed, and the structure is represented as a molecule surface. The morph demonstrates the movement of the mobile helix toward the active site opening.

Supplementary Data 1 PDB validation report for the NinaB crystal structure (PDB accession code 8FTY).

### Source data

Source Data Fig. 1 Statistical source data.

Source Data Fig. 3 Statistical source data and unprocessed gel image.

Source Data Fig. 4 Statistical source data and unprocessed gel images.

Source Data Extended Data Fig. 1 Unprocessed gel images.

Source Data Extended Data Fig. 2 Unprocessed gel images.

Source Data Extended Data Fig. 3 Statistical source data.

Source Data Extended Data Fig. 5 Statistical source data.

Source Data Extended Data Fig. 8 Statistical source data.

## Data Availability

The data that support the findings of this study are available within the main text, extended data figures and Supplementary Information file. Data are also available from the corresponding author upon request. The Non-Redundant Protein Sequence Database within NCBI Protein BLAST was used for identification and analysis of putative NinaB sequences (https://blast.ncbi.nlm.nih.gov/Blast.cgi?PAGE=Proteins). The NinaB crystallographic model and associated diffraction data are available in the Protein Data Bank (PDB) under accession code 8FTY. Other structures used in this paper are available under accession codes 4RSE and 6VCH. Source data are provided with this paper.
