# Supplementary material for: Carotenoid cleavage enzymes evolved convergently to generate the visual chromophore
Source: Nat Chem Biol. 2024 Feb 14;20(6):779–88. doi: 10.1038/s41589-024-01554-z (PMC11142922; doi:10.1038/s41589-024-01554-z)
Supplement: Supplementary file 1 — Supplementary Tables 1 and 2. [file 41589_2024_1554_MOESM1_ESM.pdf]

nature chemical biology

Article https://doi.org/10.1038/s41589-024-01554-z

Carotenoid cleavage enzymes evolved convergently to generate the visual chromophore

In the format provided by the authors and unedited

TABLE OF CONTENTS

CONTENT TYPE	PAGE
**Supplementary Tables**	
Supplementary Table 1	2
Supplementary Table 2	3

Supplementary Table 1. NinaB homologs examined in this study and selected properties

Accession code	Organism Common Name	Organism Scientific name	% Identity to *Gm*NinaB	Soluble expression level[†]
**A8Y9I2.1**	Greater wax moth	*Galleria mellonella*	100	+
**KPI96810.1**	Asian swallowtail butterfly	*Papilio xuthus*	73.4	+
**XP_030020860.1**	Hornworm	*Manduca sexta*	79.9	++
**CAB3223900.1**	Wood tiger moth	*Arctia plantaginis*	76.6	-
**XP_013185217.1**	Navel orangeworm	*Amyelois transitella*	80.6	+
**XP_026733290.1**	Cabbage looper	*Trichoplusia ni*	74.7	++++
**NP_001266310.1**	Silk moth	*Bombyx mori*	76.5	++
**XP_022814551.1**	Tobacco cutworm	*Spodoptera litura*	72.5	++++
**XP_046387976.1**	Blue-tailed damselfly	*Ischnura elegans*	57.7	-
**XP_043598764.1**	Bumble bee	*Bombus pyrosoma*	56.1	-
**EFX87306.1**	Water flea	*Daphnia pulex*	50.8	-
**XP_037285179.1**	Asian blue tick	*Rhipicephalus microplus*	36.3	-
**EEC14724.1**	Deer tick	*Ixodes scapularis*	38.5	-
**XP_023238522.1**	Arizona bark scorpion	*Centruroides sculpturatus*	39.6	-
**GBM92910.1**	European garden spider	*Araneus ventricosus*	38.1	+[1]
**XP_021002585.2**	Common house spider	*Parasteatoda tepidariorum*	40.1	+[1]
**XP_013780683.1**	Horseshoe crab	*Limulus polyphemus b*	39.2	-

[†] The '+' symbols denote a qualitative assessment of target band intensity while '-' indicates no soluble expression was observed.

[1] Minor soluble expression was observed in the chaperone co-expression system

Supplementary Table 2. X-ray diffraction data collection and structure refinement statistics

	*Tn*NinaB †
**Data collection**	
Space group	*C*2
Cell dimensions	
a, *b*, *c* (Å)	349.93, 52.04, 210.04
α, β, γ (°)	90.00, 98.95, 90.00
Resolution (Å)	50 – 1.95 (2.07 – 1.95) *
R_{merge}	0.152 (1.401)
$I / \sigma I$	5.6 (0.9)
Completeness (%)	98.6 (95.7)
Redundancy	3.9 (3.8)
**Refinement**	
Resolution (Å)	50 – 1.95
No. reflections	257,803
R_{work} / R_{free}	21.7/24.7
No. atoms	33,838
Protein	31,630
Ligand/ion	8 (FE2), 120 (MPD)
Water	2,071
B-factors (Å^2)	38.7
Protein	38.6
Ligand/ion	27.7 (FE2), 50.6 (MPD)
Water	39.3
R.m.s. deviations	
Bond lengths (Å)	0.003
Bond angles (°)	0.844

† The dataset was obtained from a single crystal
* Values in parentheses are for the highest-resolution data shell
