# Supplementary material for: Carotenoid cleavage enzymes evolved convergently to generate the visual chromophore
Source: Nat Chem Biol. 2024 Feb 14;20(6):779–88. doi: 10.1038/s41589-024-01554-z (PMC11142922; doi:10.1038/s41589-024-01554-z)
Supplement: Supplementary file 2 — Reporting Summary [file 41589_2024_1554_MOESM2_ESM.pdf]

nature portfolio

Corresponding author(s): Philip D. Kiser

Last updated by author(s): 12/28/2023

Reporting Summary

Nature Portfolio wishes to improve the reproducibility of the work that we publish. This form provides structure for consistency and transparency in reporting. For further information on Nature Portfolio policies, see our Editorial Policies and the Editorial Policy Checklist.

Statistics

For all statistical analyses, confirm that the following items are present in the figure legend, table legend, main text, or Methods section.

n/a	Confirmed	
☐	☒	The exact sample size (*n*) for each experimental group/condition, given as a discrete number and unit of measurement
☐	☒	A statement on whether measurements were taken from distinct samples or whether the same sample was measured repeatedly
☐	☒	The statistical test(s) used AND whether they are one- or two-sided<br>*Only common tests should be described solely by name; describe more complex techniques in the Methods section.*
☒	☐	A description of all covariates tested
☐	☒	A description of any assumptions or corrections, such as tests of normality and adjustment for multiple comparisons
☐	☒	A full description of the statistical parameters including central tendency (e.g. means) or other basic estimates (e.g. regression coefficient) AND variation (e.g. standard deviation) or associated estimates of uncertainty (e.g. confidence intervals)
☐	☒	For null hypothesis testing, the test statistic (e.g. *F*, *t*, *r*) with confidence intervals, effect sizes, degrees of freedom and *P* value noted<br>*Give P values as exact values whenever suitable.*
☒	☐	For Bayesian analysis, information on the choice of priors and Markov chain Monte Carlo settings
☒	☐	For hierarchical and complex designs, identification of the appropriate level for tests and full reporting of outcomes
☒	☐	Estimates of effect sizes (e.g. Cohen's *d*, Pearson's *r*), indicating how they were calculated

Our web collection on statistics for biologists contains articles on many of the points above.

Software and code

Policy information about availability of computer code

Data collection

no new software was developed for data collection.
The following software were used for data collection.
OpenLab CDS Chemstation Edition [Rev. C.01.10(287), Agilent], Lambda Bio XLS Report Viewer (Version 1.11.1.1, Perkin Elmer)

Data analysis

no new software was developed for data analysis. The following software were used for data analysis.
XDS: v20210323
CCP4 webserver: v 1.1.1
MRBUMP: v2
Phaser v2.8.3
ARP/wARP v7
Refmac5: v5.8.0411
Phenix 1.18.2-3874
Coot: v0.9.8
Molprobity server: v4.5
wwPDB validation pipeline: v2.31.3
Autodock Vina v1.1.2
AutoDockTools v1.5.7p1
Pymol v2.5.2
GraphPad Prism v10.0.3
PhyML v3.3.20220408
Weblogo v2.8.2

Muscle v5
Clustal omega v1.2.4
Protein Blast web server and Non-redundant Protein Sequence Database (https://blast.ncbi.nlm.nih.gov/Blast.cgi?PAGE=Proteins). Database release 20230104

For manuscripts utilizing custom algorithms or software that are central to the research but not yet described in published literature, software must be made available to editors and reviewers. We strongly encourage code deposition in a community repository (e.g. GitHub). See the Nature Portfolio guidelines for submitting code & software for further information.

Data

Policy information about availability of data

All manuscripts must include a data availability statement. This statement should provide the following information, where applicable:

- Accession codes, unique identifiers, or web links for publicly available datasets
- A description of any restrictions on data availability
- For clinical datasets or third party data, please ensure that the statement adheres to our policy

The data that support the findings of this study are available within the main text, extended data figures, supplementary tables, and Source Data files included with this manuscript. Data are also available from the corresponding author upon request. This study used the Non-Redundant Protein Sequence Database within NCBI Protein BLAST for identification and analysis of putative NinaB sequences (https://blast.ncbi.nlm.nih.gov/Blast.cgi?PAGE=Proteins).The NinaB crystal structure model and associated diffraction data generated in this study are available in the Protein Data Bank (PDB) under accession code: 8FTY (https://www.rcsb.org/structure/8FTY)

Other crystals structures used in this study are available from the PDB under accession codes: 6VCH (NdCCD), 4RSE (bovine RPE65 in complex with MB-001 and palmitate)

https://www.rcsb.org/structure/6VCH
https://www.rcsb.org/structure/4RSE

Human research participants

Policy information about studies involving human research participants and Sex and Gender in Research.

Reporting on sex and gender	n/a
Population characteristics	n/a
Recruitment	n/a
Ethics oversight	n/a

Note that full information on the approval of the study protocol must also be provided in the manuscript.

Field-specific reporting

Please select the one below that is the best fit for your research. If you are not sure, read the appropriate sections before making your selection.

☒ Life sciences ☐ Behavioural & social sciences ☐ Ecological, evolutionary & environmental sciences

For a reference copy of the document with all sections, see nature.com/documents/nr-reporting-summary-flat.pdf

Life sciences study design

All studies must disclose on these points even when the disclosure is negative.

Sample size	The sample sizes chosen reflect our past experience with the in vitro and cell-based experiments used in this study and were sufficient to assess replicability and allow statistical hypothesis testing. The number of independent experiments performed are specified in the figure legends.
Data exclusions	No data were excluded.
Replication	Multiple independent experiments were performed to ensure replicability of the results. All experimental results reported in the paper were successfully replicated. The number of independent experiments is specified in the figure legends.

Randomization	Randomization procedures were not used in this study since no risk of errors associated with systematic selection bias was expected.
Blinding	Blinding procedures were not used in this study because no risk of errors associated systematic selection bias or observer bias was expected

Reporting for specific materials, systems and methods

We require information from authors about some types of materials, experimental systems and methods used in many studies. Here, indicate whether each material, system or method listed is relevant to your study. If you are not sure if a list item applies to your research, read the appropriate section before selecting a response.

Materials & experimental systems

n/a	Involved in the study
☒	☐ Antibodies
☒	☐ Eukaryotic cell lines
☒	☐ Palaeontology and archaeology
☒	☐ Animals and other organisms
☒	☐ Clinical data
☒	☐ Dual use research of concern

Methods

n/a	Involved in the study
☒	☐ ChIP-seq
☒	☐ Flow cytometry
☒	☐ MRI-based neuroimaging
