# Supplementary material for: Carotenoid cleavage enzymes evolved convergently to generate the visual chromophore
Source: Nat Chem Biol. 2024 Feb 14;20(6):779–88. doi: 10.1038/s41589-024-01554-z (PMC11142922; doi:10.1038/s41589-024-01554-z)
Supplement: Supplementary file 4 — PDB validation report for the NinaB crystal structure (PDB accession code 8FTY). [file 41589_2024_1554_MOESM4_ESM.pdf]

Full wwPDB X-ray Structure Validation Report (i)

Sep 19, 2023 – 03:04 PM EDT

PDB ID	:	8FTY
Title	:	Crystal structure of the carotenoid isomerooxygenase, NinaB
Deposited on	:	2023-01-14
Resolution	:	1.95 Å(reported)

This wwPDB validation report is for manuscript review

For Manuscript Review

This is a Full wwPDB X-ray Structure Validation Report.

This report is produced by the wwPDB biocuration pipeline after annotation of the structure.

We welcome your comments at *validation@mail.wwpdb.org*
A user guide is available at
https://www.wwpdb.org/validation/2017/XrayValidationReportHelp
with specific help available everywhere you see the (i) symbol.

The types of validation reports are described at
http://www.wwpdb.org/validation/2017/FAQs#types.

The following versions of software and data (see references (i)) were used in the production of this report:

MolProbity	:	4.02b-467
Mogul	:	1.8.5 (274361), CSD as541be (2020)
Xtriage (Phenix)	:	1.13
EDS	:	2.35.1
buster-report	:	1.1.7 (2018)
Percentile statistics	:	20191225.v01 (using entries in the PDB archive December 25th 2019)
Refmac	:	5.8.0158
CCP4	:	7.0.044 (Gargrove)
Ideal geometry (proteins)	:	Engh & Huber (2001)
Ideal geometry (DNA, RNA)	:	Parkinson et al. (1996)

1 Overall quality at a glance (i)

The following experimental techniques were used to determine the structure:
X-RAY DIFFRACTION

The reported resolution of this entry is 1.95 Å.

Percentile scores (ranging between 0-100) for global validation metrics of the entry are shown in the following graphic. The table shows the number of entries on which the scores are based.

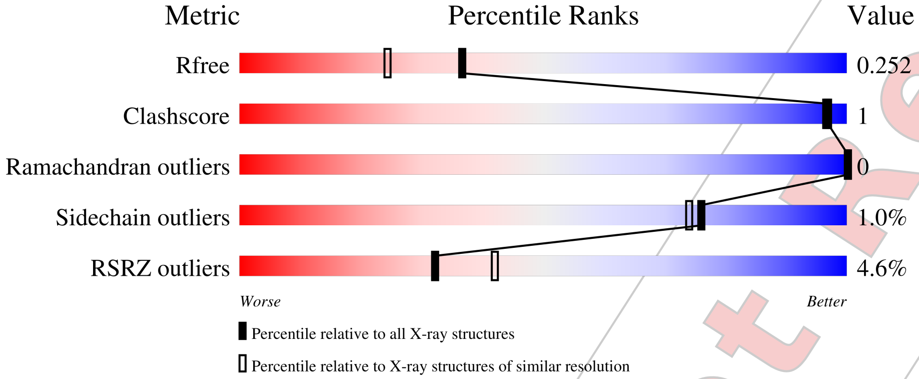

Metric	Whole archive (#Entries)	Similar resolution (#Entries, resolution range(Å))
R_{free}	130704	2580 (1.96-1.96)
Clashscore	141614	2705 (1.96-1.96)
Ramachandran outliers	138981	2678 (1.96-1.96)
Sidechain outliers	138945	2678 (1.96-1.96)
RSRZ outliers	127900	2539 (1.96-1.96)

The table below summarises the geometric issues observed across the polymeric chains and their fit to the electron density. The red, orange, yellow and green segments of the lower bar indicate the fraction of residues that contain outliers for >=3, 2, 1 and 0 types of geometric quality criteria respectively. A grey segment represents the fraction of residues that are not modelled. The numeric value for each fraction is indicated below the corresponding segment, with a dot representing fractions <=5% The upper red bar (where present) indicates the fraction of residues that have poor fit to the electron density. The numeric value is given above the bar.

Mol	Chain	Length	Quality of chain
1	A	509	4% / 92% • 5%
1	B	509	5% / 95% • •
1	C	509	4% / 91% • 5%
1	D	509	4% / 95% • •

Continued on next page...

For Manuscript Review

Continued from previous page...

Mol	Chain	Length	Quality of chain
2	E	509	2% 94% • •
2	F	509	2% 96% • •
3	G	509	6% 93% • •
3	H	509	9% 94% 5% •

For Manuscript Review

2 Entry composition (i)

There are 9 unique types of molecules in this entry. The entry contains 33838 atoms, of which 0 are hydrogens and 0 are deuteriums.

In the tables below, the ZeroOcc column contains the number of atoms modelled with zero occupancy, the AltConf column contains the number of residues with at least one atom in alternate conformation and the Trace column contains the number of residues modelled with at most 2 atoms.

- Molecule 1 is a protein called carotenoid isomerooxygenase.

Mol	Chain	Residues	Atoms					ZeroOcc	AltConf	Trace
			Total	C	N	O	S			
1	A	483	3851	2453	653	715	30	0	2	0
1	B	506	4039	2572	686	749	32	0	3	0
1	C	482	3866	2460	658	716	32	0	5	0
1	D	497	3976	2534	676	734	32	0	5	0

- Molecule 2 is a protein called carotenoid isomerooxygenase.

Mol	Chain	Residues	Atoms					ZeroOcc	AltConf	Trace
			Total	C	N	O	S			
2	E	494	3957	2520	674	731	32	0	4	0
2	F	501	4050	2579	691	746	34	0	9	0

- Molecule 3 is a protein called carotenoid isomerooxygenase.

Mol	Chain	Residues	Atoms					ZeroOcc	AltConf	Trace
			Total	C	N	O	S			
3	G	490	3887	2472	663	721	31	0	2	0
3	H	502	4004	2550	682	741	31	0	3	0

- Molecule 4 is FE (II) ION (three-letter code: FE2) (formula: Fe) (labeled as "Ligand of Interest" by depositor).

Mol	Chain	Residues	Atoms		ZeroOcc	AltConf
			Total	Fe		
4	A	1	1	1	0	0

Continued on next page...

For Manuscript Review

Continued from previous page...

Mol	Chain	Residues	Atoms	ZeroOcc	AltConf
4	B	1	Total Fe<br>1 1	0	0
4	C	1	Total Fe<br>1 1	0	0
4	D	1	Total Fe<br>1 1	0	0
4	E	1	Total Fe<br>1 1	0	0
4	F	1	Total Fe<br>1 1	0	0
4	G	1	Total Fe<br>1 1	0	0
4	H	1	Total Fe<br>1 1	0	0

- Molecule 5 is (4S)-2-METHYL-2,4-PENTANEDIOL (three-letter code: MPD) (formula: $C_6H_{14}O_2$) (labeled as "Ligand of Interest" by depositor).

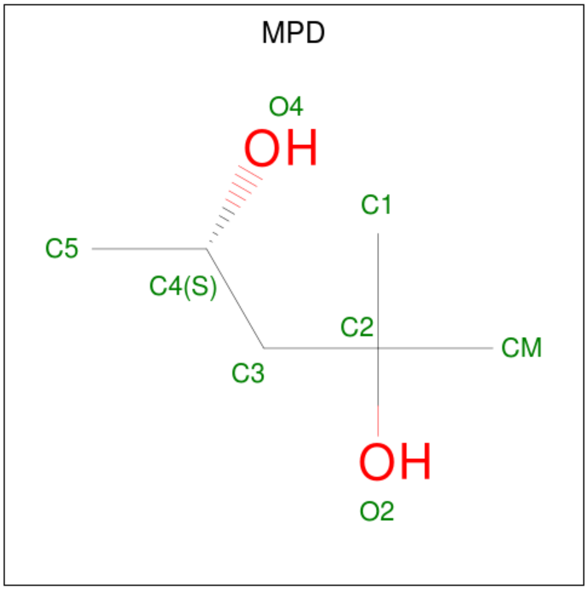

Mol	Chain	Residues	Atoms	ZeroOcc	AltConf
5	A	1	Total C O<br>8 6 2	0	0
5	A	1	Total C O<br>8 6 2	0	0
5	B	1	Total C O<br>8 6 2	0	0
5	C	1	Total C O<br>8 6 2	0	0

Continued on next page...

For Manuscript Review

Continued from previous page...

Mol	Chain	Residues	Atoms	ZeroOcc	AltConf
5	C	1	Total C O<br>8 6 2	0	0
5	D	1	Total C O<br>8 6 2	0	0
5	D	1	Total C O<br>8 6 2	0	0
5	E	1	Total C O<br>8 6 2	0	0
5	E	1	Total C O<br>8 6 2	0	0
5	E	1	Total C O<br>8 6 2	0	0
5	F	1	Total C O<br>8 6 2	0	0
5	F	1	Total C O<br>8 6 2	0	0
5	G	1	Total C O<br>8 6 2	0	0
5	G	1	Total C O<br>8 6 2	0	0
5	H	1	Total C O<br>8 6 2	0	0

- Molecule 6 is CHLORIDE ION (three-letter code: CL) (formula: Cl).

Mol	Chain	Residues	Atoms	ZeroOcc	AltConf
6	A	1	Total Cl<br>1 1	0	0
6	D	1	Total Cl<br>1 1	0	0
6	E	1	Total Cl<br>1 1	0	0

- Molecule 7 is SODIUM ION (three-letter code: NA) (formula: Na).

Mol	Chain	Residues	Atoms	ZeroOcc	AltConf
7	D	1	Total Na<br>1 1	0	0

- Molecule 8 is CACODYLATE ION (three-letter code: CAC) (formula: $C_2H_6AsO_2$).

For Manuscript Review

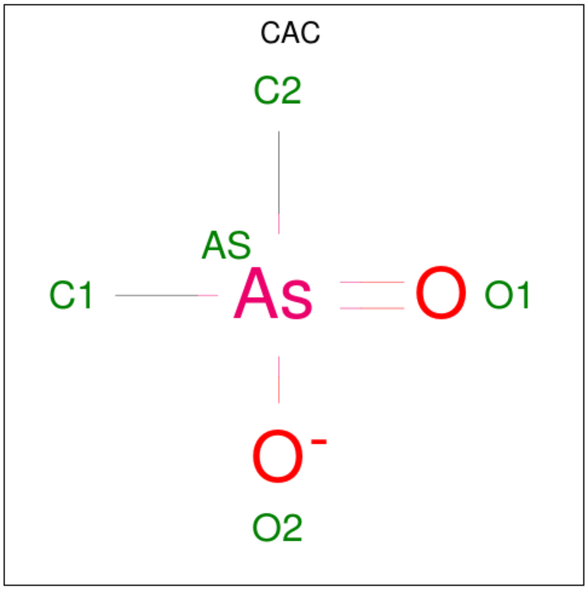

Mol	Chain	Residues	Atoms	ZeroOcc	AltConf
8	F	1	Total As C O<br>5 1 2 2	0	0

- Molecule 9 is water.

Mol	Chain	Residues	Atoms	ZeroOcc	AltConf
9	A	193	Total O<br>193 193	0	0
9	B	314	Total O<br>314 314	0	0
9	C	241	Total O<br>241 241	0	0
9	D	242	Total O<br>242 242	0	0
9	E	333	Total O<br>333 333	0	0
9	F	337	Total O<br>337 337	0	0
9	G	210	Total O<br>210 210	0	0
9	H	201	Total O<br>201 201	0	0

For Manuscript Review

3 Residue-property plots ⓘ

These plots are drawn for all protein, RNA, DNA and oligosaccharide chains in the entry. The first graphic for a chain summarises the proportions of the various outlier classes displayed in the second graphic. The second graphic shows the sequence view annotated by issues in geometry and electron density. Residues are color-coded according to the number of geometric quality criteria for which they contain at least one outlier: green = 0, yellow = 1, orange = 2 and red = 3 or more. A red dot above a residue indicates a poor fit to the electron density (RSRZ > 2). Stretches of 2 or more consecutive residues without any outlier are shown as a green connector. Residues present in the sample, but not in the model, are shown in grey.

- Molecule 1: carotenoid isomerooxygenase

Chain A:

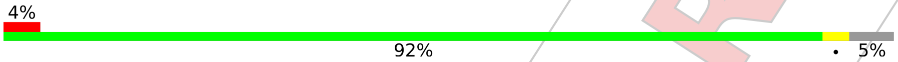

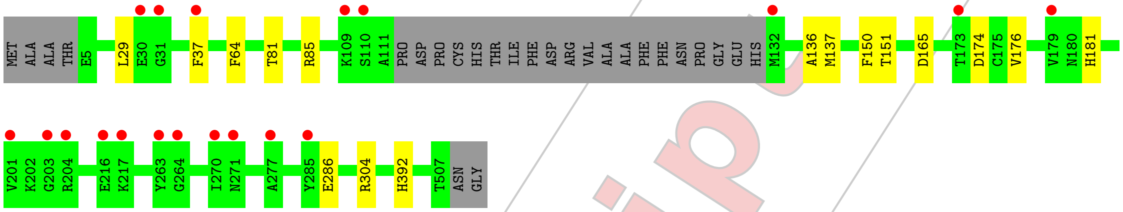

- Molecule 1: carotenoid isomerooxygenase

Chain B:

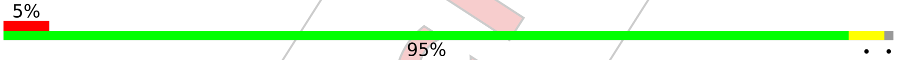

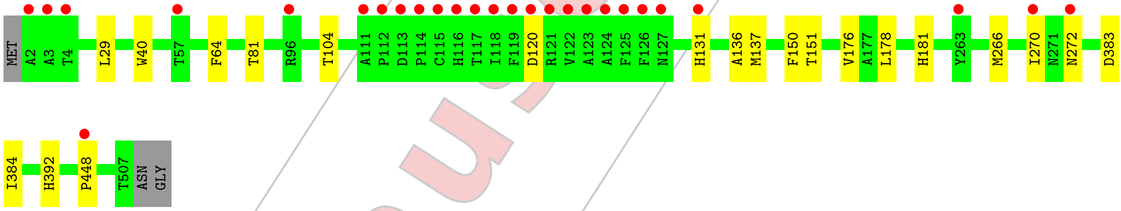

- Molecule 1: carotenoid isomerooxygenase

Chain C:

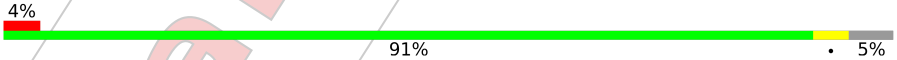

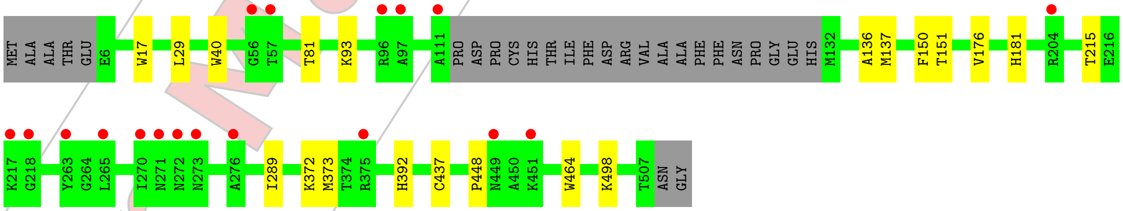

- Molecule 1: carotenoid isomerooxygenase

Chain D:

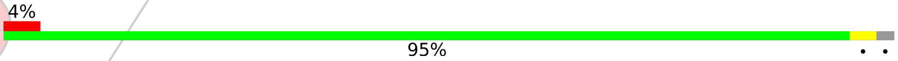

For Manuscript Review

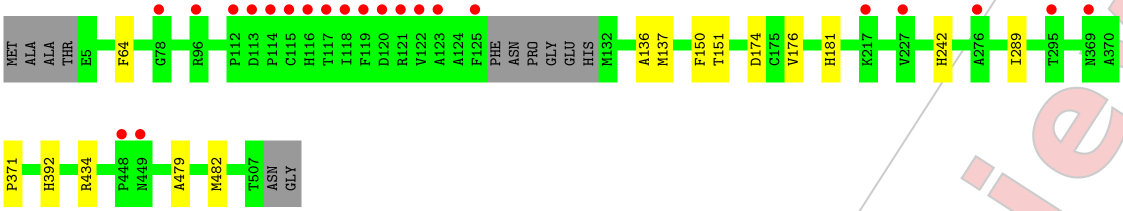

• Molecule 2: carotenoid isomerooxygenase

Chain E:

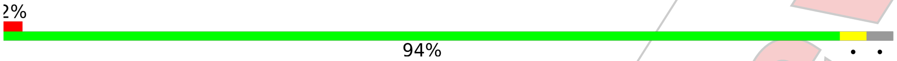

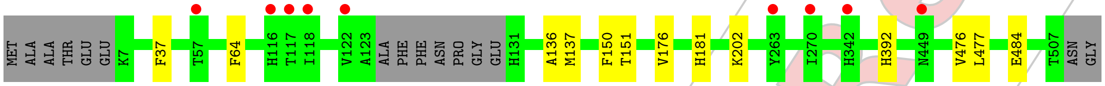

• Molecule 2: carotenoid isomerooxygenase

Chain F:

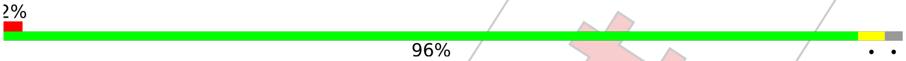

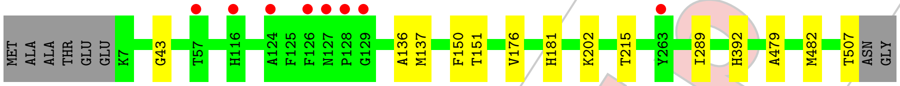

• Molecule 3: carotenoid isomerooxygenase

Chain G:

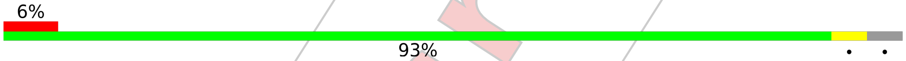

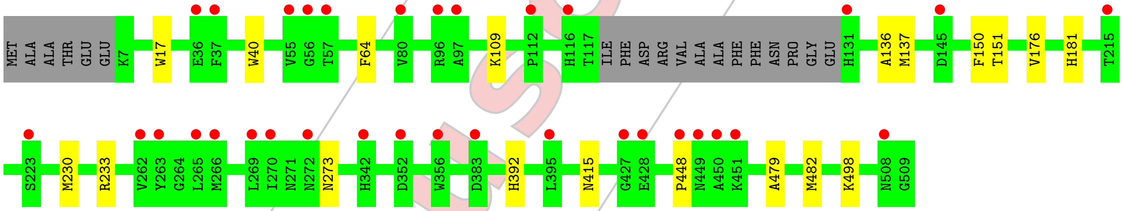

• Molecule 3: carotenoid isomerooxygenase

Chain H:

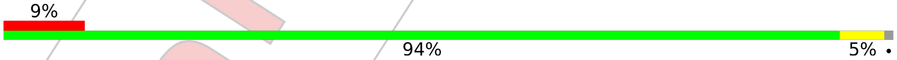

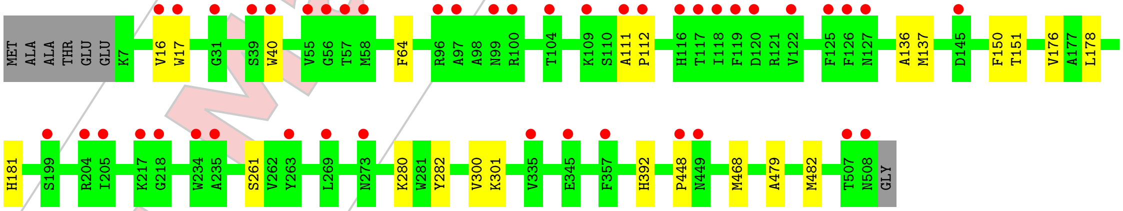

For Manuscript Review

4 Data and refinement statistics (i)

Property	Value	Source
Space group	C 1 2 1	Depositor
Cell constants<br>a, b, c, α, β, γ	349.93Å 52.04Å 210.04Å<br>90.00° 98.95° 90.00°	Depositor
Resolution (Å)	49.67 – 1.95<br>49.68 – 1.95	Depositor<br>EDS
% Data completeness<br>(in resolution range)	98.6 (49.67-1.95)<br>98.6 (49.68-1.95)	Depositor<br>EDS
R_{merge}	0.15	Depositor
R_{sym}	(Not available)	Depositor
$< I/\sigma(I) >$ [1]	1.04 (at 1.95Å)	Xtriage
Refinement program	REFMAC 5.8.0411	Depositor
R, R_{free}	0.217 , 0.247<br>0.224 , 0.252	Depositor<br>DCC
R_{free} test set	13352 reflections (4.92%)	wwPDB-VP
Wilson B-factor (Å^2)	33.8	Xtriage
Anisotropy	0.487	Xtriage
Bulk solvent k_{sol}(e/Å^3), B_{sol}(Å^2)	0.33 , 40.1	EDS
L-test for twinning[2]	$< \vert L \vert > = 0.51$, $< L^2 > = 0.35$	Xtriage
Estimated twinning fraction	No twinning to report.	Xtriage
F_o,F_c correlation	0.96	EDS
Total number of atoms	33838	wwPDB-VP
Average B, all atoms (Å^2)	38.0	wwPDB-VP

Xtriage's analysis on translational NCS is as follows: *The analyses of the Patterson function reveals a significant off-origin peak that is 54.12 % of the origin peak, indicating pseudo-translational symmetry. The chance of finding a peak of this or larger height randomly in a structure without pseudo-translational symmetry is equal to 3.8251e-05. The detected translational NCS is most likely also responsible for the elevated intensity ratio.*

[1] Intensities estimated from amplitudes.

[2] Theoretical values of $< |L| >$, $< L^2 >$ for acentric reflections are 0.5, 0.333 respectively for untwinned datasets, and 0.375, 0.2 for perfectly twinned datasets.

For Manuscript Review

5 Model quality (i)

5.1 Standard geometry (i)

Bond lengths and bond angles in the following residue types are not validated in this section: CAC, MPD, CSO, CL, CSD, NA, FE2

The Z score for a bond length (or angle) is the number of standard deviations the observed value is removed from the expected value. A bond length (or angle) with $|Z| > 5$ is considered an outlier worth inspection. RMSZ is the root-mean-square of all Z scores of the bond lengths (or angles).

Mol	Chain	Bond lengths		Bond angles	
		RMSZ	#|Z| >5	RMSZ	#|Z| >5
1	A	0.26	0/3949	0.49	0/5367
1	B	0.27	0/4146	0.50	0/5639
1	C	0.26	0/3964	0.49	0/5387
1	D	0.27	0/4080	0.49	0/5550
2	E	0.27	0/4052	0.50	0/5508
2	F	0.27	0/4150	0.49	0/5640
3	G	0.27	0/3981	0.49	0/5416
3	H	0.26	0/4105	0.49	0/5588
All	All	0.27	0/32427	0.49	0/44095

Chiral center outliers are detected by calculating the chiral volume of a chiral center and verifying if the center is modelled as a planar moiety or with the opposite hand.A planarity outlier is detected by checking planarity of atoms in a peptide group, atoms in a mainchain group or atoms of a sidechain that are expected to be planar.

Mol	Chain	#Chirality outliers	#Planarity outliers
1	A	0	1
1	B	0	1
1	C	0	1
1	D	0	1
2	E	0	1
2	F	0	1
3	G	0	1
3	H	0	1
All	All	0	8

There are no bond length outliers.

There are no bond angle outliers.

There are no chirality outliers.

All (8) planarity outliers are listed below:

For Manuscript Review

Mol	Chain	Res	Type	Group
1	A	150	PHE	Peptide
1	B	150	PHE	Peptide
1	C	150	PHE	Peptide
1	D	150	PHE	Peptide
2	E	150	PHE	Peptide
2	F	150	PHE	Peptide
3	G	150	PHE	Peptide
3	H	150	PHE	Peptide

5.2 Too-close contacts ⓘ

In the following table, the Non-H and H(model) columns list the number of non-hydrogen atoms and hydrogen atoms in the chain respectively. The H(added) column lists the number of hydrogen atoms added and optimized by MolProbity. The Clashes column lists the number of clashes within the asymmetric unit, whereas Symm-Clashes lists symmetry-related clashes.

Mol	Chain	Non-H	H(model)	H(added)	Clashes	Symm-Clashes
1	A	3851	0	3777	6	0
1	B	4039	0	3947	9	0
1	C	3866	0	3788	8	0
1	D	3976	0	3879	8	0
2	E	3957	0	3880	7	0
2	F	4050	0	3952	5	0
3	G	3887	0	3787	6	0
3	H	4004	0	3887	12	0
4	A	1	0	0	0	0
4	B	1	0	0	0	0
4	C	1	0	0	0	0
4	D	1	0	0	0	0
4	E	1	0	0	0	0
4	F	1	0	0	0	0
4	G	1	0	0	0	0
4	H	1	0	0	0	0
5	A	16	0	28	1	0
5	B	8	0	14	1	0
5	C	16	0	28	0	0
5	D	16	0	28	2	0
5	E	24	0	42	1	0
5	F	16	0	28	0	0
5	G	16	0	28	1	0
5	H	8	0	14	1	0
6	A	1	0	0	0	0
6	D	1	0	0	0	0

Continued on next page...

For Manuscript Review

Continued from previous page...

Mol	Chain	Non-H	H(model)	H(added)	Clashes	Symm-Clashes
6	E	1	0	0	0	0
7	D	1	0	0	0	0
8	F	5	0	0	0	0
9	A	193	0	0	0	0
9	B	314	0	0	0	0
9	C	241	0	0	1	0
9	D	242	0	0	1	0
9	E	333	0	0	0	0
9	F	337	0	0	0	0
9	G	210	0	0	0	0
9	H	201	0	0	0	0
All	All	33838	0	31107	58	0

The all-atom clashscore is defined as the number of clashes found per 1000 atoms (including hydrogen atoms). The all-atom clashscore for this structure is 1.

All (58) close contacts within the same asymmetric unit are listed below, sorted by their clash magnitude.

Atom-1	Atom-2	Interatomic distance (Å)	Clash overlap (Å)
1:A:64:PHE:CZ	5:A:602:MPD:H31	2.36	0.60
1:A:174:ASP:O	2:E:202:LYS:HA	2.04	0.58
3:H:64:PHE:CZ	5:H:1001:MPD:H31	2.40	0.56
1:A:286:GLU:OE1	1:A:304:ARG:HD2	2.07	0.54
1:D:434:ARG:NH2	9:D:2102:HOH:O	2.40	0.54
1:B:64:PHE:CZ	5:B:1001:MPD:H31	2.43	0.53
1:D:242:HIS:CE1	5:D:602:MPD:O2	2.62	0.53
2:E:64:PHE:CZ	5:E:602:MPD:H31	2.46	0.51
3:H:111:ALA:HB1	3:H:112:PRO:HD2	1.93	0.51
1:B:29:LEU:O	1:B:81:THR:HA	2.11	0.50
3:H:261:SER:HB2	3:H:280:LYS:HD2	1.94	0.50
1:D:174:ASP:O	2:F:202:LYS:HA	2.11	0.50
1:C:29:LEU:O	1:C:81:THR:HA	2.11	0.50
1:B:104:THR:OG1	1:B:272:ASN:OD1	2.24	0.49
1:C:437[B]:CYS:SG	1:C:464:TRP:NE1	2.87	0.48
1:D:64:PHE:CZ	5:D:602:MPD:H31	2.49	0.48
1:D:479:ALA:HA	1:D:482[A]:MET:CE	2.44	0.48
1:B:266:MET:O	1:B:270:ILE:HG12	2.14	0.47
1:B:176:VAL:HG13	1:B:178:LEU:HG	1.96	0.47
3:H:176:VAL:HG22	3:H:176:VAL:O	2.15	0.47
2:E:37:PHE:CE2	2:E:476[A]:VAL:HG21	2.49	0.47
3:H:280:LYS:HD3	3:H:282:TYR:OH	2.16	0.46

Continued on next page...

For Manuscript Review

Continued from previous page...

Atom-1	Atom-2	Interatomic distance (Å)	Clash overlap (Å)
1:B:40:TRP:CD2	1:B:448:PRO:HD3	2.51	0.46
3:H:136:ALA:HA	3:H:151:THR:HB	1.98	0.46
1:C:17:TRP:CZ2	1:C:498:LYS:HE3	2.51	0.45
3:H:176:VAL:HG13	3:H:178:LEU:HG	1.96	0.45
2:E:37:PHE:CE2	2:E:476[A]:VAL:CG2	2.99	0.45
3:G:109:LYS:HG3	3:G:273:ASN:HA	1.98	0.45
3:H:300:VAL:HG12	3:H:301:LYS:HG3	1.99	0.45
1:B:136:ALA:HA	1:B:151:THR:HB	1.99	0.44
3:G:17:TRP:CZ2	3:G:498:LYS:HE3	2.53	0.44
1:D:371:PRO:HB2	3:H:468:MET:CE	2.47	0.44
2:F:136:ALA:HA	2:F:151:THR:HB	2.00	0.44
3:H:40:TRP:CD2	3:H:448:PRO:HD3	2.52	0.44
2:E:136:ALA:HA	2:E:151:THR:HB	2.00	0.43
1:A:85:ARG:NH1	1:A:165:ASP:OD1	2.47	0.43
1:D:136:ALA:HA	1:D:151:THR:HB	2.00	0.43
3:G:64:PHE:CZ	5:G:602:MPD:H31	2.53	0.43
1:A:136:ALA:HA	1:A:151:THR:HB	2.00	0.42
3:G:136:ALA:HA	3:G:151:THR:HB	2.01	0.42
1:C:372:LYS:O	1:C:373:MET:HB2	2.19	0.42
2:F:43:GLY:HA2	2:F:507:THR:HG22	2.02	0.42
2:E:37:PHE:CZ	2:E:476[A]:VAL:HG23	2.55	0.42
2:E:477:LEU:HD23	2:E:484:GLU:HA	2.01	0.42
3:H:479:ALA:HA	3:H:482[A]:MET:CE	2.50	0.42
2:F:479:ALA:HA	2:F:482[A]:MET:CE	2.50	0.42
1:A:29:LEU:O	1:A:81:THR:HA	2.19	0.42
1:B:383:ASP:O	1:B:384:ILE:HG23	2.19	0.42
1:C:136:ALA:HA	1:C:151:THR:HB	2.00	0.42
3:H:16:VAL:HG22	3:H:17[B]:TRP:CE3	2.55	0.42
1:B:120:ASP:OD1	1:B:131:HIS:NE2	2.46	0.41
1:C:40:TRP:CD2	1:C:448:PRO:HD3	2.55	0.41
3:G:479:ALA:HA	3:G:482:MET:CE	2.50	0.41
1:C:289:ILE:HD12	1:C:289:ILE:N	2.36	0.41
3:G:40:TRP:CD2	3:G:448:PRO:HD3	2.55	0.41
2:F:289:ILE:HD12	2:F:289:ILE:N	2.36	0.41
1:C:93:LYS:HG3	9:C:1310:HOH:O	2.20	0.40
1:D:289:ILE:HD12	1:D:289:ILE:N	2.37	0.40

There are no symmetry-related clashes.

For Manuscript Review

5.3 Torsion angles (i)

5.3.1 Protein backbone (i)

In the following table, the Percentiles column shows the percent Ramachandran outliers of the chain as a percentile score with respect to all X-ray entries followed by that with respect to entries of similar resolution.

The Analysed column shows the number of residues for which the backbone conformation was analysed, and the total number of residues.

Mol	Chain	Analysed	Favoured	Allowed	Outliers	Percentiles	
1	A	481/509 (94%)	467 (97%)	14 (3%)	0	100	100
1	B	507/509 (100%)	492 (97%)	15 (3%)	0	100	100
1	C	483/509 (95%)	469 (97%)	14 (3%)	0	100	100
1	D	498/509 (98%)	481 (97%)	17 (3%)	0	100	100
2	E	493/509 (97%)	479 (97%)	14 (3%)	0	100	100
2	F	507/509 (100%)	491 (97%)	16 (3%)	0	100	100
3	G	487/509 (96%)	472 (97%)	15 (3%)	0	100	100
3	H	502/509 (99%)	487 (97%)	15 (3%)	0	100	100
All	All	3958/4072 (97%)	3838 (97%)	120 (3%)	0	100	100

There are no Ramachandran outliers to report.

5.3.2 Protein sidechains (i)

In the following table, the Percentiles column shows the percent sidechain outliers of the chain as a percentile score with respect to all X-ray entries followed by that with respect to entries of similar resolution.

The Analysed column shows the number of residues for which the sidechain conformation was analysed, and the total number of residues.

Mol	Chain	Analysed	Rotameric	Outliers	Percentiles	
1	A	425/444 (96%)	420 (99%)	5 (1%)	71	68
1	B	445/444 (100%)	442 (99%)	3 (1%)	84	82
1	C	427/444 (96%)	422 (99%)	5 (1%)	71	68
1	D	437/444 (98%)	433 (99%)	4 (1%)	78	77
2	E	437/443 (99%)	433 (99%)	4 (1%)	78	77
2	F	445/443 (100%)	440 (99%)	5 (1%)	73	71

Continued on next page...

For Manuscript Review

Continued from previous page...

Mol	Chain	Analysed	Rotameric	Outliers	Percentiles	
3	G	426/443 (96%)	419 (98%)	7 (2%)	62	58
3	H	438/443 (99%)	435 (99%)	3 (1%)	84	82
All	All	3480/3548 (98%)	3444 (99%)	36 (1%)	76	74

All (36) residues with a non-rotameric sidechain are listed below:

Mol	Chain	Res	Type
1	A	37	PHE
1	A	137	MET
1	A	176	VAL
1	A	181	HIS
1	A	392	HIS
1	B	137	MET
1	B	181	HIS
1	B	392	HIS
1	C	137	MET
1	C	176	VAL
1	C	181	HIS
1	C	215	THR
1	C	392	HIS
1	D	137	MET
1	D	176	VAL
1	D	181	HIS
1	D	392	HIS
2	E	137	MET
2	E	176	VAL
2	E	181	HIS
2	E	392	HIS
2	F	137	MET
2	F	176	VAL
2	F	181	HIS
2	F	215	THR
2	F	392	HIS
3	G	137	MET
3	G	176	VAL
3	G	181	HIS
3	G	230	MET
3	G	233	ARG
3	G	392	HIS
3	G	415	ASN
3	H	137	MET

Continued on next page...

For Manuscript Review

Continued from previous page...

Mol	Chain	Res	Type
3	H	181	HIS
3	H	392	HIS

Sometimes sidechains can be flipped to improve hydrogen bonding and reduce clashes. All (1) such sidechains are listed below:

Mol	Chain	Res	Type
1	A	237	HIS

5.3.3 RNA ⓘ

There are no RNA molecules in this entry.

5.4 Non-standard residues in protein, DNA, RNA chains ⓘ

4 non-standard protein/DNA/RNA residues are modelled in this entry.

In the following table, the Counts columns list the number of bonds (or angles) for which Mogul statistics could be retrieved, the number of bonds (or angles) that are observed in the model and the number of bonds (or angles) that are defined in the Chemical Component Dictionary. The Link column lists molecule types, if any, to which the group is linked. The Z score for a bond length (or angle) is the number of standard deviations the observed value is removed from the expected value. A bond length (or angle) with $|Z| > 2$ is considered an outlier worth inspection. RMSZ is the root-mean-square of all Z scores of the bond lengths (or angles).

Mol	Type	Chain	Res	Link	Bond lengths			Bond angles		
					Counts	RMSZ	$\#\|Z\| > 2$	Counts	RMSZ	$\#\|Z\| > 2$
3	CSD	H	115	3	3,7,8	0.72	0	1,8,10	0.51	0
2	CSO	E	115	2	3,6,7	0.68	0	0,6,8	-	-
2	CSO	F	115	2	3,6,7	0.72	0	0,6,8	-	-
3	CSD	G	115	3	3,7,8	0.72	0	1,8,10	0.29	0

In the following table, the Chirals column lists the number of chiral outliers, the number of chiral centers analysed, the number of these observed in the model and the number defined in the Chemical Component Dictionary. Similar counts are reported in the Torsion and Rings columns. '-' means no outliers of that kind were identified.

Mol	Type	Chain	Res	Link	Chirals	Torsions	Rings
3	CSD	H	115	3	-	1/2/6/8	-
2	CSO	E	115	2	-	1/1/5/7	-
2	CSO	F	115	2	-	0/1/5/7	-
3	CSD	G	115	3	-	1/2/6/8	-

For Manuscript Review

There are no bond length outliers.

There are no bond angle outliers.

There are no chirality outliers.

All (3) torsion outliers are listed below:

Mol	Chain	Res	Type	Atoms
2	E	115	CSO	N-CA-CB-SG
3	G	115	CSD	CA-CB-SG-OD1
3	H	115	CSD	CA-CB-SG-OD1

There are no ring outliers.

No monomer is involved in short contacts.

5.5 Carbohydrates (i)

There are no monosaccharides in this entry.

5.6 Ligand geometry (i)

Of 28 ligands modelled in this entry, 12 are monoatomic - leaving 16 for Mogul analysis.

In the following table, the Counts columns list the number of bonds (or angles) for which Mogul statistics could be retrieved, the number of bonds (or angles) that are observed in the model and the number of bonds (or angles) that are defined in the Chemical Component Dictionary. The Link column lists molecule types, if any, to which the group is linked. The Z score for a bond length (or angle) is the number of standard deviations the observed value is removed from the expected value. A bond length (or angle) with $|Z| > 2$ is considered an outlier worth inspection. RMSZ is the root-mean-square of all Z scores of the bond lengths (or angles).

Mol	Type	Chain	Res	Link	Bond lengths			Bond angles		
					Counts	RMSZ	$\#\|Z\| > 2$	Counts	RMSZ	$\#\|Z\| > 2$
8	CAC	F	1003	-	0,4,4	-	-	0,6,6	-	-
5	MPD	C	602	-	7,7,7	0.10	0	9,10,10	0.40	0
5	MPD	G	602	-	7,7,7	0.13	0	9,10,10	0.35	0
5	MPD	A	604	-	7,7,7	0.13	0	9,10,10	0.33	0
5	MPD	F	1002	-	7,7,7	0.13	0	9,10,10	0.38	0
5	MPD	E	602	-	7,7,7	0.15	0	9,10,10	0.38	0
5	MPD	B	1001	-	7,7,7	0.12	0	9,10,10	0.36	0
5	MPD	F	1001	-	7,7,7	0.08	0	9,10,10	0.40	0
5	MPD	G	603	-	7,7,7	0.11	0	9,10,10	0.38	0
5	MPD	D	603	-	7,7,7	0.12	0	9,10,10	0.37	0
5	MPD	D	602	-	7,7,7	0.13	0	9,10,10	0.37	0

For Manuscript Review

Mol	Type	Chain	Res	Link	Bond lengths			Bond angles		
					Counts	RMSZ	#|Z| > 2	Counts	RMSZ	#|Z| > 2
5	MPD	E	604	-	7,7,7	0.09	0	9,10,10	0.39	0
5	MPD	E	603	-	7,7,7	0.11	0	9,10,10	0.41	0
5	MPD	C	603	-	7,7,7	0.12	0	9,10,10	0.41	0
5	MPD	H	1001	-	7,7,7	0.13	0	9,10,10	0.36	0
5	MPD	A	602	-	7,7,7	0.14	0	9,10,10	0.38	0

In the following table, the Chirals column lists the number of chiral outliers, the number of chiral centers analysed, the number of these observed in the model and the number defined in the Chemical Component Dictionary. Similar counts are reported in the Torsion and Rings columns. '-' means no outliers of that kind were identified.

Mol	Type	Chain	Res	Link	Chirals	Torsions	Rings
5	MPD	C	602	-	-	0/5/5/5	-
5	MPD	G	602	-	-	1/5/5/5	-
5	MPD	A	604	-	-	0/5/5/5	-
5	MPD	F	1002	-	-	0/5/5/5	-
5	MPD	E	602	-	-	0/5/5/5	-
5	MPD	B	1001	-	-	0/5/5/5	-
5	MPD	F	1001	-	-	0/5/5/5	-
5	MPD	G	603	-	-	1/5/5/5	-
5	MPD	D	603	-	-	0/5/5/5	-
5	MPD	D	602	-	-	0/5/5/5	-
5	MPD	E	604	-	-	0/5/5/5	-
5	MPD	E	603	-	-	0/5/5/5	-
5	MPD	C	603	-	-	0/5/5/5	-
5	MPD	H	1001	-	-	0/5/5/5	-
5	MPD	A	602	-	-	0/5/5/5	-

There are no bond length outliers.

There are no bond angle outliers.

There are no chirality outliers.

All (2) torsion outliers are listed below:

Mol	Chain	Res	Type	Atoms
5	G	603	MPD	O2-C2-C3-C4
5	G	602	MPD	C2-C3-C4-O4

There are no ring outliers.

6 monomers are involved in 7 short contacts:

For Manuscript Review

Mol	Chain	Res	Type	Clashes	Symm-Clashes
5	G	602	MPD	1	0
5	E	602	MPD	1	0
5	B	1001	MPD	1	0
5	D	602	MPD	2	0
5	H	1001	MPD	1	0
5	A	602	MPD	1	0

The following is a two-dimensional graphical depiction of Mogul quality analysis of bond lengths, bond angles, torsion angles, and ring geometry for all instances of the Ligand of Interest. In addition, ligands with molecular weight > 250 and outliers as shown on the validation Tables will also be included. For torsion angles, if less then 5% of the Mogul distribution of torsion angles is within 10 degrees of the torsion angle in question, then that torsion angle is considered an outlier. Any bond that is central to one or more torsion angles identified as an outlier by Mogul will be highlighted in the graph. For rings, the root-mean-square deviation (RMSD) between the ring in question and similar rings identified by Mogul is calculated over all ring torsion angles. If the average RMSD is greater than 60 degrees and the minimal RMSD between the ring in question and any Mogul-identified rings is also greater than 60 degrees, then that ring is considered an outlier. The outliers are highlighted in purple. The color gray indicates Mogul did not find sufficient equivalents in the CSD to analyse the geometry.

Ligand MPD C 602

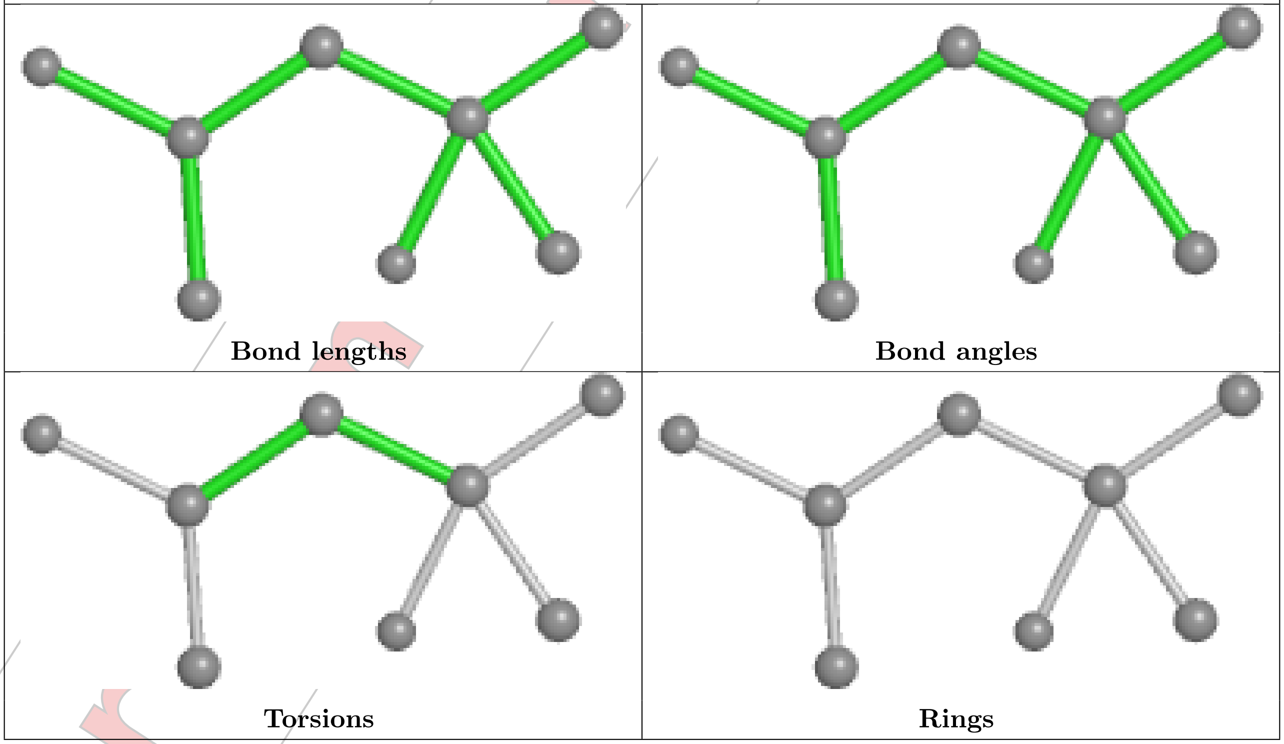

For Manuscript Review

Ligand MPD G 602

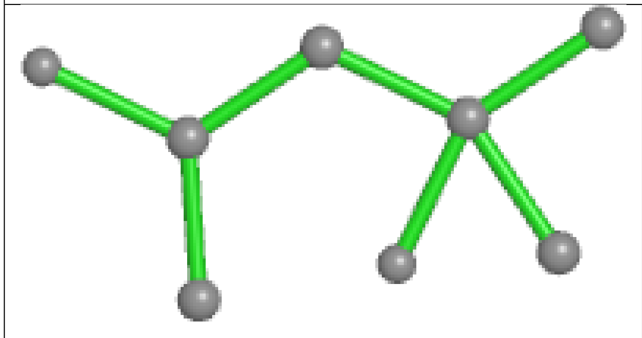
Bond lengths

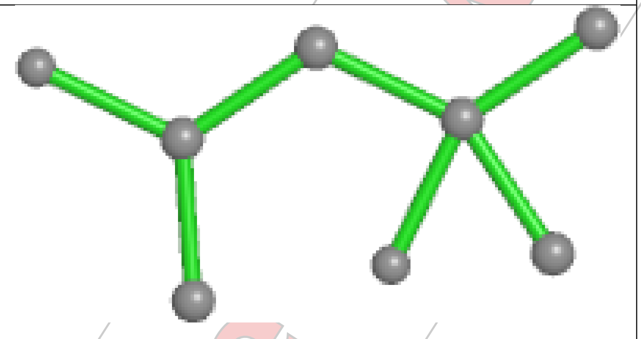
Bond angles

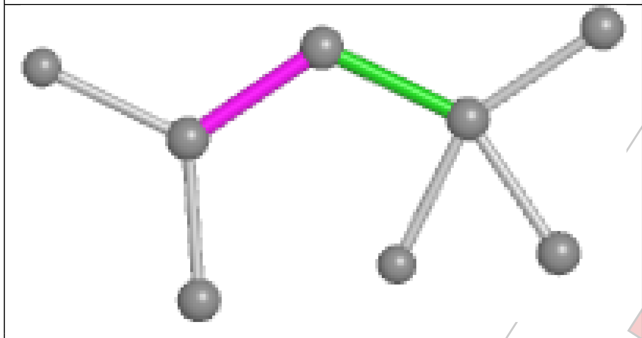
Torsions

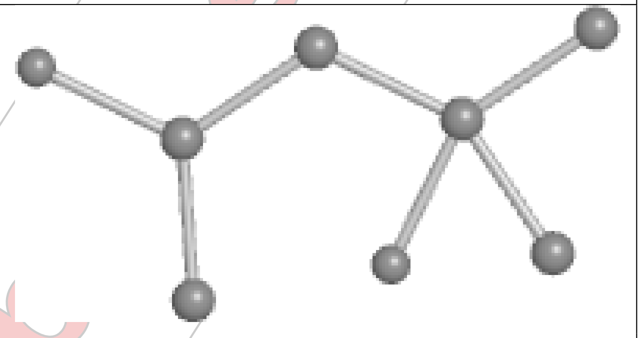
Rings

Ligand MPD A 604

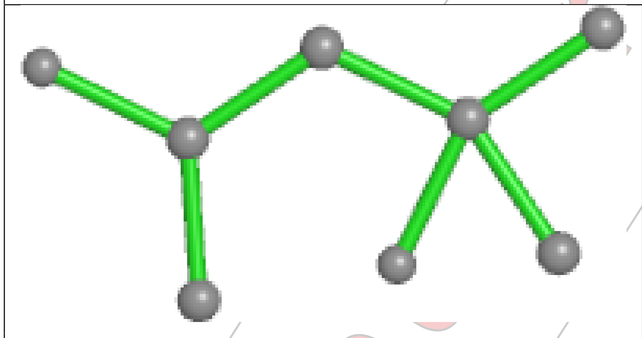
Bond lengths

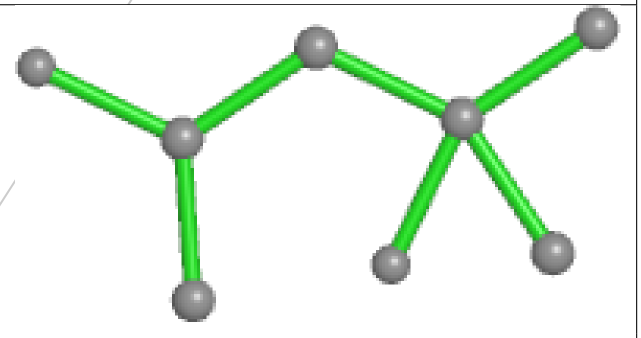
Bond angles

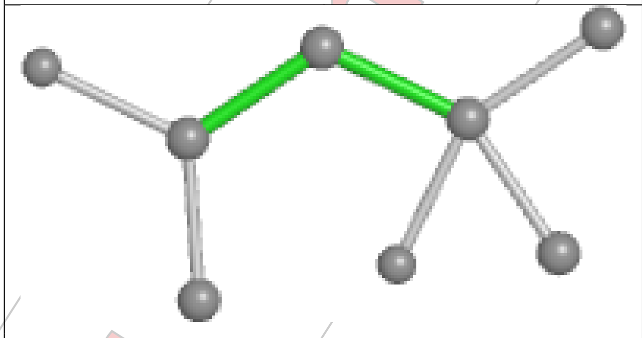
Torsions

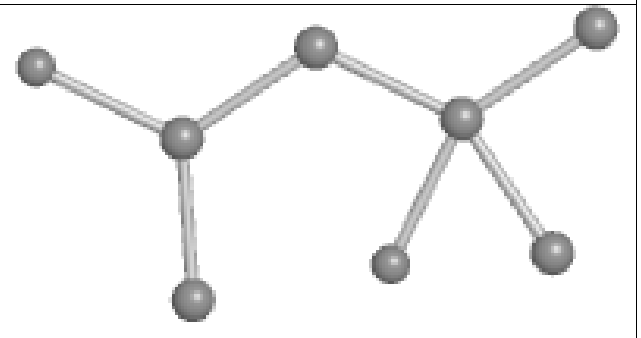
Rings

Ligand MPD F 1002

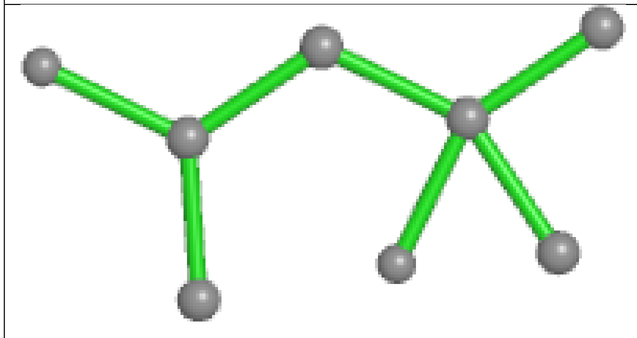
Bond lengths

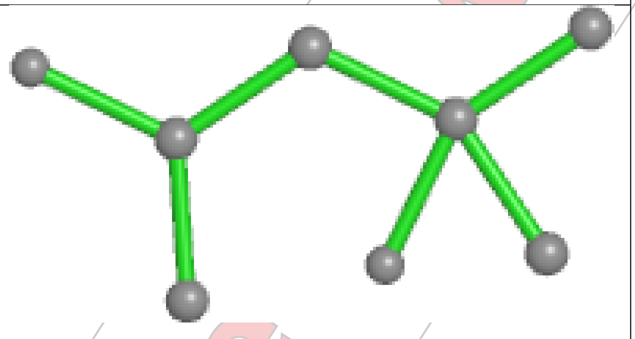
Bond angles

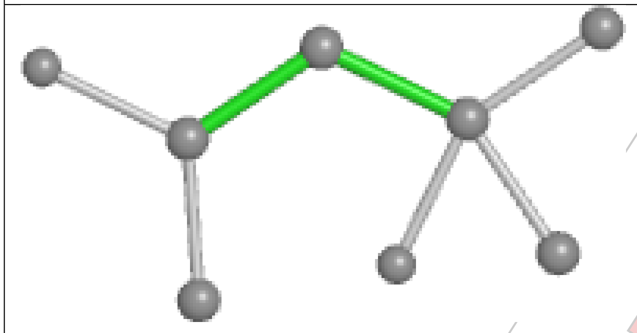
Torsions

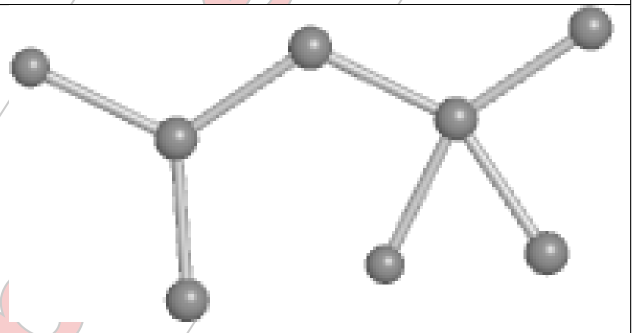
Rings

Ligand MPD E 602

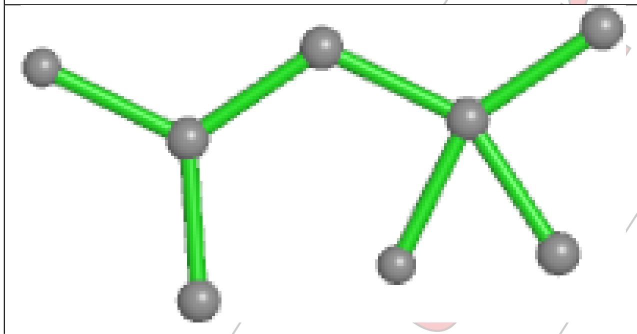
Bond lengths

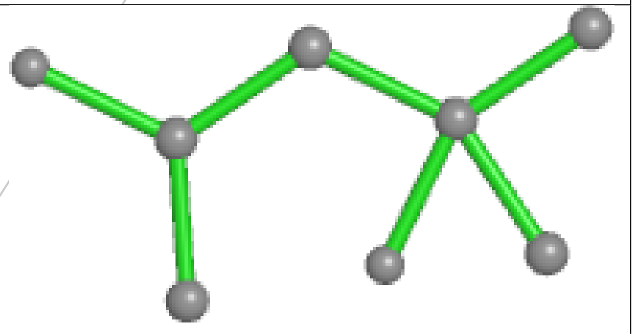
Bond angles

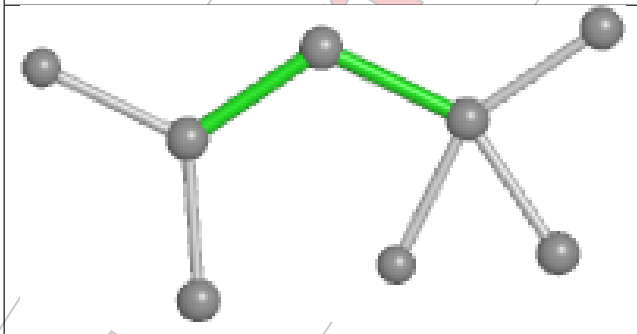
Torsions

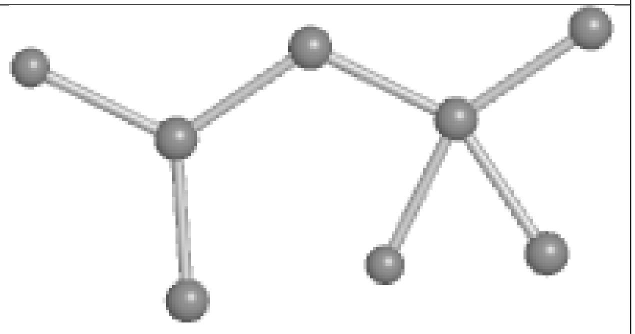
Rings

Ligand MPD B 1001

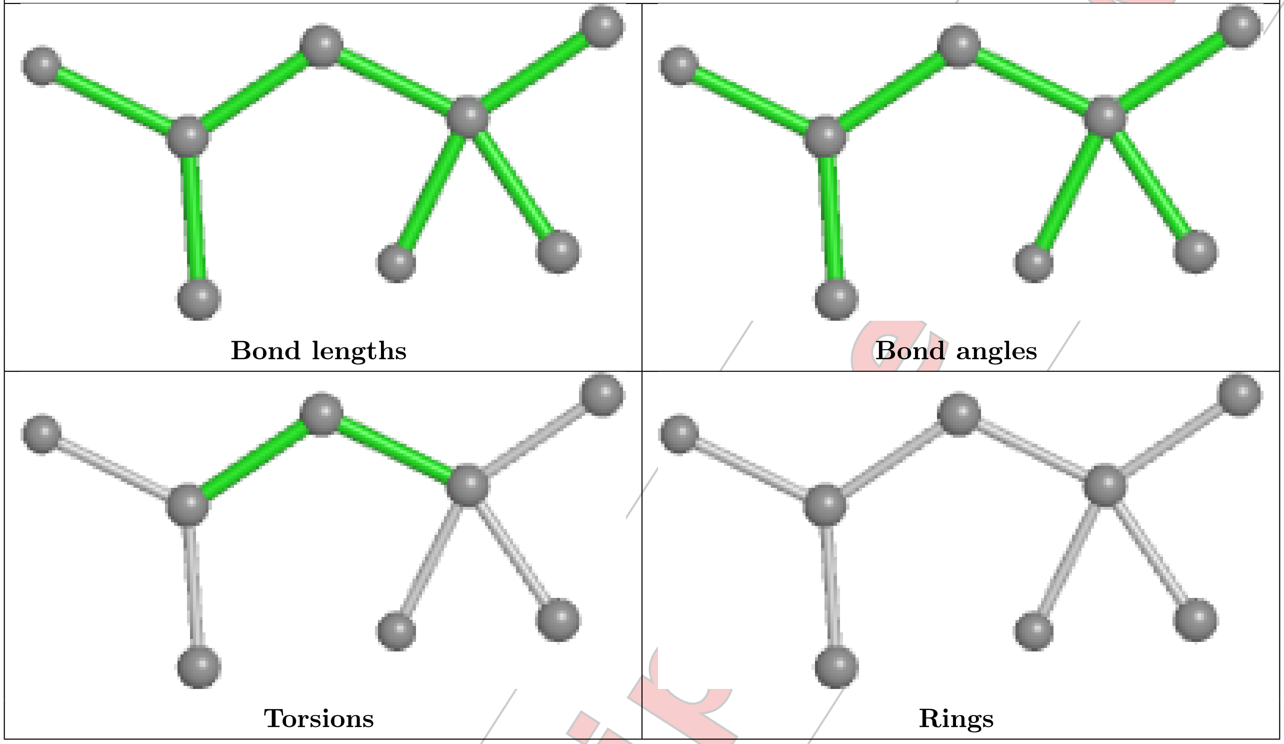

Ligand MPD F 1001

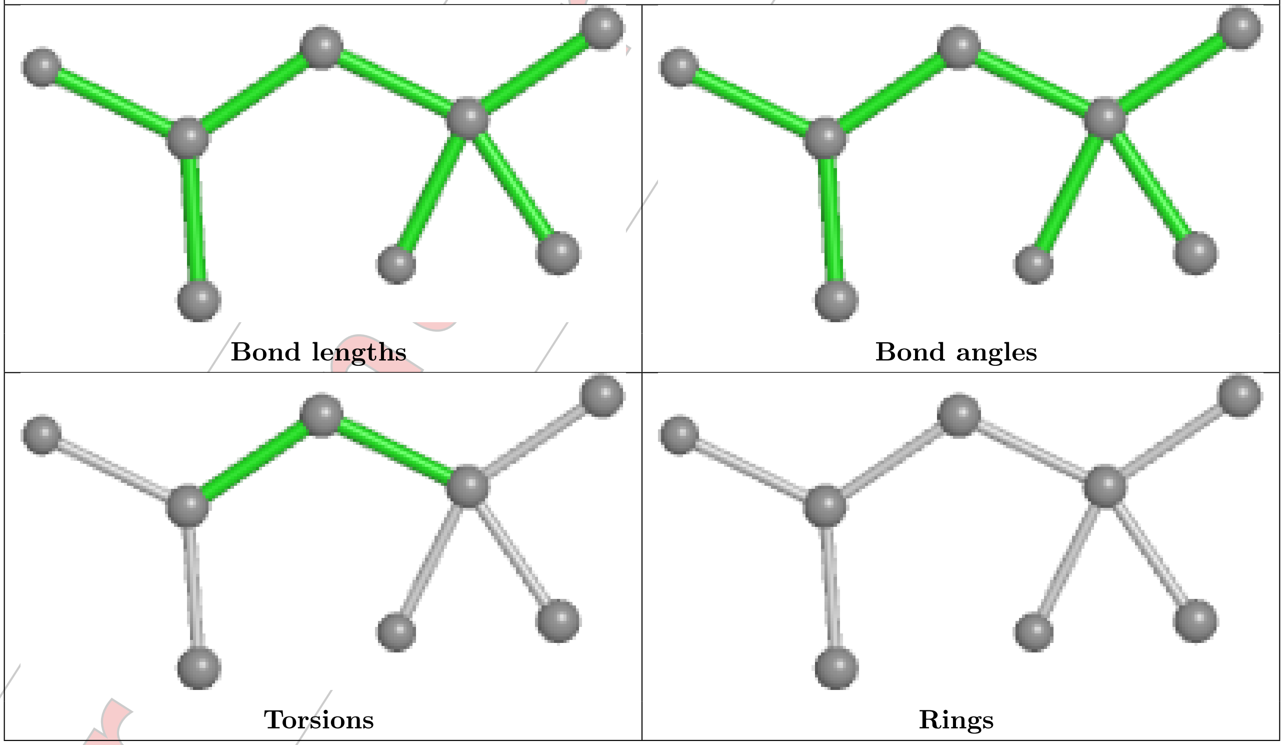

Ligand MPD G 603

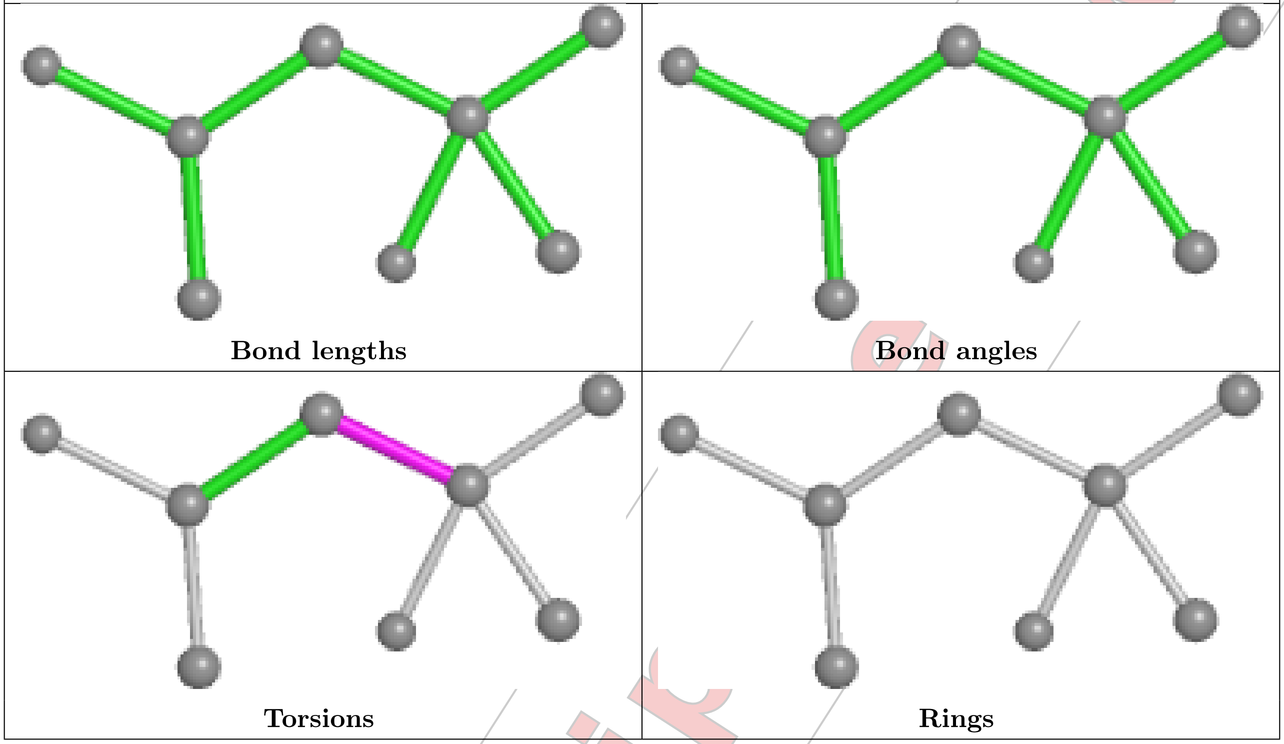

Ligand MPD D 603

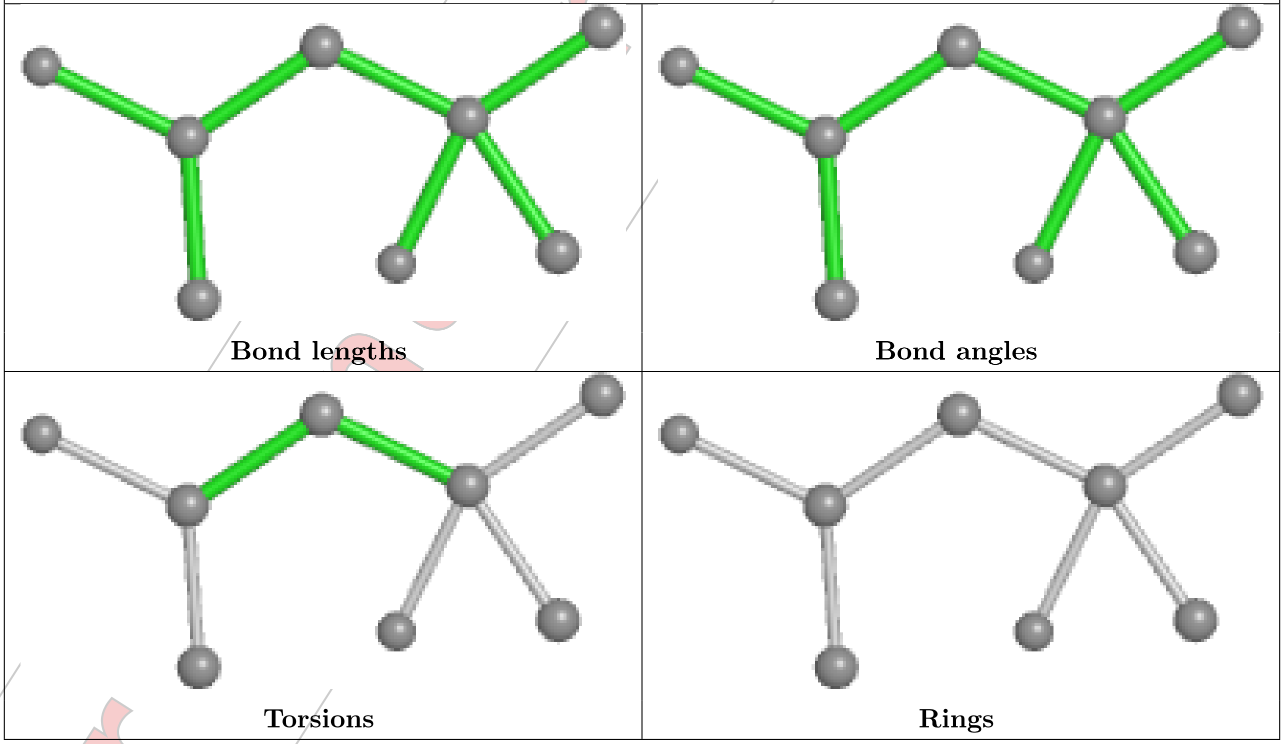

Ligand MPD D 602

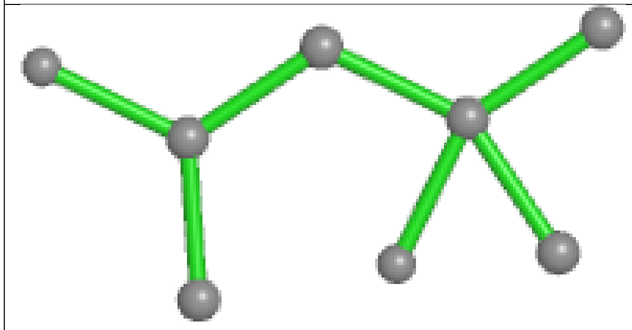

Bond lengths

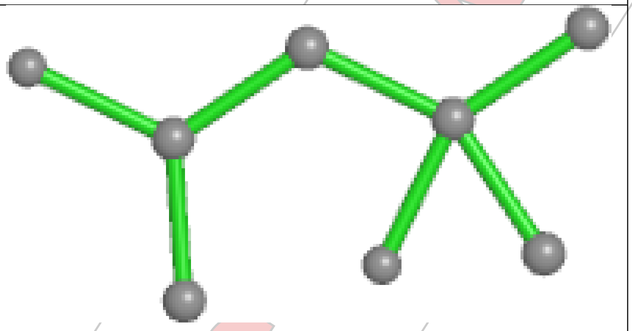

Bond angles

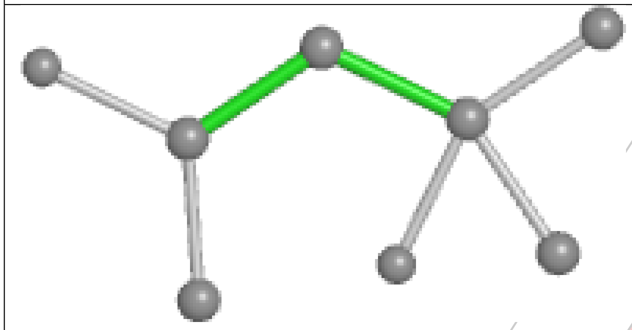

Torsions

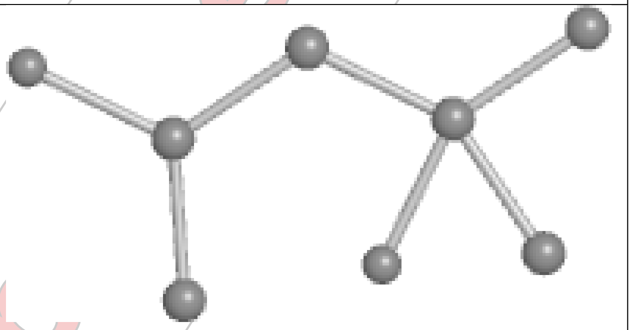

Rings

Ligand MPD E 604

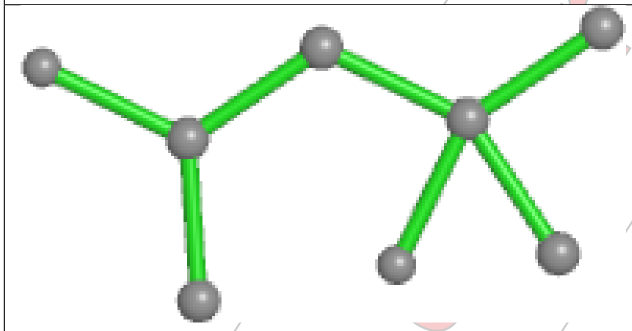

Bond lengths

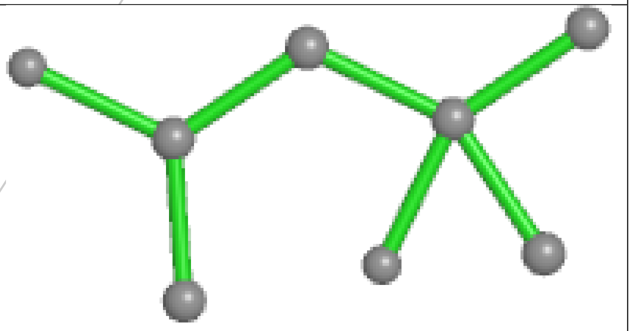

Bond angles

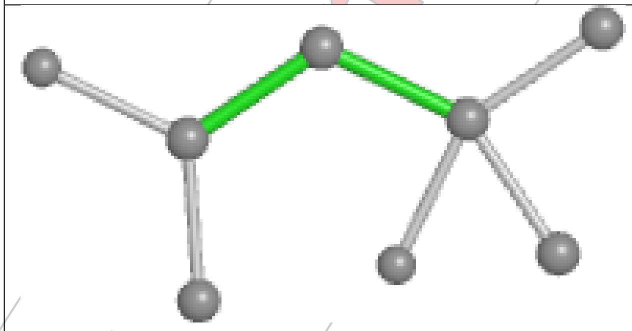

Torsions

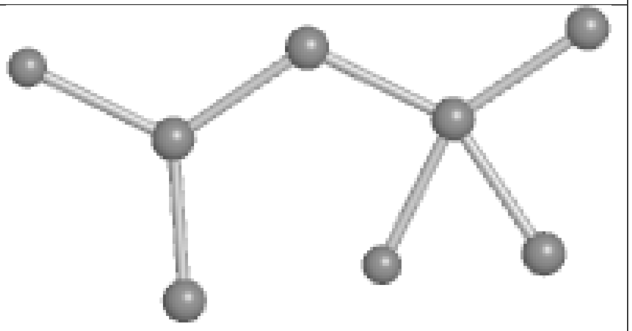

Rings

Ligand MPD E 603

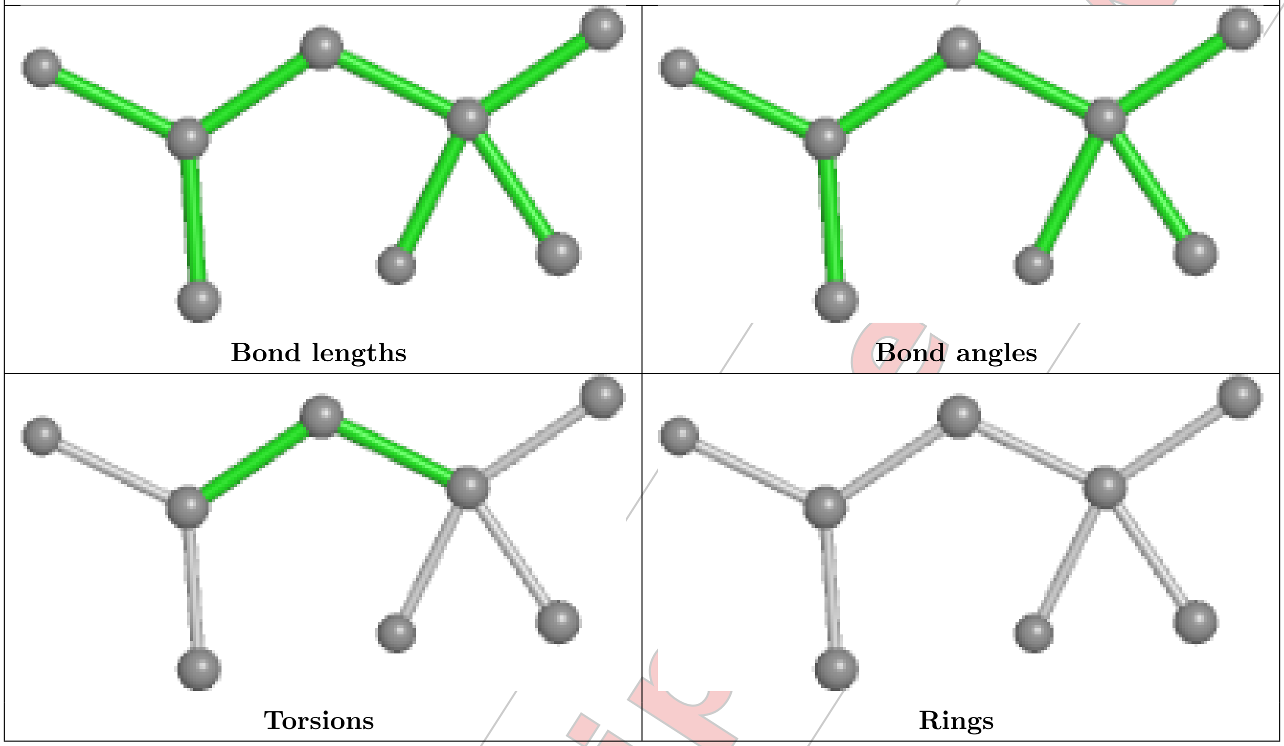

Ligand MPD C 603

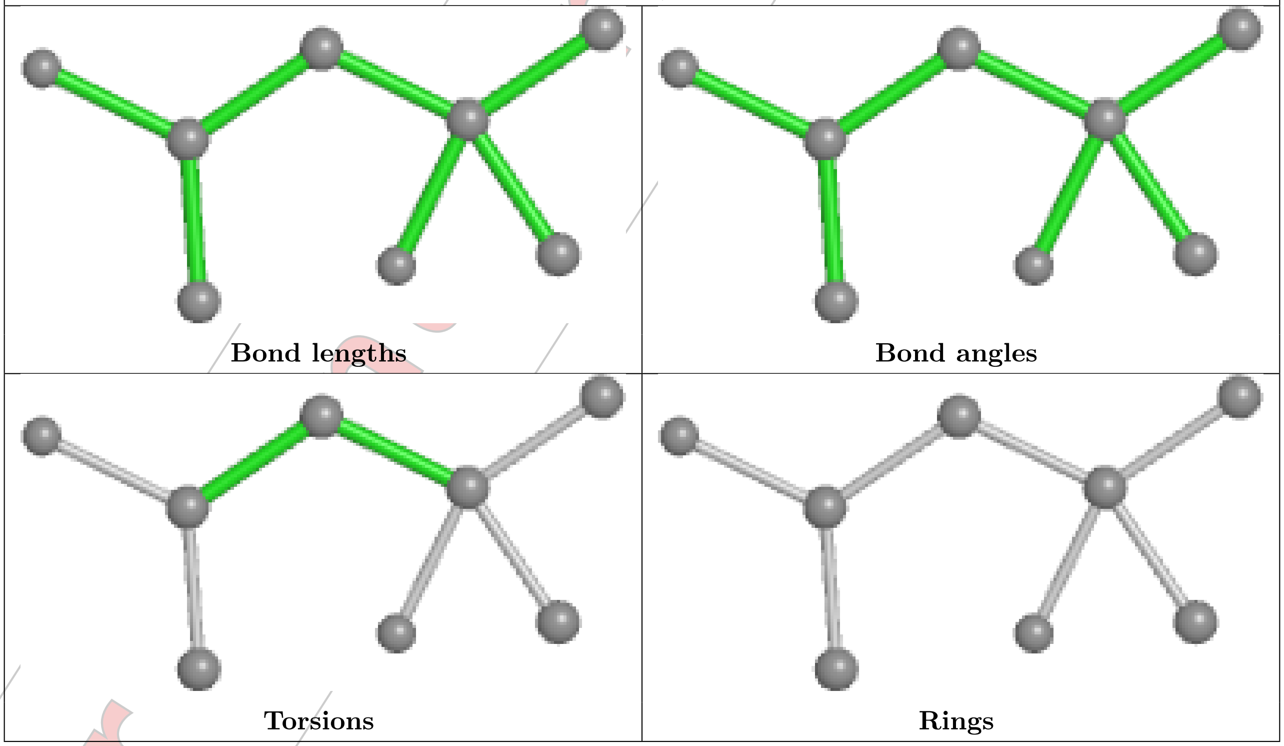

Ligand MPD H 1001

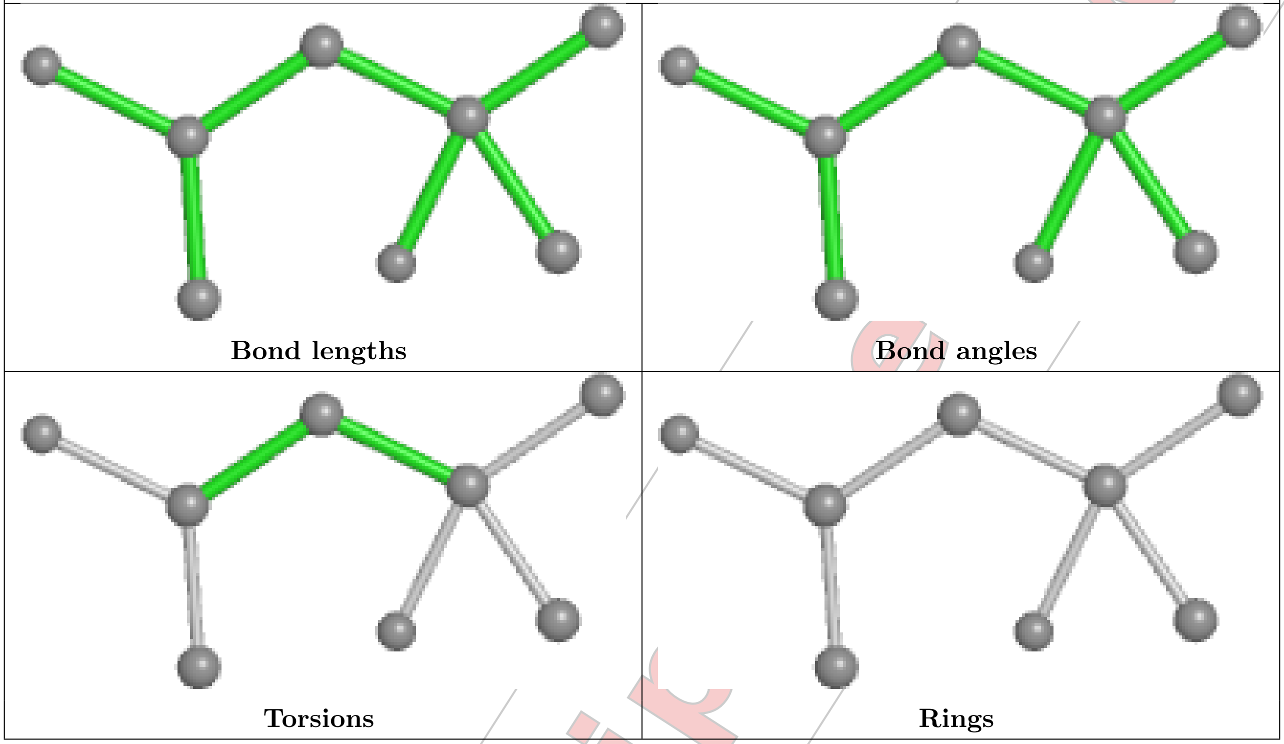

Ligand MPD A 602

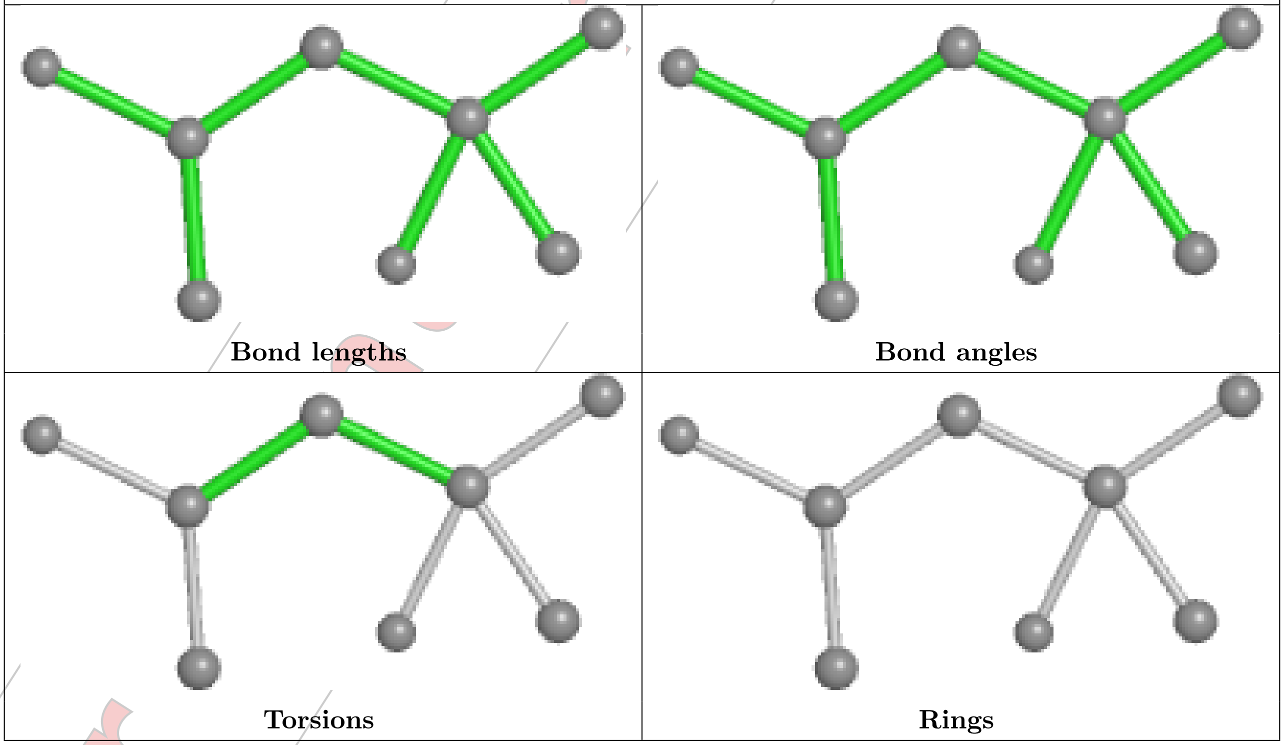

5.7 Other polymers (i)

There are no such residues in this entry.

5.8 Polymer linkage issues (i)

There are no chain breaks in this entry.

For Manuscript Review

6 Fit of model and data (i)

6.1 Protein, DNA and RNA chains (i)

In the following table, the column labelled '#RSRZ> 2' contains the number (and percentage) of RSRZ outliers, followed by percent RSRZ outliers for the chain as percentile scores relative to all X-ray entries and entries of similar resolution. The OWAB column contains the minimum, median, 95th percentile and maximum values of the occupancy-weighted average B-factor per residue. The column labelled 'Q< 0.9' lists the number of (and percentage) of residues with an average occupancy less than 0.9.

Mol	Chain	Analysed	<RSRZ>	#RSRZ>2			OWAB(Å^2)	Q<0.9
1	A	483/509 (94%)	0.24	19 (3%)	39	49	26, 39, 59, 78	0
1	B	506/509 (99%)	0.29	27 (5%)	26	35	22, 33, 66, 126	0
1	C	482/509 (94%)	0.20	18 (3%)	41	51	23, 36, 58, 80	0
1	D	497/509 (97%)	0.26	22 (4%)	34	44	23, 37, 59, 118	0
2	E	493/509 (96%)	0.03	9 (1%)	68	76	23, 32, 49, 77	0
2	F	500/509 (98%)	-0.10	8 (1%)	72	79	21, 31, 48, 71	0
3	G	489/509 (96%)	0.46	33 (6%)	17	26	22, 40, 63, 87	0
3	H	501/509 (98%)	0.47	44 (8%)	10	16	27, 40, 66, 83	0
All	All	3951/4072 (97%)	0.23	180 (4%)	32	42	21, 36, 60, 126	0

All (180) RSRZ outliers are listed below:

Mol	Chain	Res	Type	RSRZ
1	B	118	ILE	14.7
1	B	115	CYS	11.8
1	D	118	ILE	9.9
1	B	119	PHE	9.8
1	B	120	ASP	8.6
1	D	448	PRO	7.8
1	B	125	PHE	7.7
1	B	117	THR	7.5
3	G	270	ILE	7.1
1	B	116	HIS	7.0
1	B	4	THR	6.9
1	D	119	PHE	6.4
3	H	205	ILE	6.2
3	H	57	THR	6.1
1	D	217	LYS	6.0
1	B	113	ASP	6.0

Continued on next page...

For Manuscript Review

Continued from previous page...

Mol	Chain	Res	Type	RSRZ
1	D	115	CYS	5.9
2	E	118	ILE	5.7
3	H	40	TRP	5.5
1	B	122	VAL	5.4
3	G	57	THR	5.3
1	D	125	PHE	5.2
1	D	122	VAL	5.1
1	B	114	PRO	5.1
1	D	121	ARG	5.1
3	G	266	MET	5.0
1	C	57	THR	5.0
1	B	124	ALA	4.9
1	B	123	ALA	4.9
3	G	263	TYR	4.8
1	C	263	TYR	4.8
3	H	125	PHE	4.7
3	H	508	ASN	4.7
1	B	127	ASN	4.7
3	G	215	THR	4.6
2	F	126	PHE	4.6
3	H	119	PHE	4.6
1	D	113	ASP	4.5
3	H	55	VAL	4.4
1	A	270	ILE	4.3
3	G	262	VAL	4.3
3	H	126	PHE	4.2
3	G	449	ASN	4.2
1	D	114	PRO	4.2
1	B	131	HIS	4.1
3	H	56	GLY	4.1
3	H	116	HIS	4.1
1	A	132	MET	4.1
3	G	131	HIS	4.1
1	B	126	PHE	4.0
3	H	17[A]	TRP	4.0
1	D	449	ASN	3.9
1	D	116	HIS	3.9
1	D	117	THR	3.9
2	E	117	THR	3.8
1	B	3	ALA	3.8
3	H	39	SER	3.8
2	F	128	PRO	3.8

Continued on next page...

For Manuscript Review

Continued from previous page...

Mol	Chain	Res	Type	RSRZ
3	H	97	ALA	3.7
3	G	428	GLU	3.7
1	D	276	ALA	3.7
3	G	448	PRO	3.7
3	H	127	ASN	3.6
3	G	265	LEU	3.6
3	G	116	HIS	3.6
1	C	96	ARG	3.5
3	H	99	ASN	3.5
1	D	96	ARG	3.5
3	H	109	LYS	3.5
2	E	122	VAL	3.5
1	C	276	ALA	3.4
1	D	120	ASP	3.4
2	F	124	ALA	3.4
1	B	57	THR	3.4
3	H	112	PRO	3.4
3	H	31	GLY	3.4
1	C	270	ILE	3.3
2	F	263	TYR	3.3
3	G	36	GLU	3.3
2	E	116	HIS	3.2
3	G	269	LEU	3.2
1	B	112	PRO	3.2
3	H	120	ASP	3.2
1	C	273	ASN	3.2
1	B	2	ALA	3.2
3	G	56	GLY	3.2
3	H	449	ASN	3.2
1	B	263	TYR	3.2
1	C	375[A]	ARG	3.1
1	A	271	ASN	3.1
1	C	204	ARG	3.1
3	H	16	VAL	3.0
1	C	97	ALA	3.0
3	G	96	ARG	3.0
3	G	383	ASP	3.0
3	G	55	VAL	3.0
3	G	508	ASN	3.0
3	H	96	ARG	3.0
1	A	277	ALA	3.0
1	B	272	ASN	2.9

Continued on next page...

For Manuscript Review

Continued from previous page...

Mol	Chain	Res	Type	RSRZ
1	C	271	ASN	2.9
3	G	352	ASP	2.9
2	F	127	ASN	2.9
3	H	234	TRP	2.8
1	A	263	TYR	2.8
3	G	451	LYS	2.8
3	G	145	ASP	2.8
2	F	57	THR	2.8
3	G	356	TRP	2.8
1	A	31	GLY	2.7
1	C	217	LYS	2.7
3	H	118	ILE	2.7
1	B	96	ARG	2.7
3	G	112	PRO	2.7
1	A	217	LYS	2.7
2	E	263	TYR	2.7
1	C	272	ASN	2.7
2	E	449	ASN	2.6
3	G	272	ASN	2.6
1	D	369	ASN	2.6
1	B	111	ALA	2.6
3	G	97	ALA	2.6
1	A	285	TYR	2.5
3	G	342	HIS	2.5
3	H	204	ARG	2.5
3	H	269	LEU	2.5
2	F	116	HIS	2.5
1	D	295	THR	2.5
1	B	270	ILE	2.5
1	B	121	ARG	2.5
1	D	112	PRO	2.5
1	A	264	GLY	2.5
3	H	111	ALA	2.4
3	H	507	THR	2.4
1	A	216	GLU	2.4
3	H	345	GLU	2.4
1	C	449	ASN	2.4
1	A	179	VAL	2.4
2	E	342	HIS	2.4
1	C	451	LYS	2.4
1	A	173	THR	2.4
1	D	78	GLY	2.4

Continued on next page...

For Manuscript Review

Continued from previous page...

Mol	Chain	Res	Type	RSRZ
3	H	448	PRO	2.4
2	F	129	GLY	2.3
1	C	265	LEU	2.3
1	A	203	GLY	2.3
3	H	218	GLY	2.3
1	A	204	ARG	2.3
3	G	427	GLY	2.3
3	H	357	PHE	2.2
1	A	201	VAL	2.2
3	H	104	THR	2.2
3	G	395	LEU	2.2
3	H	263	TYR	2.2
3	H	235	ALA	2.2
3	H	335	VAL	2.2
1	A	30	GLU	2.2
2	E	270	ILE	2.2
3	H	217	LYS	2.2
3	H	58	MET	2.2
2	E	57	THR	2.2
1	C	218	GLY	2.2
3	G	37	PHE	2.2
1	D	227	VAL	2.2
1	C	111	ALA	2.1
3	H	199	SER	2.1
3	H	122	VAL	2.1
1	A	109	LYS	2.1
3	H	145	ASP	2.1
1	C	56	GLY	2.1
3	G	450	ALA	2.1
3	H	117	THR	2.1
1	D	123	ALA	2.1
3	G	223	SER	2.1
1	B	448	PRO	2.1
1	A	110	SER	2.0
1	A	37	PHE	2.0
3	H	100	ARG	2.0
3	H	273	ASN	2.0
3	G	80	VAL	2.0

For Manuscript Review

6.2 Non-standard residues in protein, DNA, RNA chains (i)

In the following table, the Atoms column lists the number of modelled atoms in the group and the number defined in the chemical component dictionary. The B-factors column lists the minimum, median, 95th percentile and maximum values of B factors of atoms in the group. The column labelled 'Q< 0.9' lists the number of atoms with occupancy less than 0.9.

Mol	Type	Chain	Res	Atoms	RSCC	RSR	B-factors(Å^2)	Q<0.9
2	CSO	F	115	7/8	0.83	0.20	50,53,58,61	0
2	CSO	E	115	7/8	0.85	0.25	59,64,70,73	0
3	CSD	G	115	8/9	0.96	0.13	44,48,54,55	0
3	CSD	H	115	8/9	0.97	0.10	44,47,50,52	0

6.3 Carbohydrates (i)

There are no monosaccharides in this entry.

6.4 Ligands (i)

In the following table, the Atoms column lists the number of modelled atoms in the group and the number defined in the chemical component dictionary. The B-factors column lists the minimum, median, 95th percentile and maximum values of B factors of atoms in the group. The column labelled 'Q< 0.9' lists the number of atoms with occupancy less than 0.9.

Mol	Type	Chain	Res	Atoms	RSCC	RSR	B-factors(Å^2)	Q<0.9
5	MPD	A	602	8/8	0.74	0.26	50,52,53,54	0
5	MPD	C	603	8/8	0.78	0.22	48,52,53,55	0
5	MPD	E	602	8/8	0.82	0.14	42,46,48,48	0
7	NA	D	605	1/1	0.82	0.28	47,47,47,47	1
5	MPD	C	602	8/8	0.86	0.18	48,51,52,52	0
5	MPD	F	1002	8/8	0.86	0.13	41,44,45,47	0
5	MPD	A	604	8/8	0.86	0.24	45,50,58,59	0
5	MPD	D	602	8/8	0.87	0.15	51,53,54,54	0
5	MPD	G	603	8/8	0.88	0.17	61,62,63,65	0
5	MPD	H	1001	8/8	0.88	0.14	47,49,49,51	0
5	MPD	E	603	8/8	0.88	0.14	49,52,53,55	0
5	MPD	E	604	8/8	0.89	0.16	47,48,49,50	0
5	MPD	F	1001	8/8	0.89	0.15	45,47,48,49	0
5	MPD	B	1001	8/8	0.91	0.13	45,47,48,49	0
5	MPD	D	603	8/8	0.91	0.13	44,47,49,51	0
5	MPD	G	602	8/8	0.94	0.16	46,48,51,52	0
8	CAC	F	1003	5/5	0.95	0.20	71,71,75,76	0
6	CL	E	605	1/1	0.96	0.05	57,57,57,57	0

Continued on next page...

For Manuscript Review

Continued from previous page...

Mol	Type	Chain	Res	Atoms	RSCC	RSR	B-factors(Å^2)	Q<0.9
6	CL	D	604	1/1	0.98	0.04	32,32,32,32	1
4	FE2	B	1000	1/1	0.99	0.13	27,27,27,27	0
6	CL	A	603	1/1	0.99	0.03	35,35,35,35	1
4	FE2	H	1000	1/1	1.00	0.10	28,28,28,28	0
4	FE2	A	601	1/1	1.00	0.08	34,34,34,34	0
4	FE2	C	601	1/1	1.00	0.11	25,25,25,25	0
4	FE2	D	601	1/1	1.00	0.09	31,31,31,31	0
4	FE2	E	601	1/1	1.00	0.10	26,26,26,26	0
4	FE2	F	1000	1/1	1.00	0.11	23,23,23,23	0
4	FE2	G	601	1/1	1.00	0.14	24,24,24,24	0

The following is a graphical depiction of the model fit to experimental electron density of all instances of the Ligand of Interest. In addition, ligands with molecular weight > 250 and outliers as shown on the geometry validation Tables will also be included. Each fit is shown from different orientation to approximate a three-dimensional view.

Electron density around MPD A 602:

$2mF_o$-DF_c (at 0.7 rmsd) in gray
mF_o-DF_c (at 3 rmsd) in purple (negative)
and green (positive)

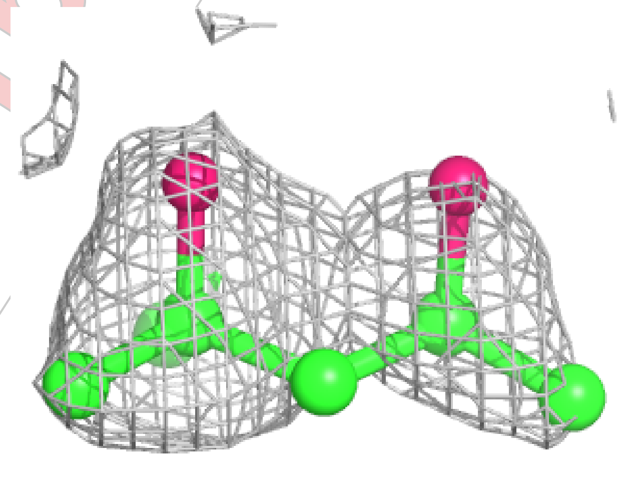

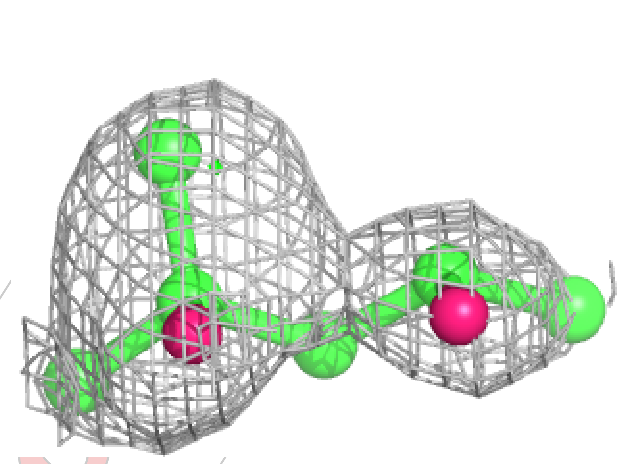

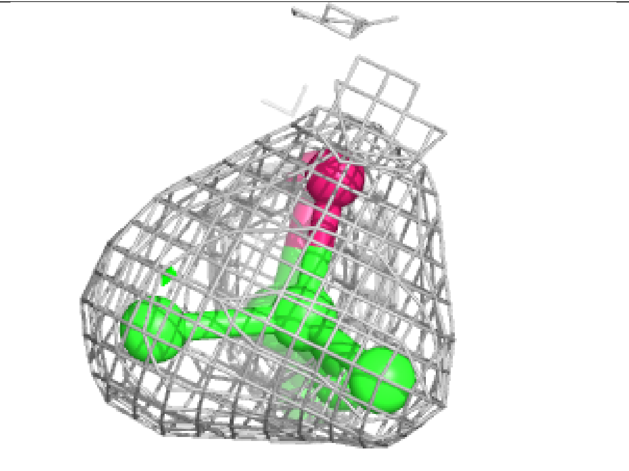

Electron density around MPD C 603:

$2mF_o$-DF_c (at 0.7 rmsd) in gray
mF_o-DF_c (at 3 rmsd) in purple (negative)
and green (positive)

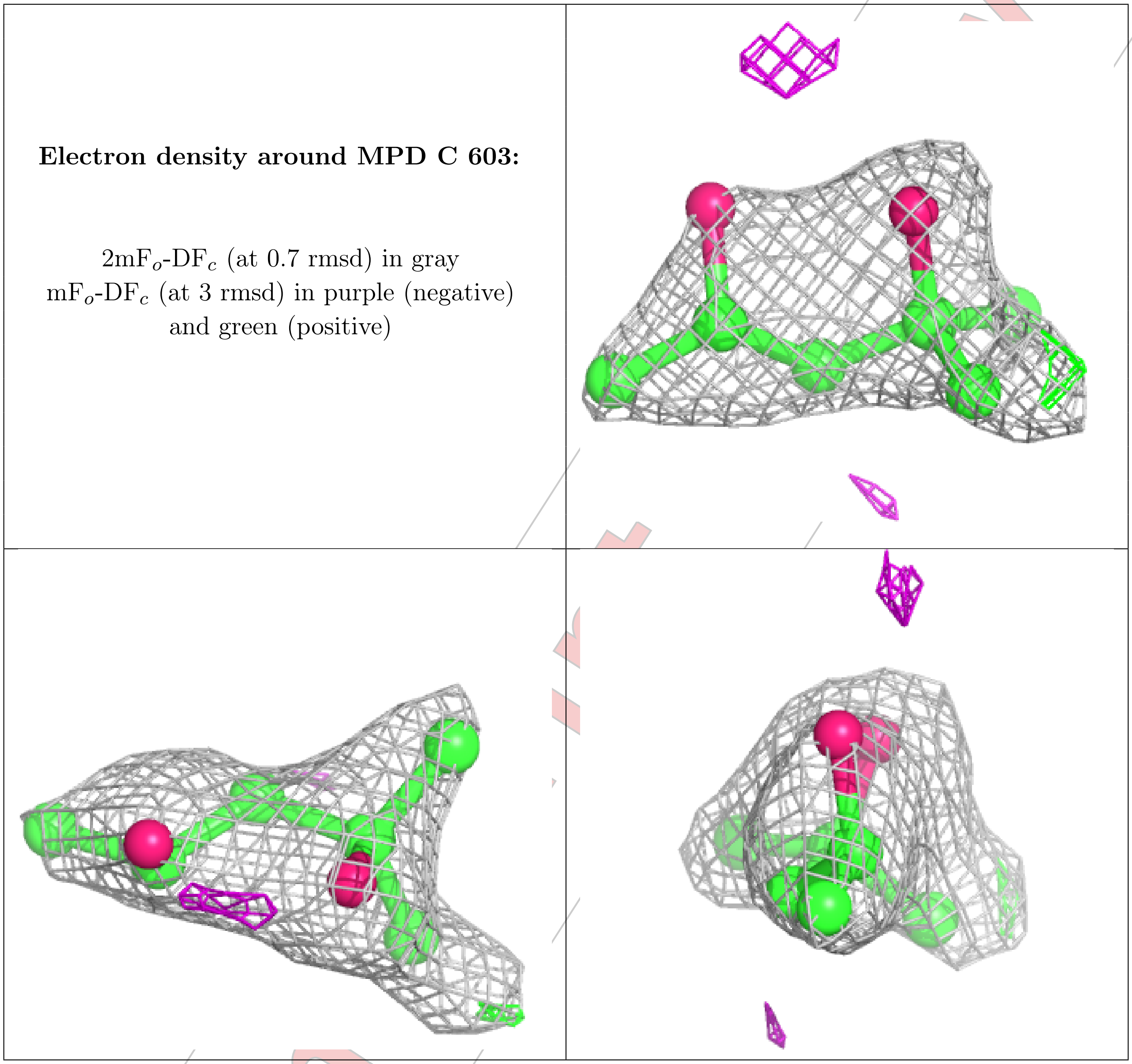

For Manuscript Review

Electron density around MPD E 602:

$2mF_o$-DF_c (at 0.7 rmsd) in gray
mF_o-DF_c (at 3 rmsd) in purple (negative)
and green (positive)

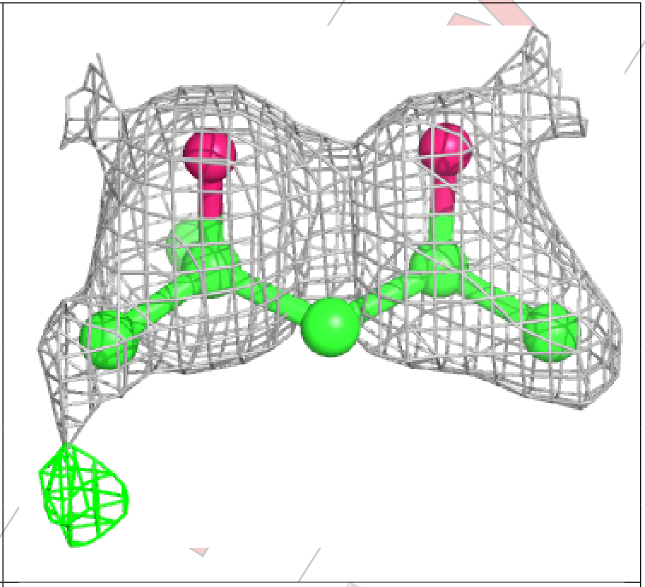

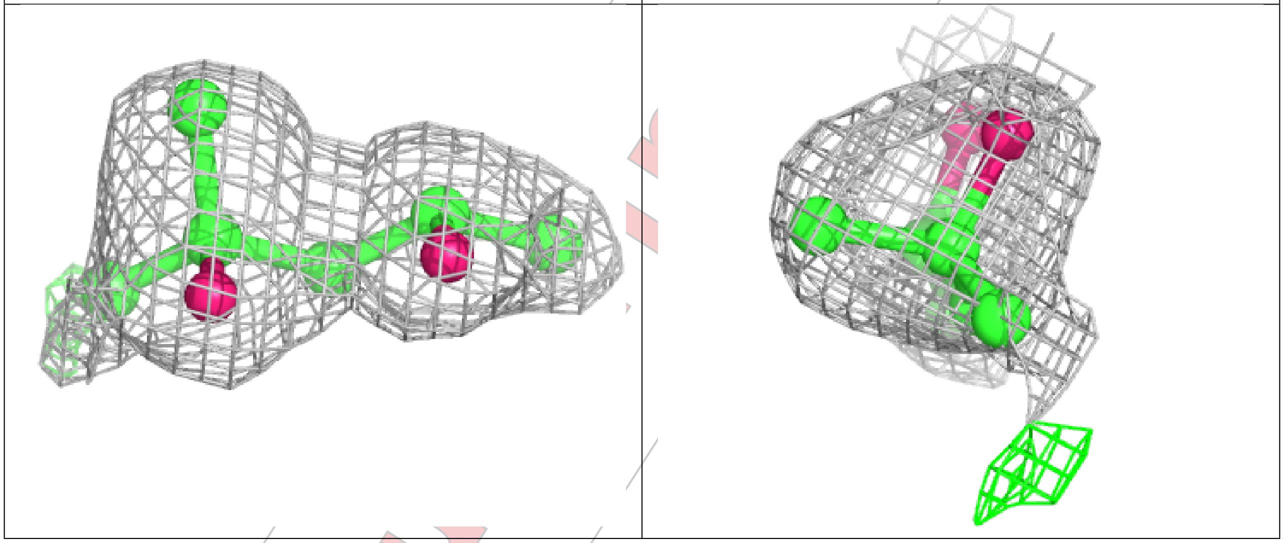

For Manuscript Review

Electron density around MPD C 602:

$2mF_o$-DF_c (at 0.7 rmsd) in gray
mF_o-DF_c (at 3 rmsd) in purple (negative)
and green (positive)

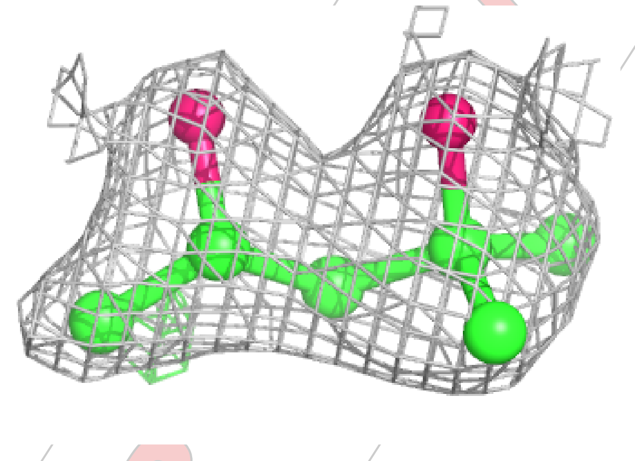

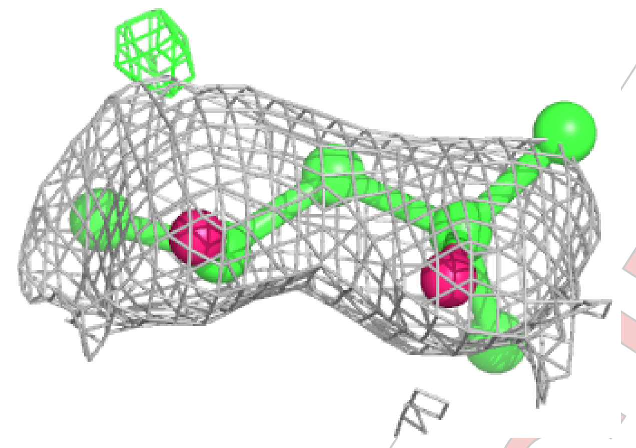

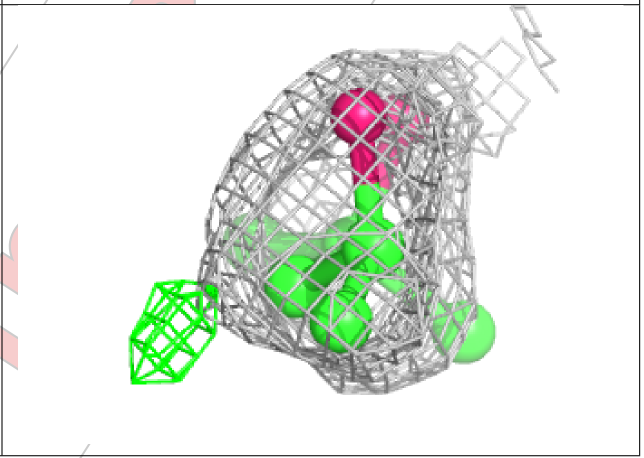

Electron density around MPD F 1002:

$2mF_o$-DF_c (at 0.7 rmsd) in gray
mF_o-DF_c (at 3 rmsd) in purple (negative)
and green (positive)

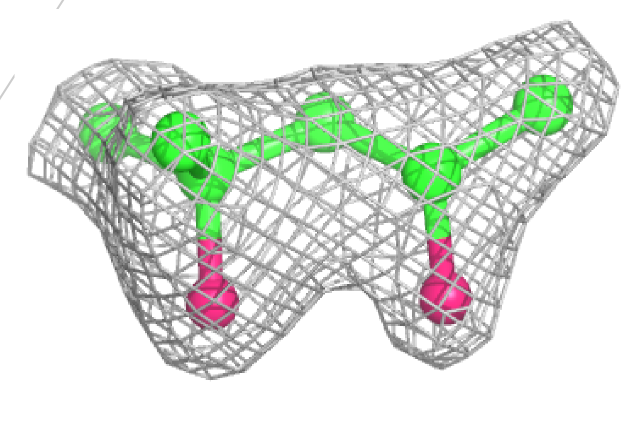

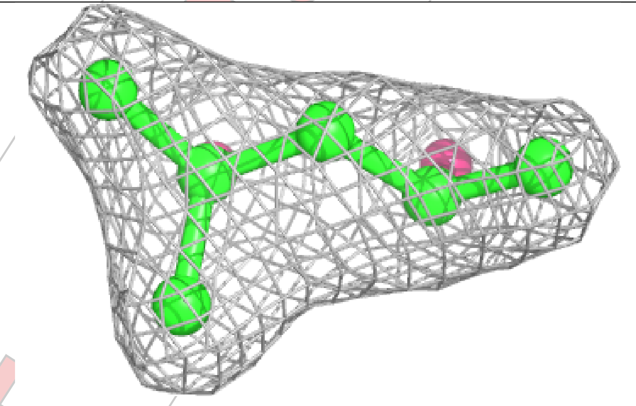

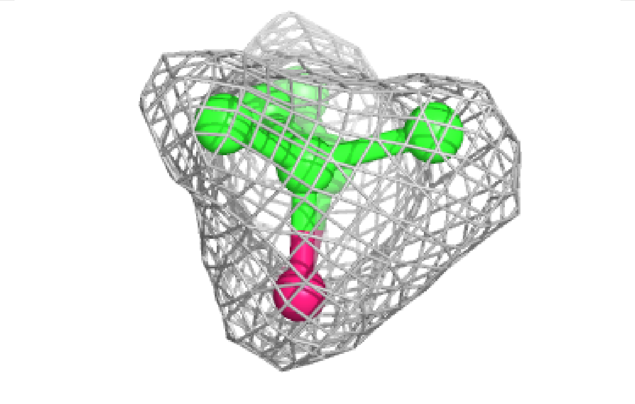

Electron density around MPD A 604:

$2mF_o$-DF_c (at 0.7 rmsd) in gray
mF_o-DF_c (at 3 rmsd) in purple (negative)
and green (positive)

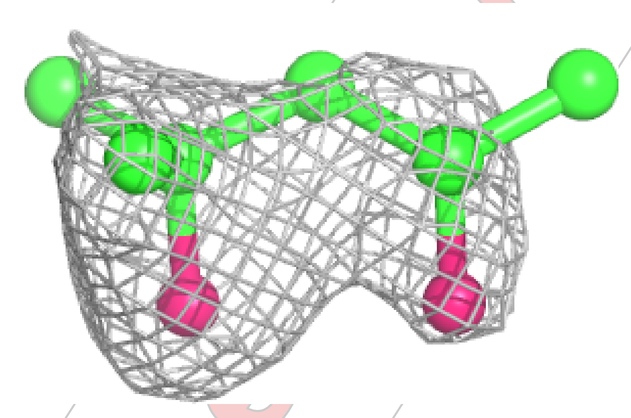

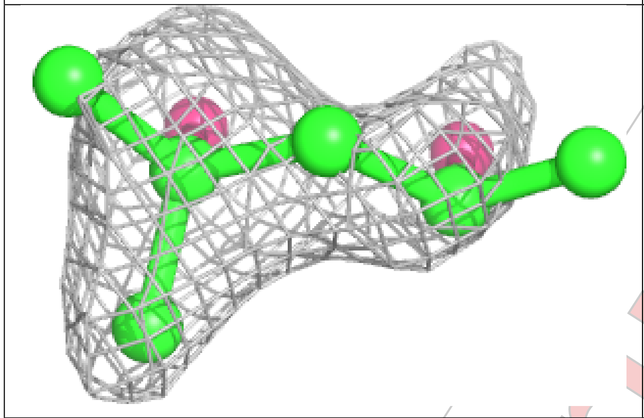

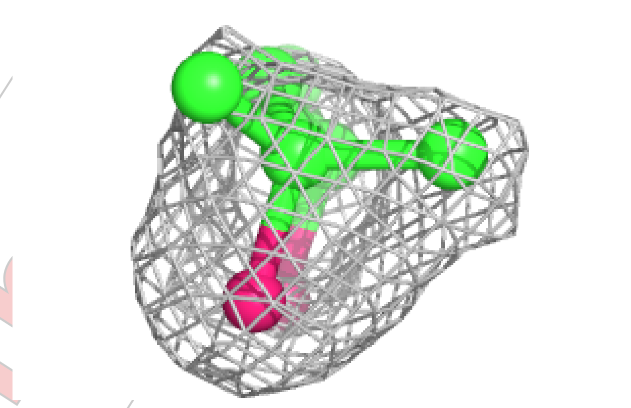

For Manuscript Review

Electron density around MPD D 602:

$2mF_o$-DF_c (at 0.7 rmsd) in gray
mF_o-DF_c (at 3 rmsd) in purple (negative)
and green (positive)

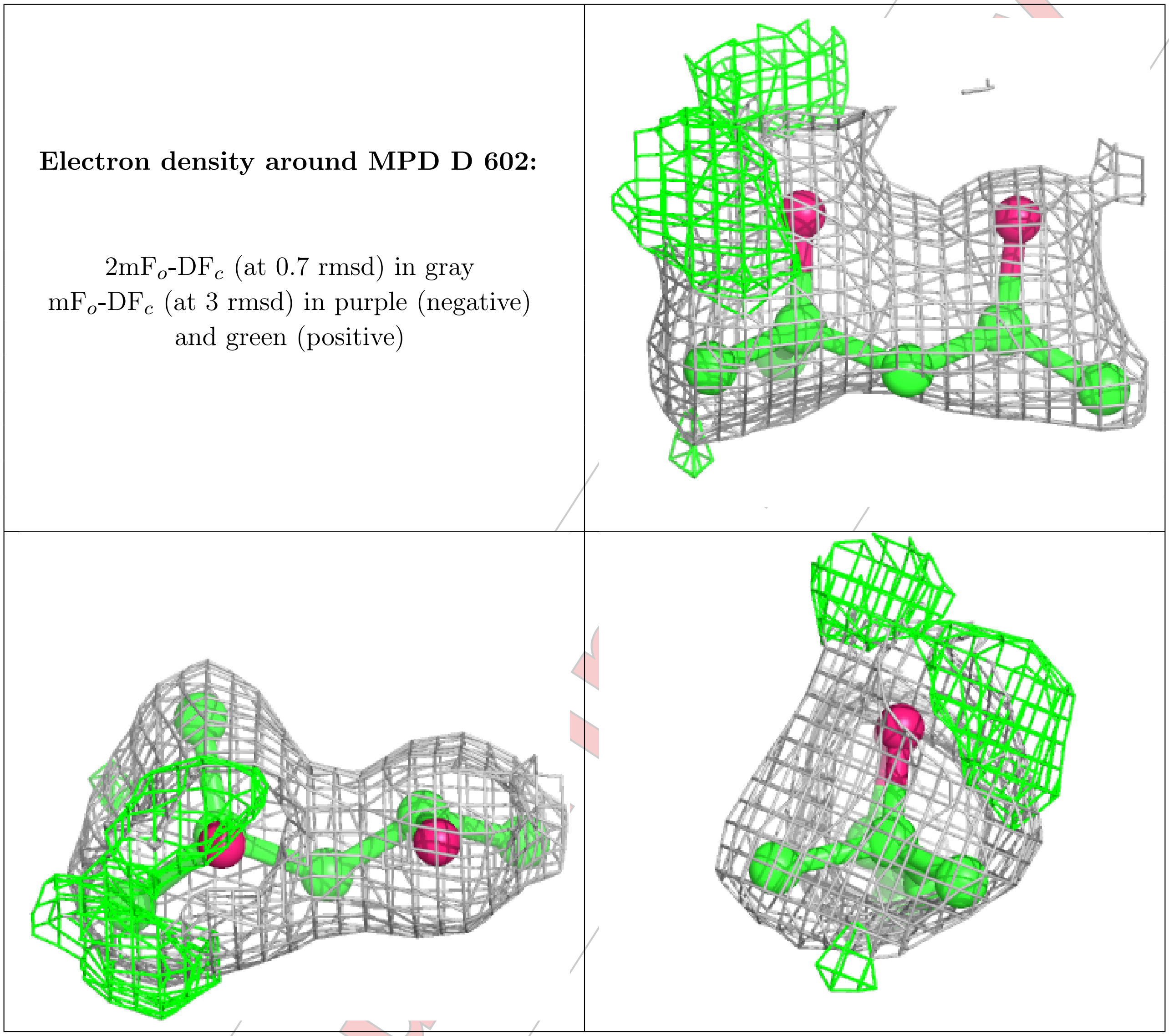

For Manuscript Review

Electron density around MPD G 603:

2mF$_o$-DF$_c$ (at 0.7 rmsd) in gray
mF$_o$-DF$_c$ (at 3 rmsd) in purple (negative)
and green (positive)

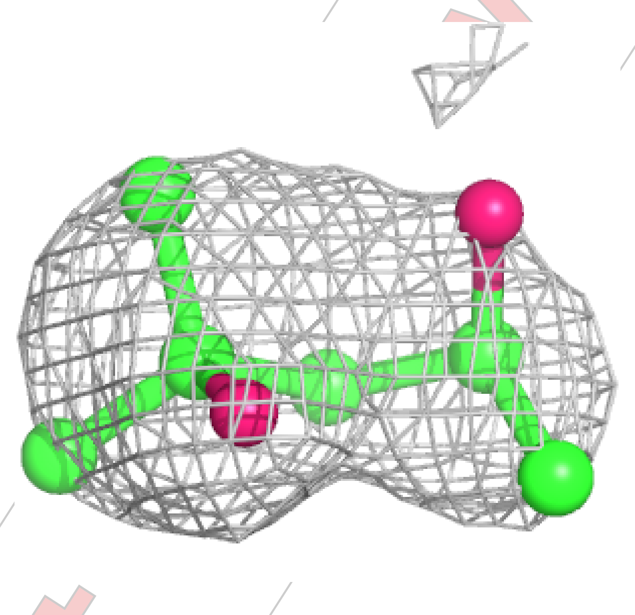

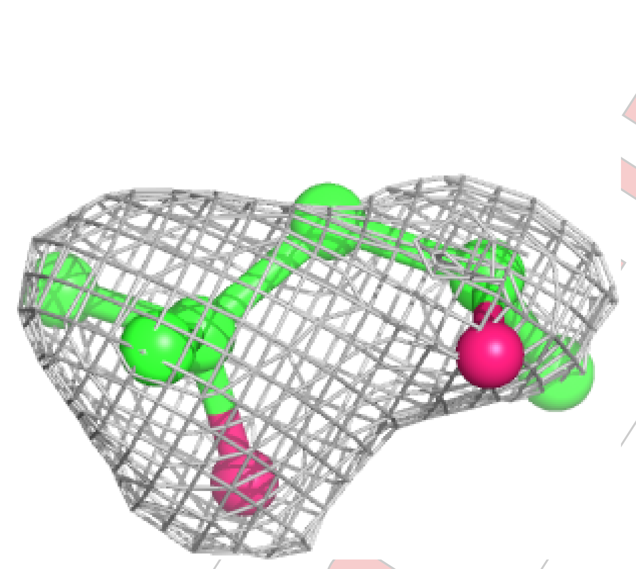

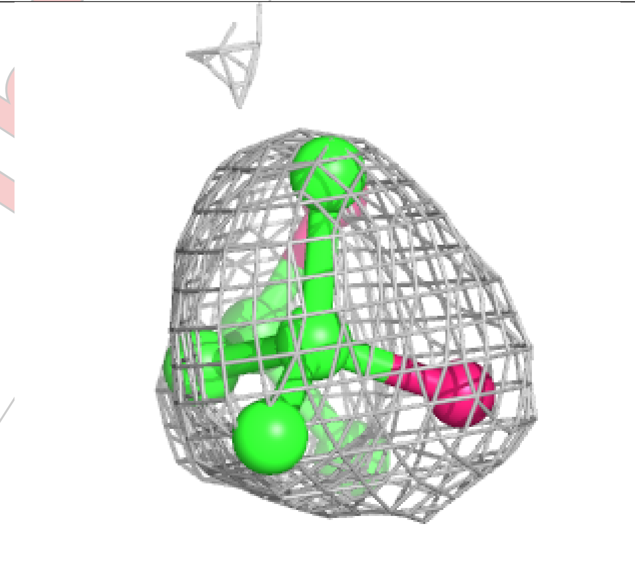

For Manuscript Review

Electron density around MPD H 1001:

2mF$_o$-DF$_c$ (at 0.7 rmsd) in gray
mF$_o$-DF$_c$ (at 3 rmsd) in purple (negative)
and green (positive)

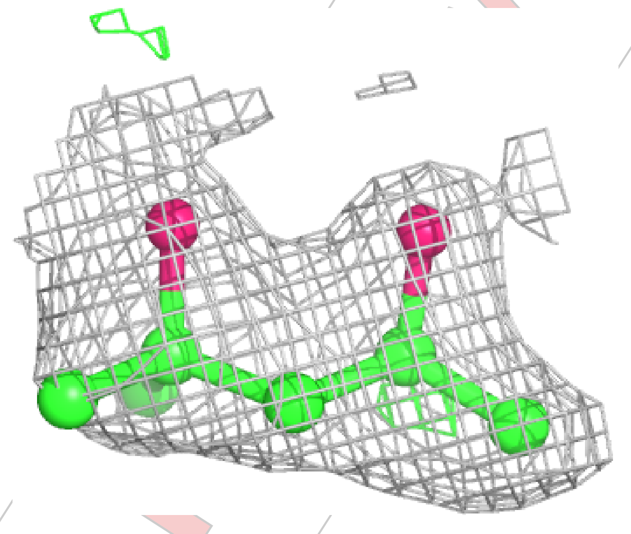

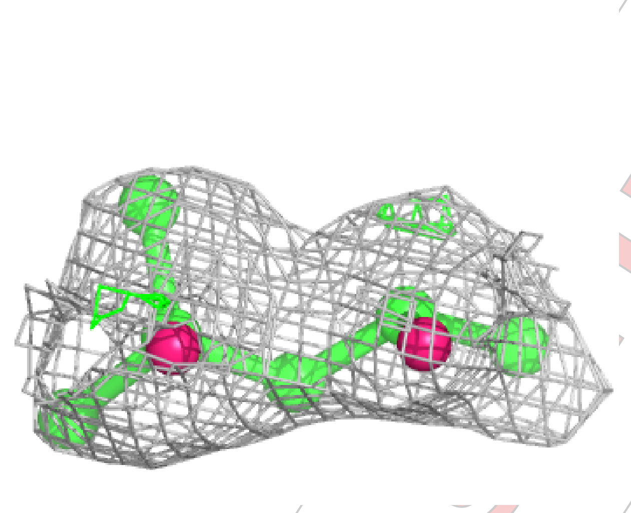

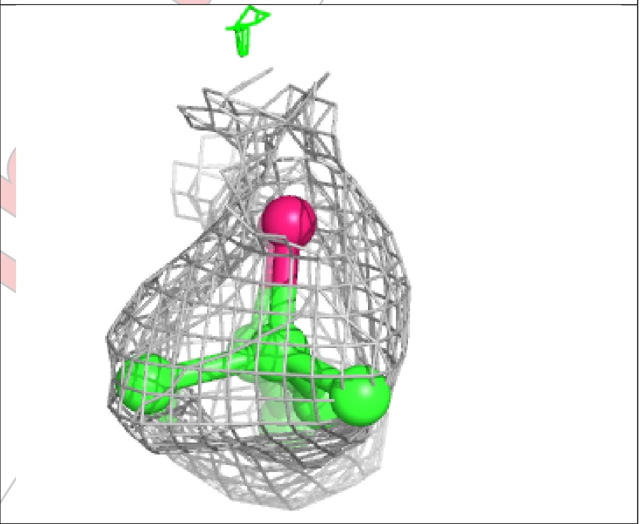

For Manuscript Review

Electron density around MPD E 603:

$2mF_o$-DF_c (at 0.7 rmsd) in gray
mF_o-DF_c (at 3 rmsd) in purple (negative)
and green (positive)

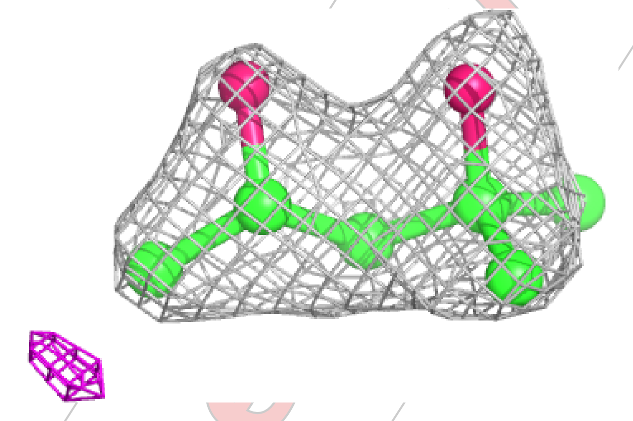

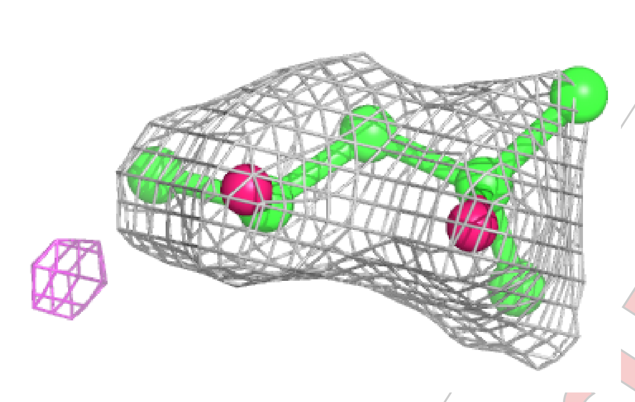

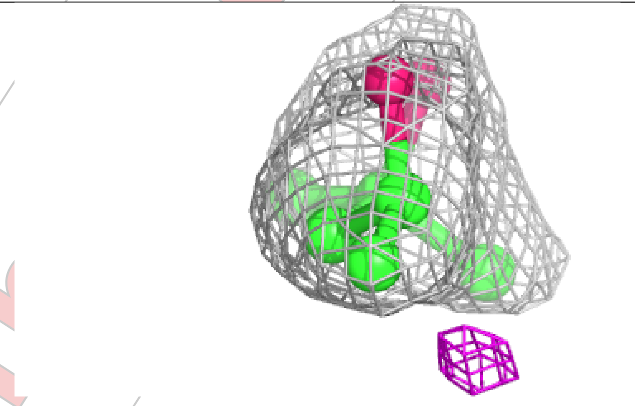

Electron density around MPD E 604:

$2mF_o$-DF_c (at 0.7 rmsd) in gray
mF_o-DF_c (at 3 rmsd) in purple (negative)
and green (positive)

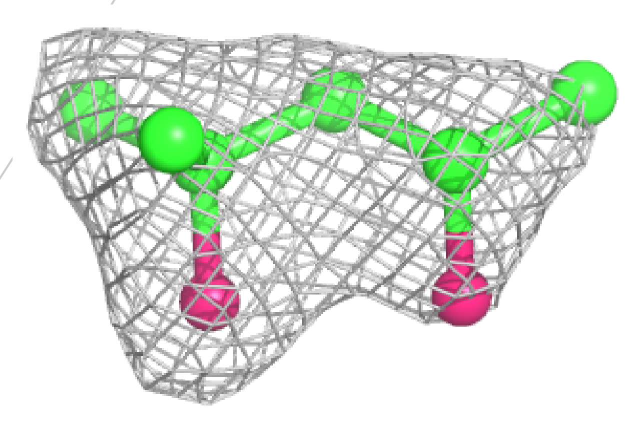

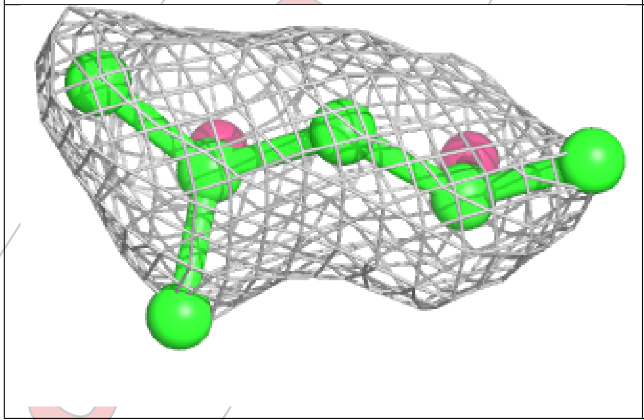

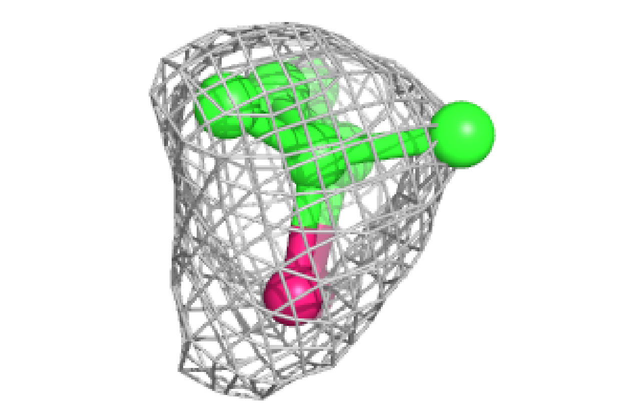

Electron density around MPD F 1001:

$2mF_o$-DF_c (at 0.7 rmsd) in gray
mF_o-DF_c (at 3 rmsd) in purple (negative)
and green (positive)

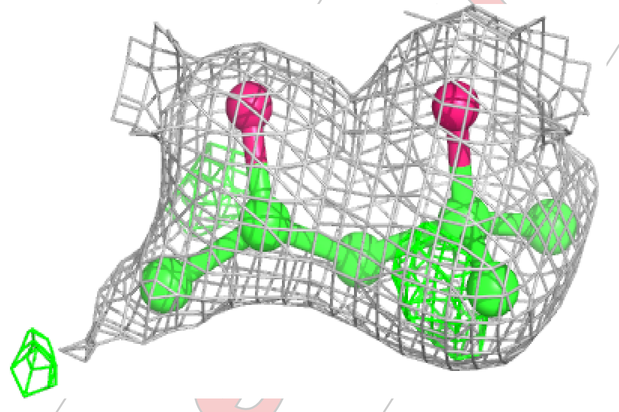

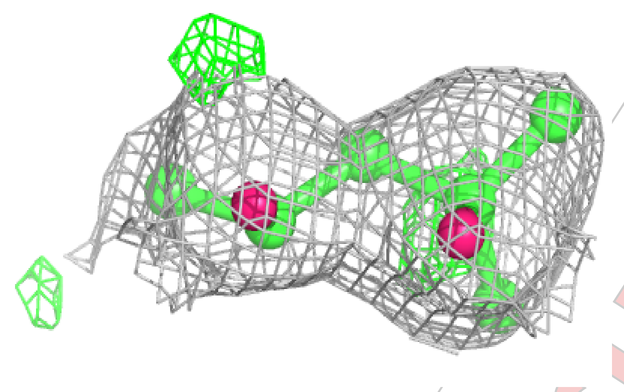

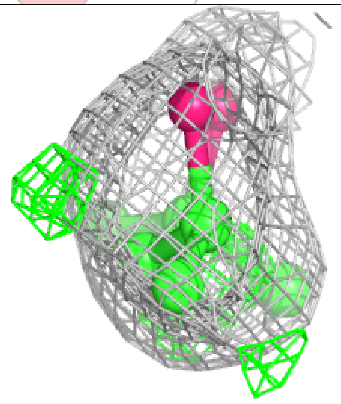

For Manuscript Review

Electron density around MPD B 1001:

$2mF_o$-DF_c (at 0.7 rmsd) in gray
mF_o-DF_c (at 3 rmsd) in purple (negative)
and green (positive)

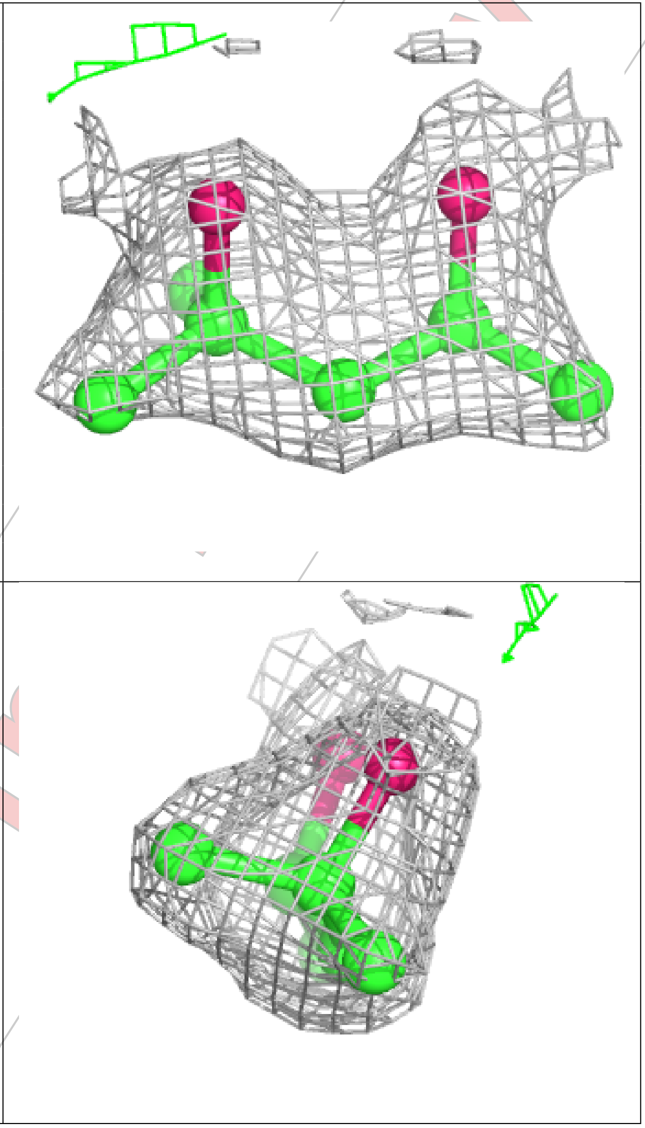

For Manuscript Review

Electron density around MPD D 603:

2mF$_o$-DF$_c$ (at 0.7 rmsd) in gray
mF$_o$-DF$_c$ (at 3 rmsd) in purple (negative)
and green (positive)

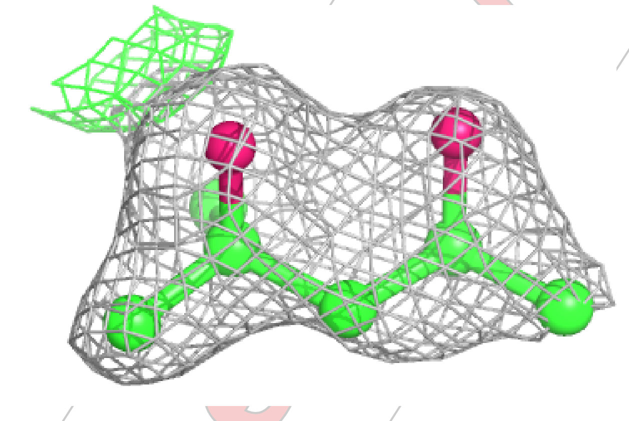

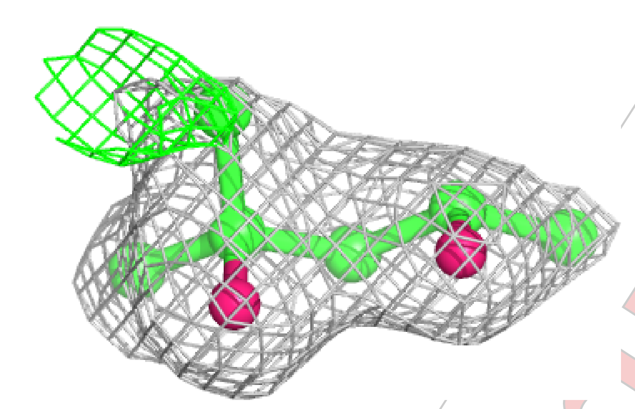

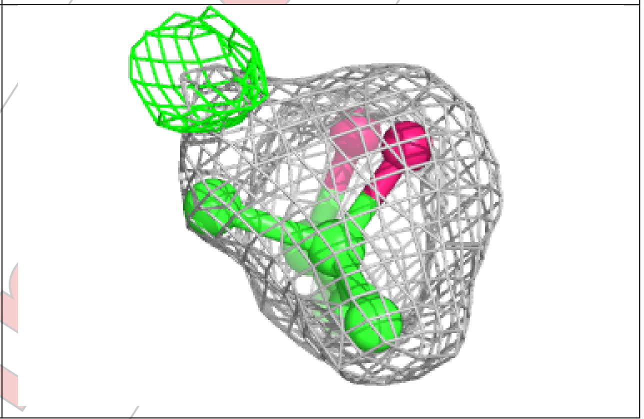

Electron density around MPD G 602:

2mF$_o$-DF$_c$ (at 0.7 rmsd) in gray
mF$_o$-DF$_c$ (at 3 rmsd) in purple (negative)
and green (positive)

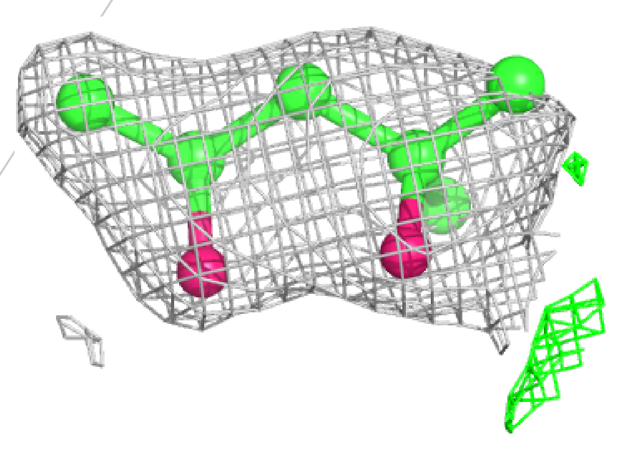

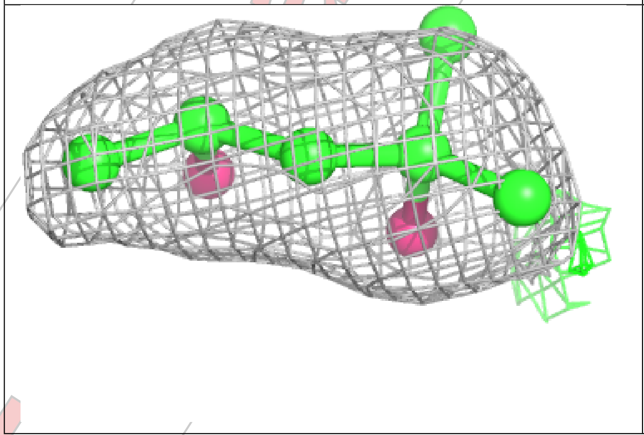

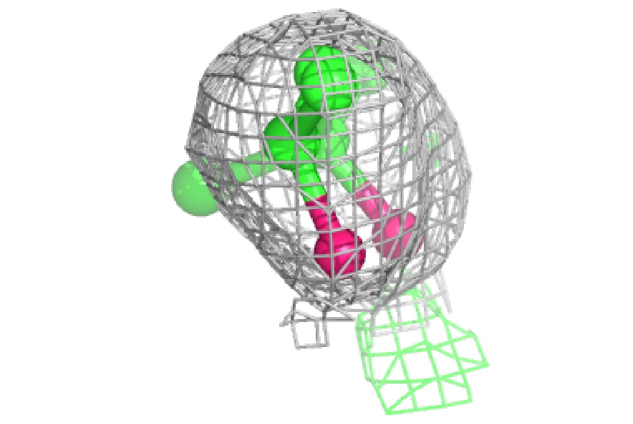

Electron density around FE2 B 1000:

$2mF_o$-DF_c (at 0.7 rmsd) in gray
mF_o-DF_c (at 3 rmsd) in purple (negative)
and green (positive)

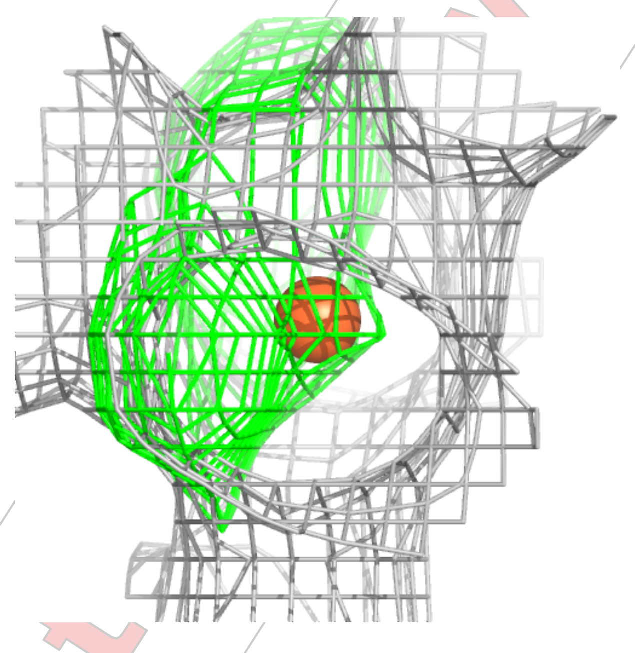

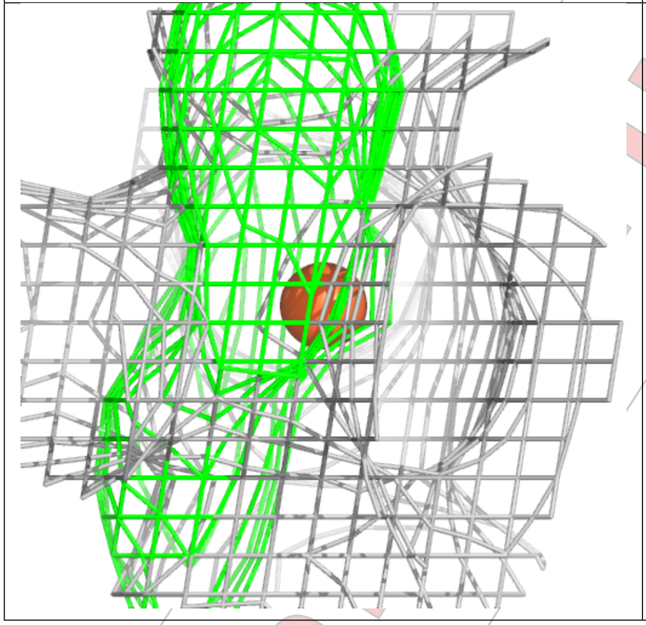

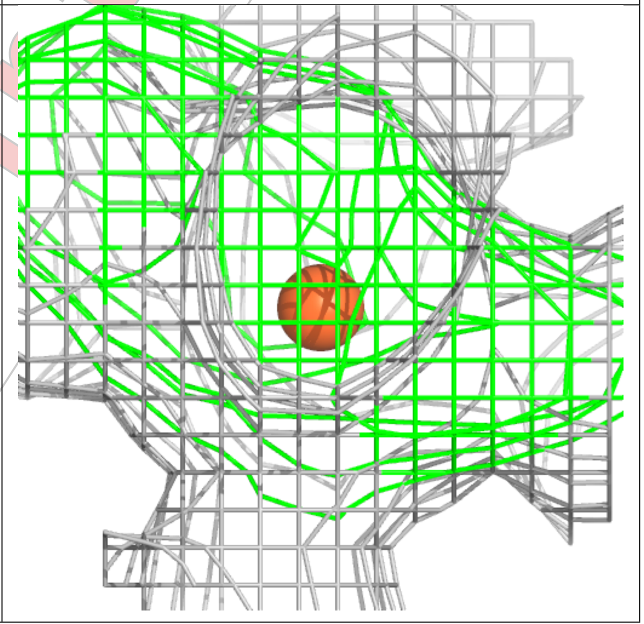

Electron density around FE2 H 1000:

2mF$_o$-DF$_c$ (at 0.7 rmsd) in gray
mF$_o$-DF$_c$ (at 3 rmsd) in purple (negative)
and green (positive)

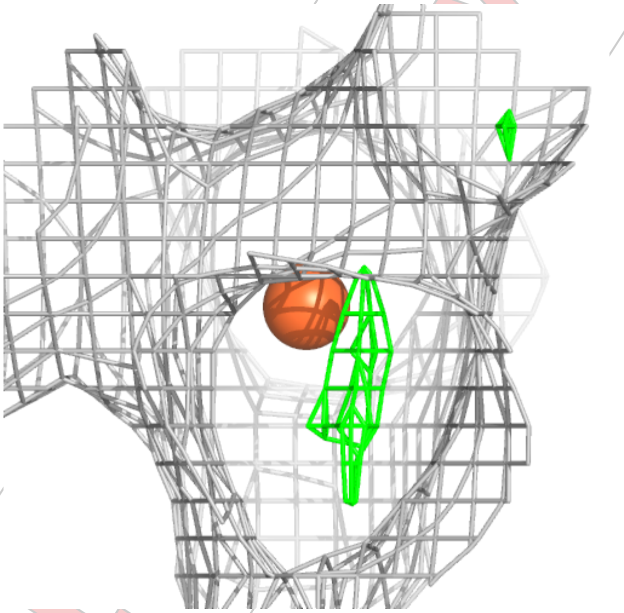

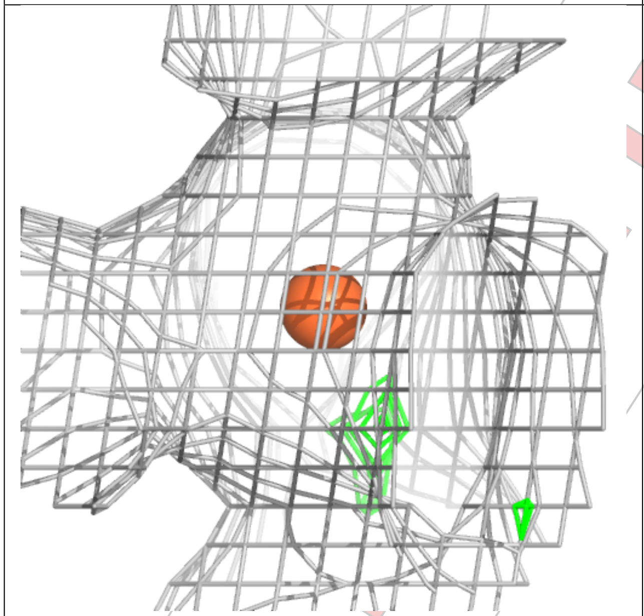

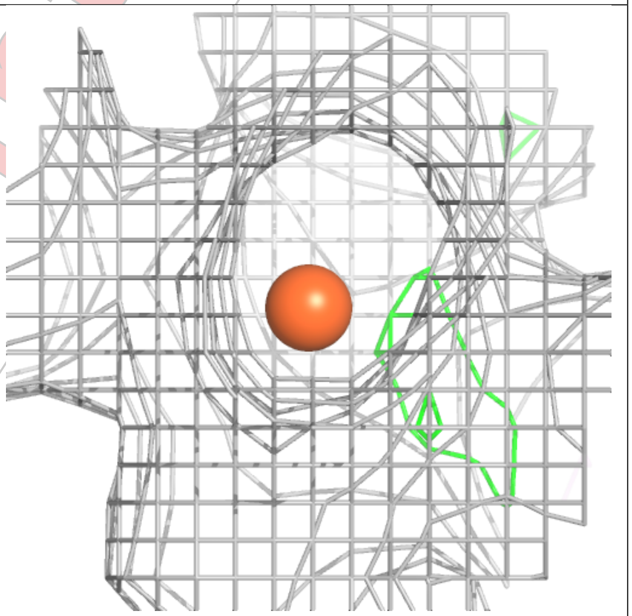

For Manuscript Review

Electron density around FE2 A 601:

$2mF_o$-DF_c (at 0.7 rmsd) in gray
mF_o-DF_c (at 3 rmsd) in purple (negative)
and green (positive)

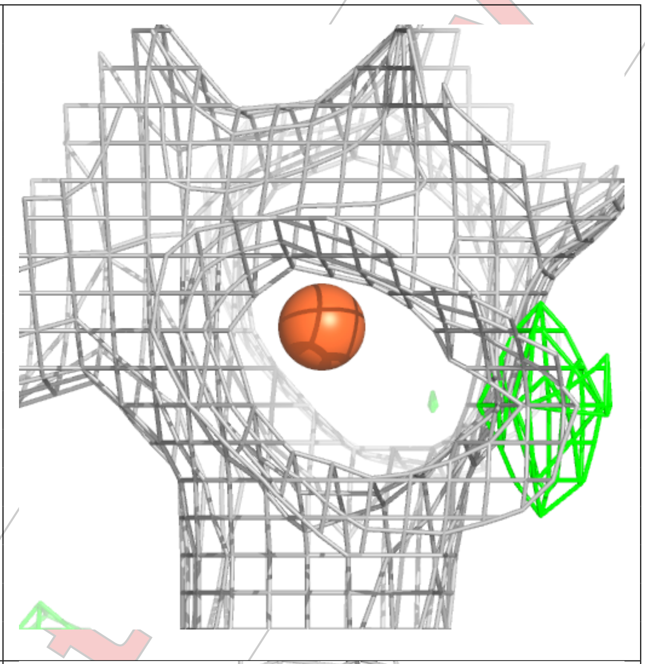

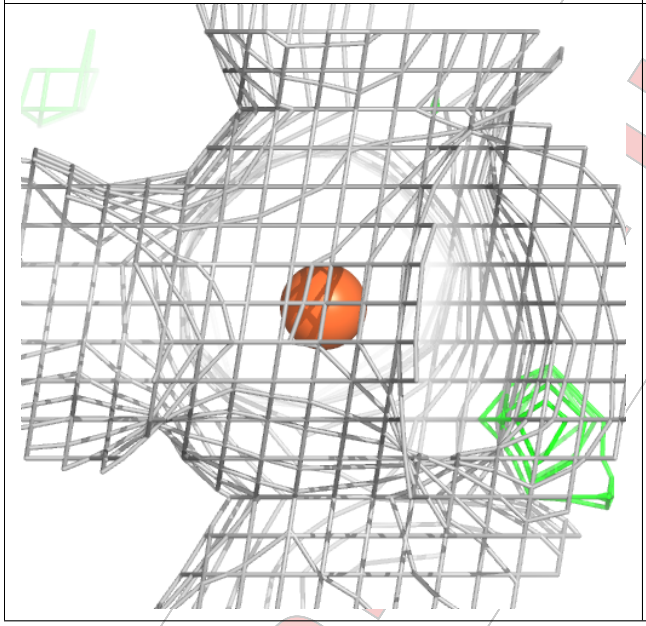

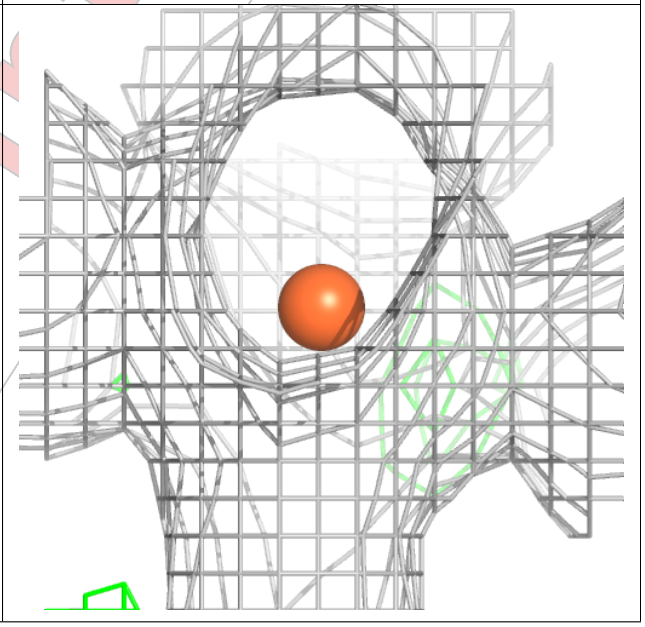

For Manuscript Review

Electron density around FE2 C 601:

$2mF_o$-DF_c (at 0.7 rmsd) in gray
mF_o-DF_c (at 3 rmsd) in purple (negative)
and green (positive)

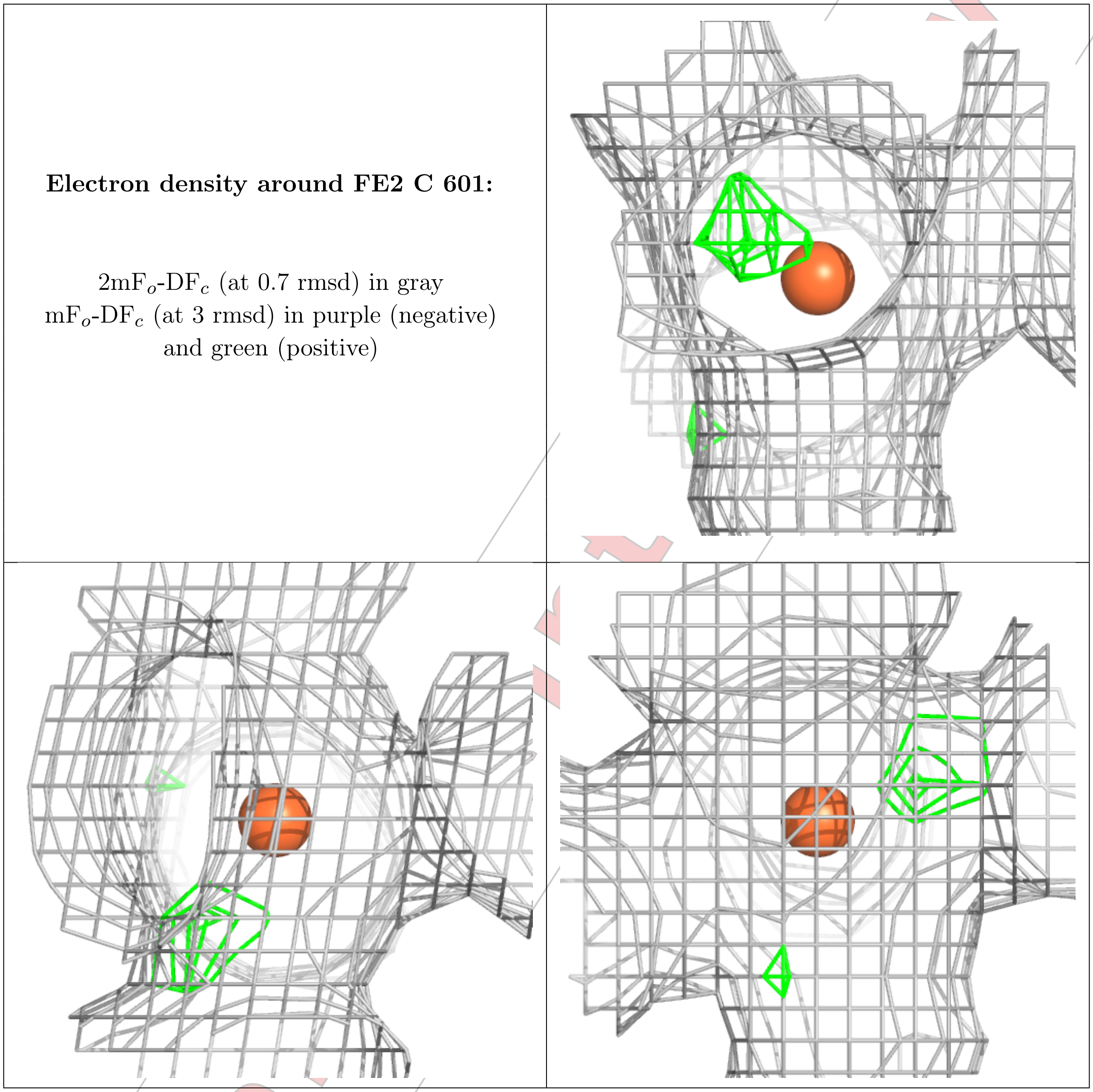

For Manuscript Review

Electron density around FE2 D 601:

2mF$_o$-DF$_c$ (at 0.7 rmsd) in gray
mF$_o$-DF$_c$ (at 3 rmsd) in purple (negative)
and green (positive)

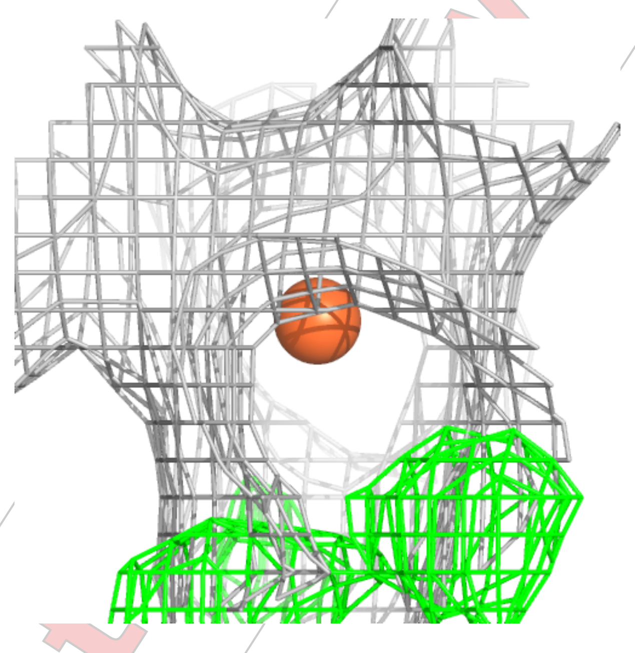

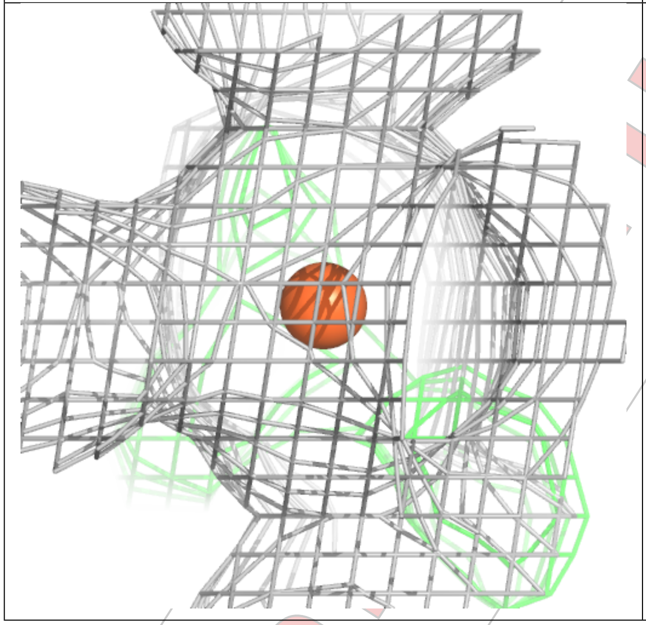

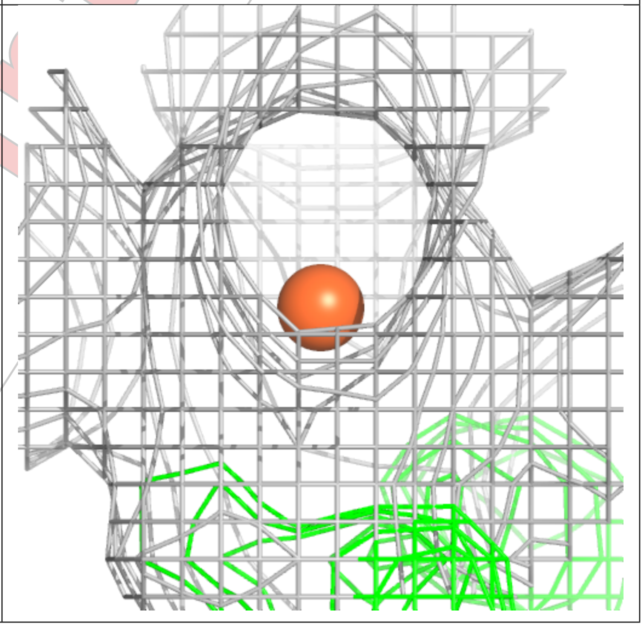

For Manuscript Review

Electron density around FE2 E 601:

$2mF_o$-DF_c (at 0.7 rmsd) in gray
mF_o-DF_c (at 3 rmsd) in purple (negative)
and green (positive)

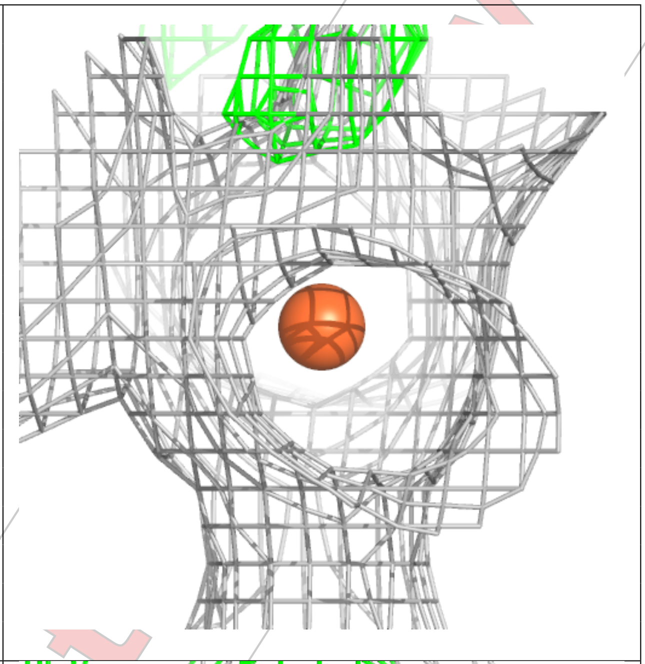

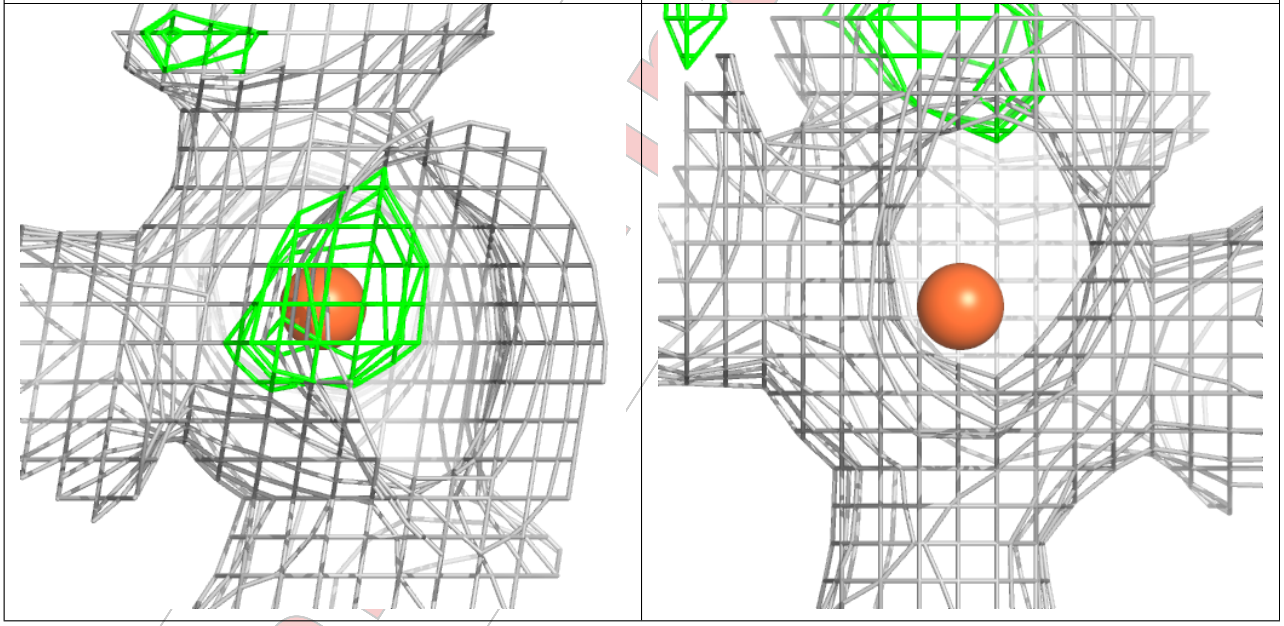

Electron density around FE2 F 1000:

$2mF_o$-DF_c (at 0.7 rmsd) in gray
mF_o-DF_c (at 3 rmsd) in purple (negative)
and green (positive)

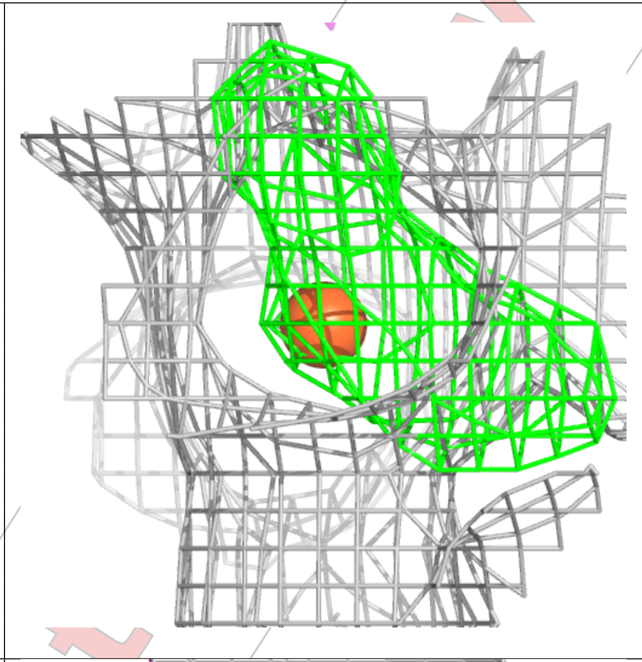

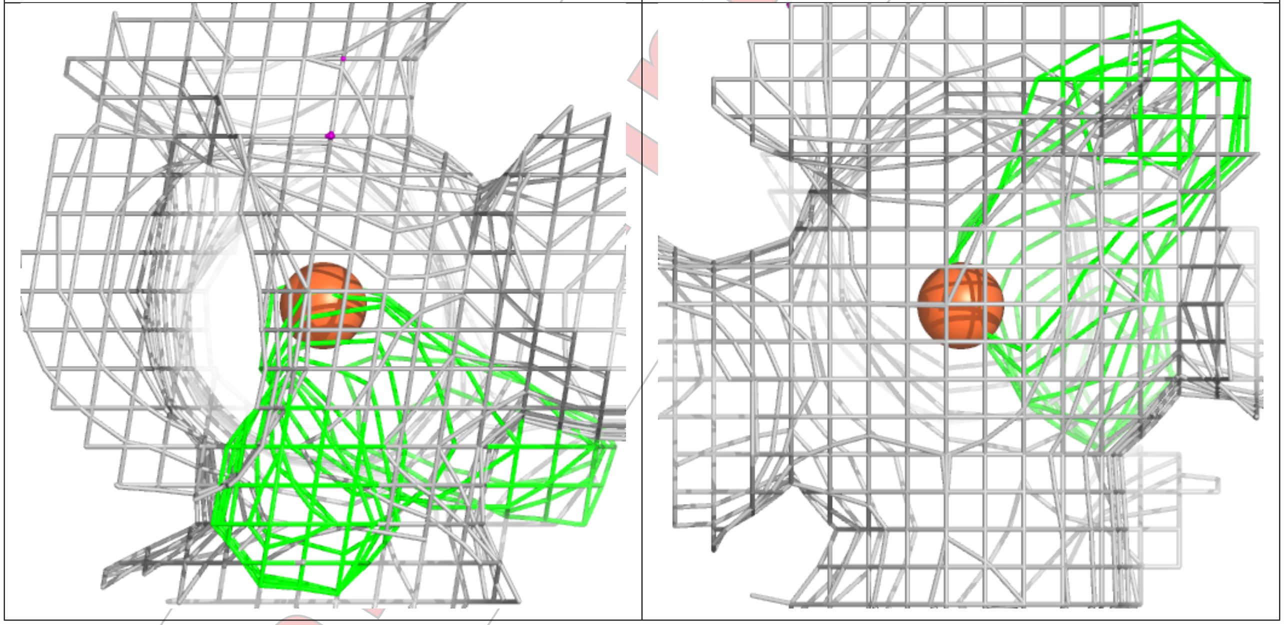

For Manuscript Review

Electron density around FE2 G 601:

2mF$_o$-DF$_c$ (at 0.7 rmsd) in gray
mF$_o$-DF$_c$ (at 3 rmsd) in purple (negative)
and green (positive)

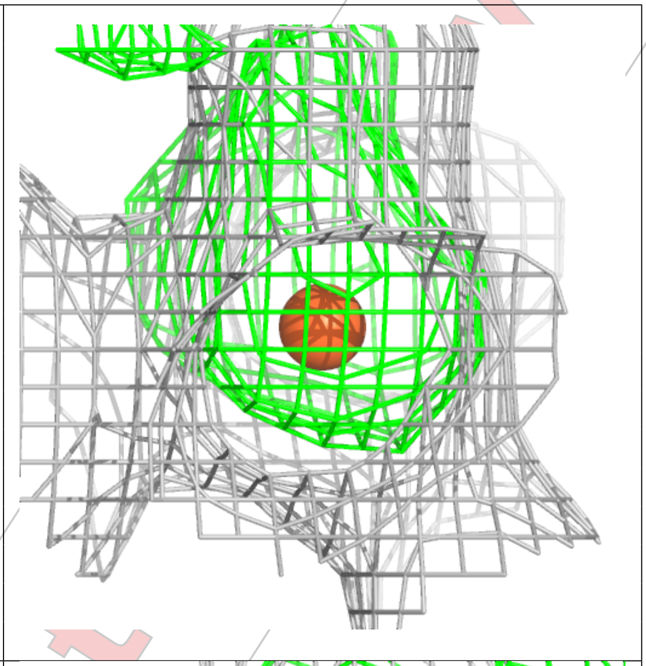

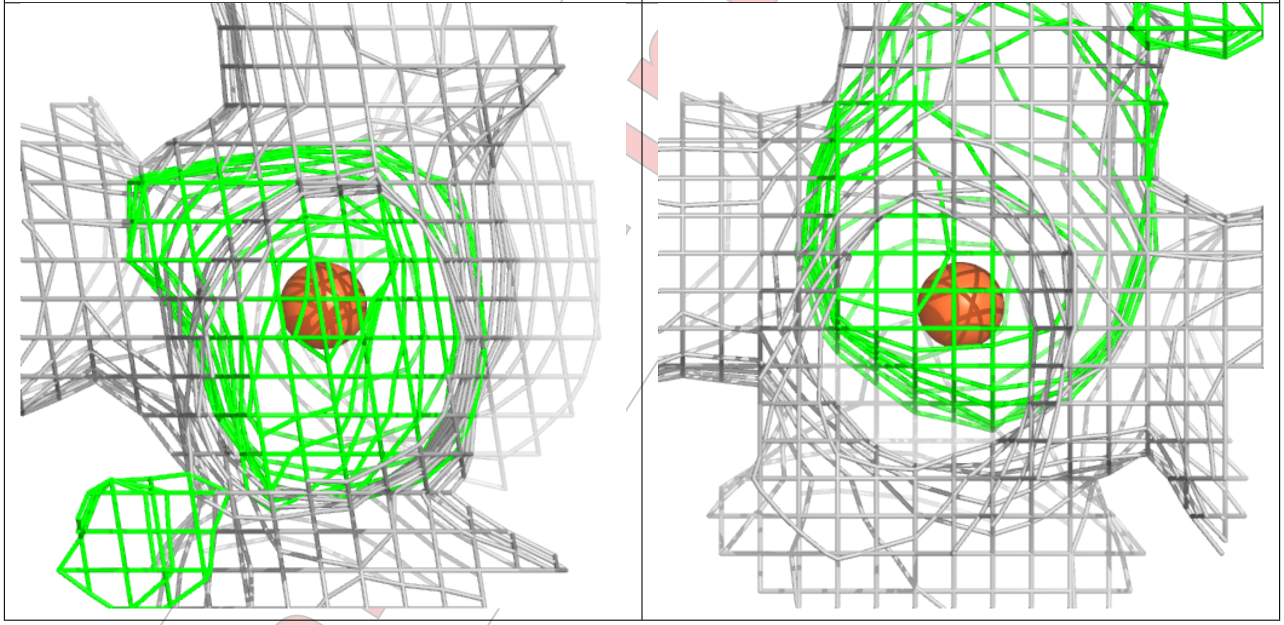

6.5 Other polymers (i)

There are no such residues in this entry.
