# Supplementary figures and images for: Carotenoid cleavage enzymes evolved convergently to generate the visual chromophore
Source: Nat Chem Biol. 2024 Feb 14;20(6):779–88. doi: 10.1038/s41589-024-01554-z (PMC11142922; doi:10.1038/s41589-024-01554-z)

Full gels for Extended Data Figure 1a

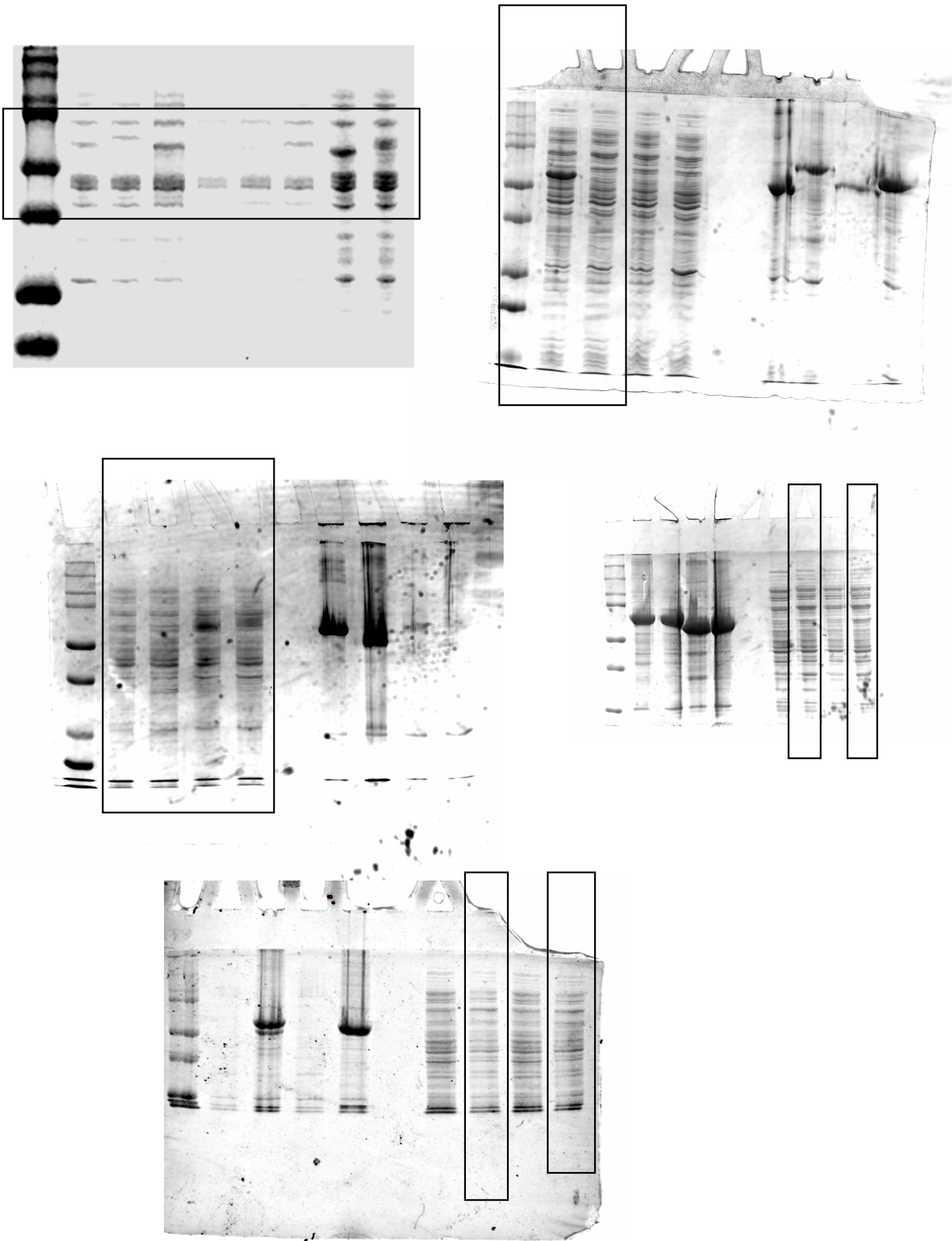

Supplement: Supplementary file 8 — Unprocessed gel images. [file 41589_2024_1554_MOESM8_ESM.pdf]

Full gels for Extended Data Figure 2

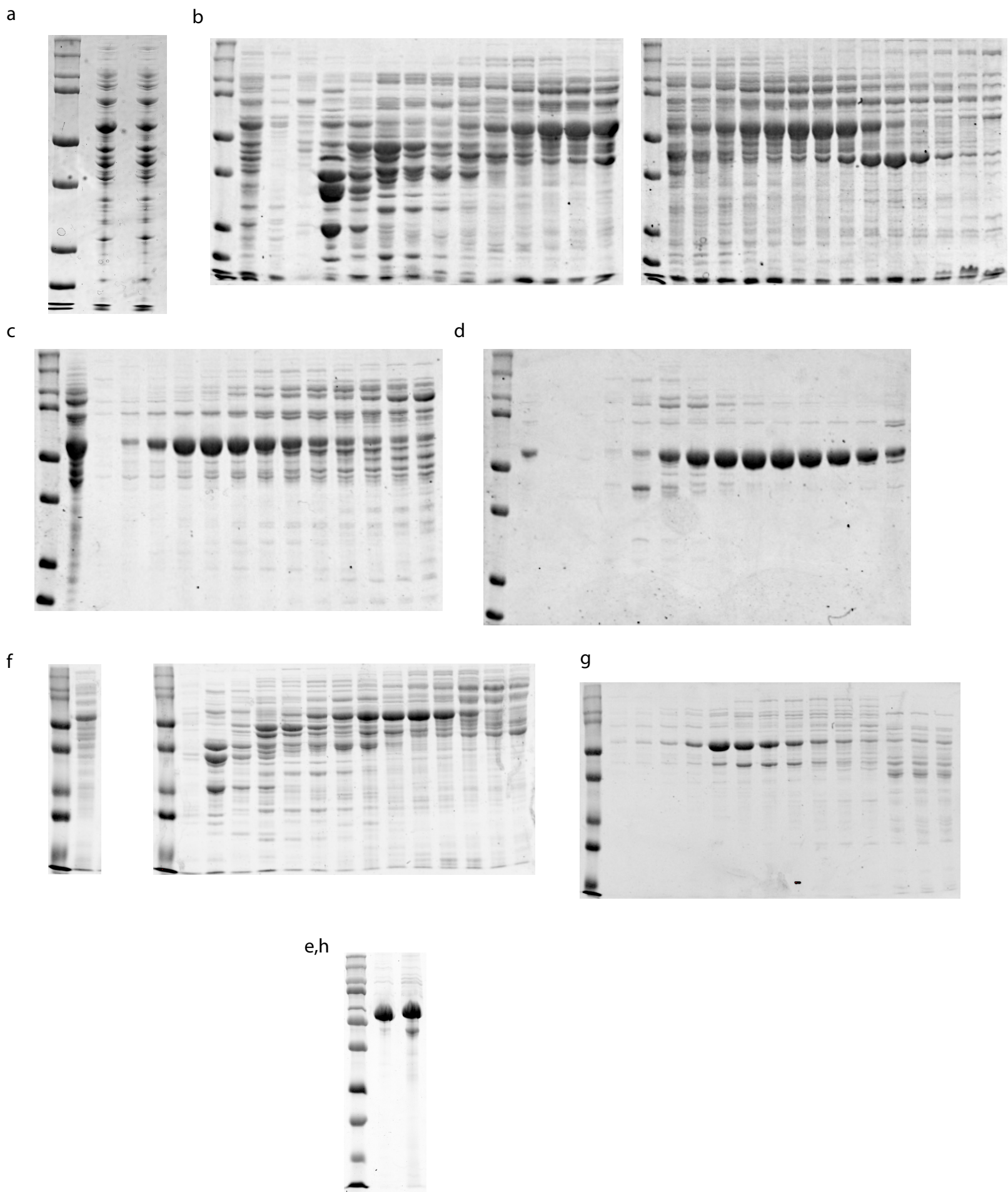

Supplement: Supplementary file 9 — Unprocessed gel images. [file 41589_2024_1554_MOESM9_ESM.pdf]
